# Reduced mortality during the COVID-19 outbreak in Japan, 2020: a two-stage interrupted time-series design

**DOI:** 10.1093/ije/dyab216

**Published:** 2021-10-28

**Authors:** Daisuke Onozuka, Yuta Tanoue, Shuhei Nomura, Takayuki Kawashima, Daisuke Yoneoka, Akifumi Eguchi, Chris Fook Sheng Ng, Kentaro Matsuura, Shoi Shi, Koji Makiyama, Shinya Uryu, Yumi Kawamura, Shinichi Takayanagi, Stuart Gilmour, Takehiko I Hayashi, Hiroaki Miyata, Francesco Sera, Tomimasa Sunagawa, Takuri Takahashi, Yuuki Tsuchihashi, Yusuke Kobayashi, Yuzo Arima, Kazuhiko Kanou, Motoi Suzuki, Masahiro Hashizume

**Affiliations:** 1 Department of Medical Informatics and Clinical Epidemiology, Graduate School of Medical Science, Kyoto Prefectural University of Medicine, Kyoto, Japan; 2 Institute for Business and Finance, Waseda University, Tokyo, Japan; 3 Department of Health Policy and Management, School of Medicine, Keio University, Tokyo, Japan; 4 Department of Global Health Policy, Graduate School of Medicine, The University of Tokyo, Tokyo, Japan; 5 Department of Mathematical and Computing Science, Tokyo Institute of Technology, Tokyo, Japan; 6 Graduate School of Public Health, St. Luke's International University, Tokyo, Japan; 7 Department of Sustainable Health Science, Center for Preventive Medical Sciences, Chiba University, Chiba, Japan; 8 School of Tropical Medicine and Global Health, Nagasaki University, Nagasaki, Japan; 9 Department of Management Science, Graduate School of Engineering, Tokyo University of Science, Tokyo, Japan; 10 HOXO-M Inc., Tokyo, Japan; 11 Department of Systems Pharmacology, Graduate School of Medicine, The University of Tokyo, Tokyo, Japan; 12 Laboratory for Synthetic Biology, RIKEN Center for Biosystems Dynamics Research, Osaka, Japan; 13 Center for Environmental Biology and Ecosystem Studies, National Institute for Environmental Studies (NIES), Tokyo, Japan; 14 RIKEN Center for Sustainable Resource Science, Saitama, Japan; 15 Center for Health and Environmental Risk Research, National Institute for Environmental Studies, Ibaraki, Japan; 16 Department of Statistics, Computer Science and Applications ‘G. Parenti’, University of Florence, Florence, Italy; 17 Infectious Disease Surveillance Center, the National Institute of Infectious Diseases, Tokyo, Japan

**Keywords:** All-cause death, COVID-19, excess mortality, Japan, two-stage interrupted time-series design

## Abstract

**Background:**

Coronavirus disease 2019 (COVID-19) continues to be a major global health burden. This study aims to estimate the all-cause excess mortality occurring in the COVID-19 outbreak in Japan, 2020, by sex and age group.

**Methods:**

Daily time series of mortality for the period January 2015–December 2020 in all 47 prefectures of Japan were obtained from the Ministry of Health, Labour and Welfare, Japan. A two-stage interrupted time-series design was used to calculate excess mortality. In the first stage, we estimated excess mortality by prefecture using quasi-Poisson regression models in combination with distributed lag non-linear models, adjusting for seasonal and long-term variations, weather conditions and influenza activity. In the second stage, we used a random-effects multivariate meta-analysis to synthesize prefecture-specific estimates at the nationwide level.

**Results:**

In 2020, we estimated an all-cause excess mortality of −20 982 deaths [95% empirical confidence intervals (eCI): −38 367 to −5472] in Japan, which corresponded to a percentage excess of −1.7% (95% eCI: −3.1 to −0.5) relative to the expected value. Reduced deaths were observed for both sexes and in all age groups except those aged <60 and 70–79 years.

**Conclusions:**

All-cause mortality during the COVID-19 outbreak in Japan in 2020 was decreased compared with a historical baseline. Further evaluation of cause-specific excess mortality is warranted.

Key Messages

- We estimated the all-cause excess mortality occurring in the COVID-19 outbreak in Japan in 2020 by sex and age group.
- In 2020, we estimated an all-cause excess mortality of −20 982 deaths in Japan, which corresponded to a percentage excess of −1.7% relative to the expected value.
- Reduced deaths were observed for both sexes and in all age groups except those aged <60 and 70–79 years.

## Introduction

Since the early reports of an outbreak of severe acute respiratory syndrome coronavirus 2 (SARS-CoV-2) in Wuhan, China, in December 2019, coronavirus disease 2019 (COVID-19) has had global impact, resulting in considerable morbidity, mortality and economic burden.^1^ As of 24 March 2021, the global number of confirmed cases of COVID-19 across the globe stood at 123 902 242, including 2 727 837 deaths.^2^^,^^3^

Estimation of the excess deaths resulting from COVID-19 is an important challenge since it quantifies the overall burden of COVID-19 compared with a baseline level.^4^ Several studies have reported significant excess COVID-19 mortality in the USA and European countries.^4–11^ However, despite early exposure, high population density and ageing, and no strict quarantine or lockdown measures, Japan has had one of the lowest number of COVID-19 deaths in the world.^12^ Although several studies have reported excess mortality during the COVID-19 epidemic in Japan,^13^^,^^14^ these analyses were based on monthly or weekly data on shorter periods (from January to May or July 2020), used different reference periods (mean monthly mortality in 2 years or 3 weeks before and after a certain point for ≤5 years ago) and did not adjust for seasonal influenza activity, ambient temperature and temporal trends and variations in these confounding factors. The pandemic of COVID-19 is ongoing and it is difficult to cover the entire pandemic in Japan. However, to gain a better understanding of the impact of the disease on mortality in Japan, use of more detailed data over a longer period is essential. Additionally, few studies have accounted for weather factors, influenza epidemics, seasonality and long-term trends. These potential biases might affect interpretation of the results and resolving them requires quantification of excess mortality using more precise modelling methods. Understanding patterns of excess mortality in Japan offers an opportunity to identify whether Japan’s unique approach to COVID-19 control, based on limited voluntary lockdowns and case isolation following World Health Organization (WHO) guidelines, is associated with a different pattern of mortality than the approach in hard-hit nations.

Here, we examined overall excess mortality in the COVID-19 outbreak across all 47 prefectures in Japan, with stratification by sex and age group. Assessment of excess mortality was based on official mortality data obtained from the Ministry of Health, Labour and Welfare, Japan.

## Methods

### Data

We obtained the number of daily deaths, with stratification by sex and age group in 2015–2020 in all 47 prefectures of Japan from the Ministry of Health, Labour and Welfare. The data included complete daily totals for 2015–2019 and early-release data of 2020. Vital statistics in Japan are surveyed based on the Family Register Act and all registries of vital statistics are thought to be complete. We also obtained data on average daily temperatures in 2015–2020 from the Japan Meteorological Agency.^15^ For this, data were sourced from one weather station within an urban region of each prefectural capital city. Measurements were made hourly across 24 hours and averaged to yield average daily temperatures. Furthermore, we obtained national surveillance data on weekly influenza cases in 2015–2020 in all 47 Japanese prefectures from the National Institute of Infectious Diseases.^16^ The daily number of influenza cases was calculated by converting weekly surveillance data to daily values using a uniform distribution of events over 1 week. Mortality counts were summed by prefecture in a sex- and age-specific daily time series and were then associated with daily mean temperature and incidence of influenza cases.

### Statistical analysis

We used a two-stage interrupted time-series design to estimate the time-varying excess mortality in Japan during the COVID-19 outbreak in comparison with the pre-outbreak period, which accounted for temporal trends and variations in other risk factors, as detailed elsewhere.^10^ In the first stage, we used a quasi-Poisson-regression model:^17^

$$\log\left[E\left(Y_{it}\right)\right]=\alpha +h_{1}\left(\mathrm{days}\;\mathrm{from}\;\mathrm{first}\;\mathrm{COVID}19\;\mathrm{case};\theta_{i}\right)+\mathrm{date}+h_{2}\left(\mathrm{day}\;of\;\mathrm{the}\;\mathrm{year};\gamma_{i}\right)+\mathrm{dow}+f\left(T_{it},\ell ;\beta_{i}\right)+\mathrm{flu}+\sum_{l=1}^{28}\log(Y_{it-l})$$

where $Y_{it}\;$denotes daily deaths observed at time *t* in prefecture *i.*

The first component *h_1_* (*days from first COVID*19 *case*; *θ_i_*) represents the spline function that models temporal excess mortality associated with the COVID-19 outbreak and was defined using a constrained quadratic B-spline system. Three equally spaced knots were used in the spline terms to control the smoothness for days from 14 January (date of first confirmed COVID-19 case) to 31 December 2020; the choice of the number of internal knots was based on the smallest Quasi-Akaike Information Criterion (QAIC) (Supplementary Table S9, available as Supplementary data at *IJE* online).

To deal with time-varying confounders (or nuisance variables), we included a linear term for date to control for long-term trends, a cyclic cubic B-spline with 5 degrees of freedom (df) for day of the year $h_{2}\left(\mathrm{day}\;of\;\mathrm{the}\;\mathrm{year};\gamma_{i}\right)$ to account for seasonality, as well as dummy indicators for the day of the week (dow) to control for weekly variation in mortality. To control for potential differences in underlying mortality arising from the non-optimal temperature between the pre-outbreak and outbreak periods, we model the complex relationship between temperature and mortality characterized by non-linearity and delayed (lagged) effects along the lag ℓ as a cross-basis term $f\left(T_{it},\ell ;\beta_{i}\right)$ of distributed lag non-linear models.^18^ In the cross-basis parameterization, we considered a natural cubic-spline function for the temperature with three internal knots, set at the 10th, 75th and 90th percentiles of prefecture-specific empirical distributions of the temperature and we considered the lags of ≤21 days to account for the delayed impact of temperature.^19^ Influenza terms with lags of ≤14 days (*flu*) were included to control for potential confounding of influenza epidemics and their delayed effects.^4^^,^^11^ To allow for autocorrelations, an autoregressive term of lagged deaths counts (≤28 days) $\sum_{l=1}^{28}\log(Y_{it-l})\;$was incorporated into the models.^20^ We checked the dispersion parameter (average of 1.066 in our data), model residuals, observed and fitted values, autocorrelation and partial autocorrelation function of the residuals to determine adequate adjustment for seasonal trends (Supplementary Figures S1–S4, available as Supplementary data at *IJE* online).

In the second stage, we evaluated prefecture-based coefficients $\theta_{i}$ that characterize the excess mortality during the COVID-19 outbreak using a random-effects multivariate meta-analysis model to synthesize the prefecture-specific estimates of coefficients $\theta_{i}\;$at the nationwide level.^21^ We then calculated the best linear unbiased prediction (BLUP) estimated at the prefecture level ${\hat{\theta }}_{bi}$. The BLUP estimate represents a trade-off between specific-to-prefecture and pooled-among-prefectures associations, which enables areas with small numbers of daily cases to use information obtained from larger populations that share similar characteristics and thus stabilizes the estimates.^22^ The Cochran's Q test, which asymptotically follows the chi-squared distribution with the df of 46, were used to assess the heterogeneity between prefectures. This approach has been widely studied in the two-stage time-series design or individual patient data meta-analysis.^21–24^

Nationwide and prefecture-specific estimates were used to calculate the relative risk (RR) of excess mortality in each prefecture for every day of the outbreak period. In particular, every day of the outbreak period was represented using the quadratic B-spline system and the nationwide and BLUP prefecture-specific estimates applied to the spline values. The obtained predicted values were then exponentiated obtaining the RR of excess mortality for every day of the outbreak period. The daily number of excess deaths was calculated as n*(RR-1)/RR, in which *n* is the number of deaths per day. We calculated empirical confidence intervals (eCIs) with 1000 Monte Carlo simulations established using a multivariate normal distribution for the BLUPs for the reduced coefficients. This approach has been tested in a similar context before.^18^^,^^23^^,^^25^ Stratified analysis was performed by sex and age groups (<60, 60–69, 70–79, 80–89 and ≥90 years).

Statistical analyses were performed using the packages *dlnm and mvmeta* in R 3.6.3 (R Core Team, R Foundation for Statistical Computing, Vienna, Austria).

## Results

Total deaths, estimated excess deaths and percentage excess in mortality during the period 13 February–31 December 2020 in Japan are summarized in Table 1. In this period, 1 190 409 deaths were registered in Japan, with an estimated excess of −20 982 deaths (95% eCI: −38 367 to −5472) relative to the expected baseline mortality. This corresponds to a percentage excess of −1.7% (95% eCI: −3.1 to −0.5), indicating that mortality was slightly lower in 2020 than would have been expected given national trends. Among the 47 prefectures, 46 prefectures had the negative percentage of total excess mortality, with the exception of Hyogo Prefecture [0.2% (95% eCI: −1.0 to 1.3)]. Descriptive statistics before and during the COVID-19 pandemic by prefecture are reported in Supplementary Table S1 (available as Supplementary data at *IJE* online). The number of observed and estimated excess deaths (95% eCI) during the period 13 February–31 December 2020 by prefecture is shown in Supplementary Table S2 (available as Supplementary data at *IJE* online).

**Table 1:** Number of observed and estimated excess deaths (95% empirical confidence interval) during the period 13 February–31 December 2020 in Japan

		Total deaths	Excess deaths	Percentage excess
Total		1 190 409	−20 982 (−38 367 to −5472)	−1.7 (−3.1 to −0.5)
Sex	Males	612 370	−8021 (−15 155 to −1779)	−1.3 (−2.4 to −0.3)
	Females	578 039	−14 561 (−19 506 to −9685)	−2.5 (−3.3 to −1.6)
Age	<60	73 688	2213 (386 to 3895)	3.1 (0.5 to 5.6)
	60–69	98 152	−4662 (−7972 to −1492)	−4.5 (−7.5 to −1.5)
	70–79	248 471	4153 (23 to 8080)	1.7 (0.0 to 3.4)
	80–89	426 982	−11 995 (−18 517 to −6138)	−2.7 (−4.2 to −1.4)
	≥90	343 116	−13 604 (−20 585 to −7189)	−3.8 (−5.7 to −2.1)

In the sex-stratified analysis, there were −8021 excess deaths in men (95% eCI: −15 155 to −1779), corresponding to a percentage excess of −1.3% (95% eCI: −2.4 to −0.3). The same estimate for women was −14 561 (95% eCI: −19 506 to −9685), corresponding to a percentage excess of −2.5% (95% eCI: −3.3 to −1.6).

In the age-stratified analysis, there were 2213 excess deaths (95% eCI: 386 to 3895) in the <60-years age group, −4662 (95% eCI: −7972 to −1492) in the 60– to 69-years age group, 4153 (95% eCI: 23 to 8080) in the 70– to 79-years age group, −11 995 (95% eCI: −18 517 to −6138) in the 80–to 89-years age group and −13 604 (95% eCI: −20 585 to −7189) in the ≥90-years age group. The percentage of excess mortality was 3.1% (95% eCI: 0.5 to 5.6) in the <60-years age group, −4.5% (95% eCI: −7.5 to −1.5) in the 60– to 69-years age group, 1.7% (95% eCI: 0.0 to 3.4) in the 70– to 79-years age group, −2.7% (95% eCI: −4.2 to −1.4) in the 80– to 89-years age group and −3.8% (95% eCI: −5.7 to −2.1) in the ≥90-years age group.

**Figure 1:**
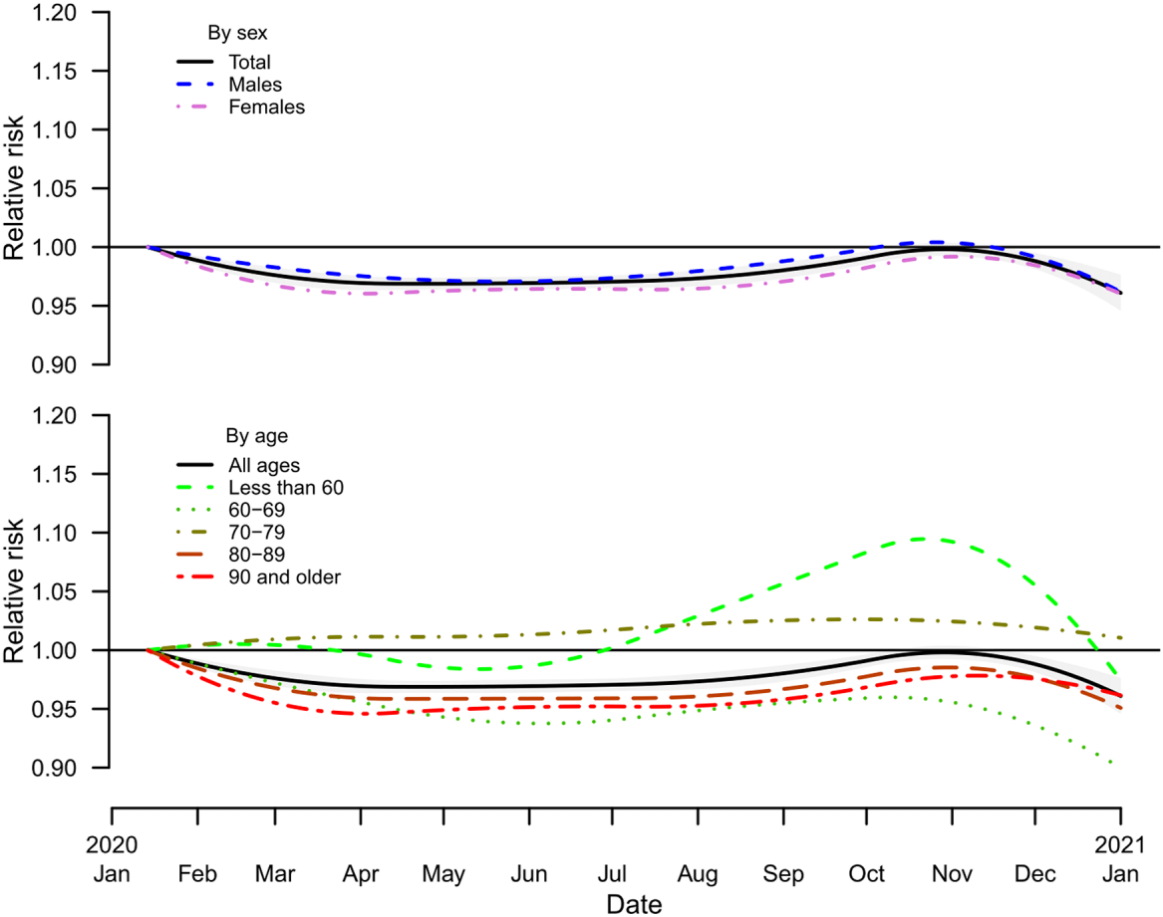
Trends in estimated excess risk (relative risk) during the period 14 January–31 December 2020 in Japan by sex and age groups compared with the total (band corresponds to 95% empirical confidence intervals)

**Figure 2:**
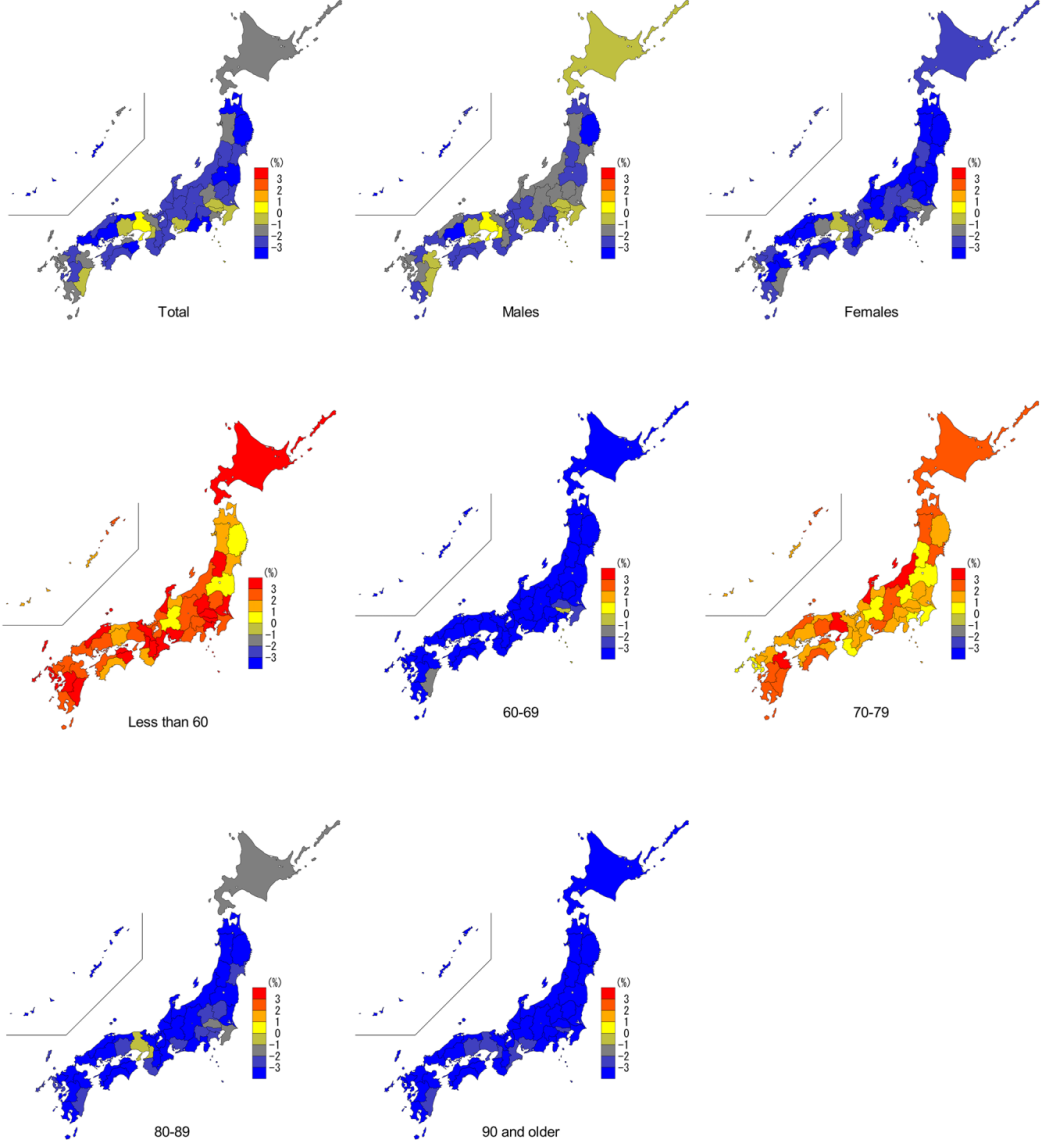
The spatial distribution of percentage excess in mortality during the period 14 January–31 December 2020 in the 47 prefectures of Japan in total and stratified by sex and age groups

Temporal changes in excess risk of mortality during the period 14 January–31 December 2020 in Japan by sex and age group are shown in Figure 1. We found that the total excess risk of mortality started decreasing below the baseline at the beginning of January and continued to remain low. From around September, the total excess risk gradually started increasing and the peak was reached around the beginning of November. The temporal distribution of excess-mortality risk is consistent in both sex and age groups, with an indication of an increase in the <60- and 70– to 79-years age groups. Temporal changes in excess mortality by prefectures and pooled estimates are shown in Supplementary Figure S5 (available as Supplementary data at *IJE* online). Consistently with the country-pooled estimates, similar waveforms were observed for prefecture-specific estimates. The spatial distribution of percentage excess in mortality during the period 14 January–31 December 2020 in the 47 prefectures of Japan in total and stratified by sex and age groups are shown in Figure 2. There was spatial heterogeneity in the excess mortality between prefectures (Cochran Q test, *p* < 0.001; *I*^2^ = 27.3%).

The dispersion parameters, plots of model residuals, observed and fitted time-series plots, autocorrelation and partial autocorrelation function of the residuals suggested that our model had an adequate amount of adjustment for seasonal trends (Supplementary Figures S1–S4, available as Supplementary data at *IJE* online). We also performed sensitivity analyses to evaluate whether these findings were sensitive to the levels of control for influenza activity, ambient temperature and the number of knots (four, five and six) in the interrupted spline component. The sensitivity analysis revealed that the temporal changes in excess mortality were consistent with the main analysis and the obtained curves identify the pandemic as one continuous until November, rather than small waves that clearly depend on the number of knots chosen (Supplementary Figure S6, available as Supplementary data at *IJE* online). The estimates of excess deaths and percentage excess obtained using sensitivity analyses are reported in Supplementary Tables S3–S8 (available as Supplementary data at *IJE* online). The QAICs for the main model and each sensitivity analysis are reported in Supplementary Table S9 (available as Supplementary data at *IJE* online). The sensitivity analysis found that total excess deaths ranged between −28 834 (95% eCI: −33 025 to −24 510) and −18 476 (95% eCI: −24 939 to −11 957), corresponding to a percentage excess of −2.4 (95% eCI: −2.7 to −2.0) and −1.5 (95% eCI: −2.1 to −1.0).

## Discussion

We estimated the all-cause excess mortality during the COVID-19 outbreak in Japan using a two-stage interrupted time-series design and flexible statistical methods. Our models revealed a decrease in mortality during the COVID-19 outbreak in February–December 2020 in Japan. There was significant spatial heterogeneity between prefectures. The difference in the methods between our study and the previous study in Italy is that our study considered the effects of influenza activity in the main model, whereas the previous study did not.

Our findings showed that the COVID-19 outbreak may have potentially led to a decrease in deaths in Japan. These results align with a recent study that indicated a much lower overall excess-mortality burden due to COVID-19 in Japan than in Europe and the USA.^14^ However, that study used weekly data on a shorter period and used data for 3 weeks before and after a certain point as the reference period. Additionally, the study did not consider variations in the effect of influenza activity and ambient temperature.^14^ In contrast, an advantage of our study is that we used an advanced two-stage interrupted time-series design based on daily data and considering temporal trends and variations in these confounding factors. During the first wave of the COVID-19 epidemic in Japan, the Japanese government was criticized because of not performing reverse transcriptase–polymerase chain reaction (RT-PCR) tests extensively^26^ and the very small number of COVID-19 deaths was considered due in part to under-reporting of COVID-19 mortality. However, our results indicated that the total excess risk of mortality in Japan started decreasing below the baseline since the first COVID-19 case was confirmed and continued to remain low. Thus, the criticism that other causes were assigned to the unidentified cases of COVID-19 is not considered to be valid. Our findings suggest that Japan is one of the few countries with negative excess mortality in the world.^27^

Our study revealed significant spatial heterogeneity in excess mortality among prefectures, suggesting the existence of regional differences in the COVID-19 situation. It is possible that regional differences may be related to changes in people's lifestyles, human behaviour and morbidity due to movement restrictions and socio-economic conditions during the COVID-19 outbreak.^28^ Regional variations in excess mortality may be attributed to other factors such as population density, timely access to medical care and available beds, and cluster outbreaks in elderly-care facilities and medical institutions.^29^ These factors may have contributed to the variations in excess mortality and further studies are essential to consider a variety of socio-economic and demographic factors in different regions.

Several possible mechanisms have been proposed to explain this low mortality. First, influenza activity has been at lower levels in 2020 than in previous years in Japan.^30^ Approximately 2000 and 12 000 fewer deaths from influenza and pneumonia without COVID-19, respectively, have been reported in 2020 compared with the same period in 2019.^31^ Second, the number of road-traffic fatalities decreased from January to September 2020 in Japan, which might be due to the decrease in mobility and traffic volumes on major national highways.^32^ These results might be related to the reduction in all-cause deaths due to COVID-19 in Japan.

Japan saw far fewer cases and a much lower incidence rate of COVID-19 than comparable high-income countries^2^ and its coronavirus response was broadly consistent with WHO best-practice guidelines. This reduced transmission of the virus and relative success in protecting elderly populations from infection may also have driven the low mortality rate. Regarding these points, several hypotheses have been proposed. First, East Asian populations have a higher allele frequency of the angiotensin-converting enzyme-2 (ACE2) variants associated with higher tissue expression of ACE2.^33^ Elevated plasma ACE2 is associated with increased risk of atrial fibrillation, myocardial infarction, coronary artery disease, heart failure and aortic stenosis;^34^ thus, Japanese people may have reduced susceptibility due to increased ACE2-receptor expression. Second, countries including Japan with the mandatory bacille Calmette-Guérin (BCG) vaccination may have reduced mortality for COVID-19 compared with those that do not: among findings, BCG may boost trained immunity^35^ and vaccination might be associated with a decrease in mortality.^36^ The BCG vaccine also promotes the production and maturation of naïve T cells that lead to enhanced long-term trained immune protection against SARS-CoV-2.^37^ Furthermore, small-module BCG mimics such as emetin and lopinavir inhibit the growth of SARS-CoV-2 *in* *vitro*, which may contribute to reduce COVID-19 mortality.^37^ Although the exact mechanisms of remain unclear, it may result from complex and multifactorial interactions among these and other as-yet unidentified factors.

Stratified analysis showed a reduction in mortality in both men and women during the COVID-19 outbreak in Japan. Specifically, the percentage excess was −1.3% for men and −2.5% for women, with approximately twice the decline in women as in men. However, we found increases in mortality in the <60-years age group from the middle of June 2020. This finding is consistent with a recent study which suggested that suicide rates in Japan in 2020 increased in October and November in men and in July through November in women, and that the increase was most pronounced among males aged <30 years and in females aged <30 as well as 30–49 years.^38^ Additionally, on 1 March 2021, the Ministry of Health, Labour and Welfare, Japan, reported that the cumulative number of layoffs and suspensions related to the COVID-19 outbreak had reached 90 185 as of 26 February 2021.^39^ Furthermore, on 16 March 2021, Japan’s National Police Agency reported that juvenile suicides reached a record high of 499 in 2020 since records began to be kept in 1980.^40^ These might be related to the fact that physical isolation and lockdown affect serious threats to the mental health and well-being of the general population.^41^ Public health emergencies due to the outbreak are associated with a range of psychosocial difficulties, including economic and financial loss from unemployment and reduced income, school and work closures, inadequate resources for suitable medical responses, domestic violence and insufficient distribution of basic necessities.^42^ Loneliness as well as social isolation also increases the risk of depression, and children and adolescents are more likely to experience increased rates of depression and anxiety.^43^ Although we could not access individual data on deaths, increased suicidality due to economic and mental health problems in the <60-years age group might be related to the increase in mortality.

Our study also showed that there were significant increases in mortality in the 70– to 79-year age group from February 2020 in Japan. Loneliness and social isolation in the aged are also serious public health risks.^44^ Previous studies have shown that older adults are at elevated risk for morbidity and mortality due to COVID-19 and also likely to experience extended isolation.^45^ Social loneliness and isolation in older adults were associated with a 50% increase in the risk of dementia^46^ and a 26% increase in the risk of premature death from all causes.^47^ Moreover, degraded social relationships due to social loneliness and isolation were associated with a 30% increased risk of coronary artery disease and stroke.^48^^,^^49^ These results suggest that increased risk of dementia, coronary heart disease and stroke due to social isolation might be associated with the increase in mortality in the 70– to 79-years age group. Although increased excess mortality was not observed in the 60– to 69-years age group, this might be due to the fact that many people in their 60s are working on the frontline and are an economically active population.^50^ Furthermore, depression symptoms were lower among people aged 60–69 years compared with those aged >70 years during the COVID-19 pandemic.^51^ Further more detailed analysis of cause-specific excess mortality is needed to determine the reason for the higher excess mortality in specific age groups during the COVID-19 outbreak in Japan.

Our present results carry practical implications for the clarification or adjustment of estimates for excess deaths in public health policies for COVID-19. Our study estimates a substantial difference in excess mortality compared with other comparable high-income countries. Although the Japanese government does not have legal authority to impose lockdowns or to fine residents who ignore requests to cooperate in preventing the spread of COVID-19, most Japanese people have been following such requests. Our findings are important because there may be no need to impose strict social-distancing and social-isolation measures for COVID-19 in Japan. Further, international fora should be informed that the influence of COVID-19 on mortality varies by country and region.

There are several limitations in this study. First, we could not take account of information concerning individual factors, such as demographics or socio-economic status, due to difficulties in sourcing data. Therefore, we did not account for social or economic vulnerabilities in our estimates of excess mortality. Additionally, investigating the role of social and demographic factors in the spread of COVID-19 would depend on data on behavioural trends in Japan. Because COVID-19 is likely spread through both human mobility and social transmission networks, these clearly require future study. Second, although the vital-statistics survey is based on the Family Register Act and the registries are considered complete, mortality data in 2020 were early-release data and there may be some differences from the actual number of deaths. This happened due to delays in reporting deaths from municipal governments to the national Ministry of Health, Labour and Welfare. This delay might be due to the overload of the health-department workers due to the COVID-19-related work. However, we suggest that this would not result in substantial bias because the death notification must be submitted to the municipal government within 7 days under the Family Register Act in Japan and the degree of delayed reporting is considered to be very low.^14^ Third, biases may have resulted from our inability to consider immunity to or the likelihood of person-to-person transmission as well as variations in symptoms or presentation of COVID-19 in the population at risk. Variations resulting from immunity to as well as the transmissibility of SARS-CoV-2 in the at-risk population may have biased our estimates. Fourth, although we analysed daily data spanning from 2015 to 2020, the pandemic of COVID-19 is ongoing and our data did not represent the full pandemic period in Japan. The modelling accuracy of our study would be improved by a longer study period or more detailed data. These possible biases might have in turn influenced our interpretation of the results and future studies with more precise modelling methods and detailed data are required.

In summary, our present study estimated a reduction in all-cause mortality during the COVID-19 outbreak in 2020 in Japan. Knowledge of the effects of the COVID-19 outbreak on different types of disease and areas is insufficient. Further studies with longer-term estimation of cause-specific excess deaths during the COVID-19 outbreak are warranted.

## Supplementary data

Supplementary data are available at *IJE* online.

## Ethics approval

The contents of this study were approved by the ethics committee of the National Institute of Infectious Diseases under authorization number 1174. As this study was conducted under a retrospective observational design that specifically included de-identified national mortality data, informed consent was not required.

## Funding

This work was supported in part by a grant from the Ministry of Health, Labour and Welfare, Japan [JPMH20HA2007] and the Japan Society for the Promotion of Science (JSPS) KAKENHI [grant numbers JP18K11666, JP19H03900 and JP21K12274]. The funding sources had no role in the study design, data collection, data analysis, data interpretation or preparation of the manuscript.

## Data availability

The mortality data have been obtained through a restricted data-use agreement with the Ministry of Health, Labour and Welfare, Japan, and are therefore not available for public dissemination.

## Supplementary Material

dyab216_Supplementary_File

## References

[1] Guan WJ , Ni ZY, Hu Y et al; China Medical Treatment Expert Group for Covid-19. Clinical characteristics of coronavirus disease 2019 in China. N Engl J Med 2020;382:1708–20. 32109013 10.1056/NEJMoa2002032 PMC7092819

[2] World Health Organization. WHO Coronavirus Disease (COVID-19) Dashboard. 2020. https://covid19.who.int/ (25 March 2021, date last accessed).

[3] Kabir M , Afzal MS, Khan A, Ahmed H. COVID-19 pandemic and economic cost; impact on forcibly displaced people. Travel Med Infect Dis 2020;35:101661. 32272198 10.1016/j.tmaid.2020.101661 PMC7136875

[4] Weinberger DM , Chen J, Cohen T et al Estimation of excess deaths associated with the COVID-19 pandemic in the United States, March to May 2020. JAMA Intern Med 2020;180:1336–44. 32609310 10.1001/jamainternmed.2020.3391 PMC7330834

[5] Aburto JM , Kashyap R, Scholey J et al Estimating the burden of the COVID-19 pandemic on mortality, life expectancy and lifespan inequality in England and Wales: a population-level analysis. J Epidemiol Community Health 2021;75:735–40. 33468602 10.1136/jech-2020-215505 PMC7818788

[6] Chen YH , Glymour MM, Catalano R et al Excess mortality in California during the coronavirus disease 2019 pandemic, March to August 2020. JAMA Intern Med 2021;181:705–07. 33346804 10.1001/jamainternmed.2020.7578 PMC7754079

[7] Faust JS , Krumholz HM, Du C et al All-cause excess mortality and COVID-19-related mortality among US adults aged 25–44 years, March–July 2020. JAMA 2021;325:785–87. 33325994 10.1001/jama.2020.24243 PMC7745134

[8] Norgaard SK , Vestergaard LS, Nielsen J et al Real-time monitoring shows substantial excess all-cause mortality during second wave of COVID-19 in Europe, October to December 2020. Euro Surveill 2021;26:2002023. 10.2807/1560-7917.ES.2021.26.1.2002023 PMC7809719 33446304

[9] Rossen LM , Branum AM, Ahmad FB, Sutton P, Anderson RN. Excess deaths associated with COVID-19, by age and race and ethnicity—United States, January 26–October 3, 2020. MMWR Morb Mortal Wkly Rep 2020;69:1522–27. 33090978 10.15585/mmwr.mm6942e2 PMC7583499

[10] Scortichini M , Schneider Dos Santos R, De' Donato F et al Excess mortality during the COVID-19 outbreak in Italy: a two-stage interrupted time-series analysis. Int J Epidemiol 2021;49:1909–17. 33053172 10.1093/ije/dyaa169 PMC7665549

[11] van Asten L , Harmsen CN, Stoeldraijer L et al Excess deaths during influenza and coronavirus disease and infection-fatality rate for severe acute respiratory syndrome coronavirus 2, the Netherlands. Emerg Infect Dis 2021;27:411–20. 33395381 10.3201/eid2702.202999 PMC7853586

[12] Iwasaki A , Grubaugh ND. Why does Japan have so few cases of COVID-19? EMBO Mol Med 2020;12:e12481. 32275804 10.15252/emmm.202012481 PMC7207161

[13] Yorifuji T , Matsumoto N, Takao S. Excess all-cause mortality during the COVID-19 outbreak in Japan. J Epidemiol 2021;31:90–92. 33132284 10.2188/jea.JE20200492 PMC7738637

[14] Kawashima T , Nomura S, Tanoue Y et al Excess all-cause deaths during coronavirus disease pandemic, Japan, January–May 2020(1). Emerg Infect Dis 2021;27:789–95. 33622468 10.3201/eid2703.203925 PMC7920666

[15] Japan Meteorological Agency. *Search for Historical Weather Data.* 2021. https://www.data.jma.go.jp/obd/stats/etrn/index.php (25 March 2021, date last accessed).

[16] National Institute of Infectious Diseases, Japan. *Infectious Diseases Weekly Report*. 2021. https://www.niid.go.jp/niid/ja/idwr.html (25 March 2021, date last accessed).

[17] Bhaskaran K , Gasparrini A, Hajat S, Smeeth L, Armstrong B. Time series regression studies in environmental epidemiology. Int J Epidemiol 2013;42:1187–95. 23760528 10.1093/ije/dyt092 PMC3780998

[18] Gasparrini A , Guo Y, Hashizume M et al Mortality risk attributable to high and low ambient temperature: a multicountry observational study. Lancet 2015;386:369–75. 26003380 10.1016/S0140-6736(14)62114-0 PMC4521077

[19] Gasparrini A. Modeling exposure-lag-response associations with distributed lag non-linear models. Stat Med 2014;33:881–99. 24027094 10.1002/sim.5963 PMC4098103

[20] Imai C , Armstrong B, Chalabi Z, Mangtani P, Hashizume M. Time series regression model for infectious disease and weather. Environ Res 2015;142:319–27. 26188633 10.1016/j.envres.2015.06.040

[21] Sera F , Armstrong B, Blangiardo M, Gasparrini A. An extended mixed-effects framework for meta-analysis. Stat Med 2019;38:5429–44. 31647135 10.1002/sim.8362

[22] Gasparrini A , Armstrong B, Kenward MG. Multivariate meta-analysis for non-linear and other multi-parameter associations. Stat Med 2012;31:3821–39. 22807043 10.1002/sim.5471 PMC3546395

[23] Gasparrini A , Guo Y, Sera F et al Projections of temperature-related excess mortality under climate change scenarios. Lancet Planet Health 2017;1:e360–67. 29276803 10.1016/S2542-5196(17)30156-0 PMC5729020

[24] Vicedo-Cabrera AM , Sera F, Gasparrini A. Hands-on tutorial on a modeling framework for projections of climate change impacts on health. Epidemiology 2019;30:321–29. 30829832 10.1097/EDE.0000000000000982 PMC6533172

[25] Gasparrini A , Leone M. Attributable risk from distributed lag models. BMC Med Res Methodol 2014;14:55. 24758509 10.1186/1471-2288-14-55 PMC4021419

[26] Sawano T , Kotera Y, Ozaki A et al Underestimation of COVID-19 cases in Japan: an analysis of RT-PCR testing for COVID-19 among 47 prefectures in Japan. QJM 2020;113:551–55. 32573730 10.1093/qjmed/hcaa209 PMC7454847

[27] Islam N , Shkolnikov VM, Acosta RJ et al Excess deaths associated with covid-19 pandemic in 2020: age and sex disaggregated time series analysis in 29 high income countries. BMJ 2021;373:n1137. 34011491 10.1136/bmj.n1137 PMC8132017

[28] Okumura J. Polarized nature of the COVID-19 pandemic in Japan: associations with population age structure and behaviours. Trop Med Health 2021;49:38. 33985579 10.1186/s41182-021-00324-0 PMC8117128

[29] Makiyama K , Kawashima T, Nomura S et al Trends in healthcare access in Japan during the first wave of the COVID-19 pandemic, up to June 2020. Int J Environ Res Public Health 2021;18: 3271. 33809955 10.3390/ijerph18063271 PMC8004161

[30] Sakamoto H , Ishikane M, Ueda P. Seasonal influenza activity during the SARS-CoV-2 outbreak in Japan. JAMA 2020;323:1969–71. 32275293 10.1001/jama.2020.6173 PMC7149351

[31] Ministry of Health, Labour and Welfare, Japan. *Demographic Surveys in Japan*. 2021. https://www.mhlw.go.jp/toukei/list/81-1a.html (25 March 2021, date last accessed).

[32] Nomura S , Kawashima T, Yoneoka D et al Trends in deaths from road injuries during the COVID-19 pandemic in Japan, January to September 2020. Inj Epidemiol 2021;7:66. 33256821 10.1186/s40621-020-00294-7 PMC7703507

[33] Cao Y , Li L, Feng Z et al Comparative genetic analysis of the novel coronavirus (2019-nCoV/SARS-CoV-2) receptor ACE2 in different populations. Cell Discov 2020;6:11. 32133153 10.1038/s41421-020-0147-1 PMC7040011

[34] Beyerstedt S , Casaro EB, Rangel EB. COVID-19: angiotensin-converting enzyme 2 (ACE2) expression and tissue susceptibility to SARS-CoV-2 infection. Eur J Clin Microbiol Infect Dis 2021;40:905–19. 33389262 10.1007/s10096-020-04138-6 PMC7778857

[35] Netea MG , Joosten LA, Latz E et al Trained immunity: a program of innate immune memory in health and disease. Science 2016;352:aaf1098. 27102489 10.1126/science.aaf1098 PMC5087274

[36] Higgins JP , Soares-Weiser K, Lopez-Lopez JA et al Association of BCG, DTP, and measles containing vaccines with childhood mortality: systematic review. BMJ 2016;355:i5170. 27737834 10.1136/bmj.i5170 PMC5063034

[37] Hajjo R , Tropsha A. A systems biology workflow for drug and vaccine repurposing: identifying small-molecule BCG mimics to reduce or prevent COVID-19 mortality. Pharm Res 2020;37:212. 33025261 10.1007/s11095-020-02930-9 PMC7537965

[38] Sakamoto H , Ishikane M, Ghaznavi C, Ueda P. Assessment of suicide in Japan During the COVID-19 pandemic vs previous years. JAMA Netw Open 2021;4:e2037378. 33528554 10.1001/jamanetworkopen.2020.37378 PMC7856546

[39] Ministry of Health, Labour and Welfare, Japan. *Information About the Impact on Employment Caused by the COVID-19*. 2021. https://www.mhlw.go.jp/stf/seisakunitsuite/bunya/koyou_roudou/koyou/koyouseisaku1.html (25 March 2021, date last accessed).

[40] National Police Agency, Japan. *Suicides in 2020*. 2021. https://www.npa.go.jp/news/release/2021/20210314001.html (25 March 2021, date last accessed).

[41] Fiorillo A , Sampogna G, Giallonardo V et al Effects of the lockdown on the mental health of the general population during the COVID-19 pandemic in Italy: Results from the COMET collaborative network. Eur Psychiatry 2020;63:e87. 32981568 10.1192/j.eurpsy.2020.89 PMC7556907

[42] Pfefferbaum B , North CS. Mental health and the Covid-19 pandemic. N Engl J Med 2020;383:510–12. 32283003 10.1056/NEJMp2008017

[43] Loades ME , Chatburn E, Higson-Sweeney N et al Rapid systematic review: the impact of social isolation and loneliness on the mental health of children and adolescents in the context of COVID-19. J Am Acad Child Adolesc Psychiatry 2020;59:1218–39.e3. 32504808 10.1016/j.jaac.2020.05.009 PMC7267797

[44] National Academies of Sciences, Engineering, and Medicine. Social Isolation and Loneliness in Older Adults: Opportunities for the Health Care System. Washington, DC: The National Academies Press, 2020. 32510896

[45] Donovan NJ , Blazer D. Social isolation and loneliness in older adults: review and commentary of a National Academies report. Am J Geriatr Psychiatry 2020;28:1233–44. 32919873 10.1016/j.jagp.2020.08.005 PMC7437541

[46] Kuiper JS , Zuidersma M, Oude Voshaar RC et al Social relationships and risk of dementia: a systematic review and meta-analysis of longitudinal cohort studies. Ageing Res Rev 2015;22:39–57. 25956016 10.1016/j.arr.2015.04.006

[47] Holt-Lunstad J , Smith TB, Baker M, Harris T, Stephenson D. Loneliness and social isolation as risk factors for mortality: a meta-analytic review. Perspect Psychol Sci 2015;10:227–37. 25910392 10.1177/1745691614568352

[48] Valtorta NK , Kanaan M, Gilbody S, Ronzi S, Hanratty B. Loneliness and social isolation as risk factors for coronary heart disease and stroke: systematic review and meta-analysis of longitudinal observational studies. Heart 2016;102:1009–16. 27091846 10.1136/heartjnl-2015-308790 PMC4941172

[49] Holt-Lunstad J , Smith TB. Loneliness and social isolation as risk factors for CVD: implications for evidence-based patient care and scientific inquiry. Heart 2016;102:987–89. 27091845 10.1136/heartjnl-2015-309242 PMC4941164

[50] Glynn JR. Protecting workers aged 60–69 years from COVID-19. Lancet Infect Dis 2020;20:1123. 10.1016/S1473-3099(20)30311-X PMC7162642 32305069

[51] Zach S , Zeev A, Ophir M, Eilat-Adar S. Physical activity, resilience, emotions, moods, and weight control of older adults during the COVID-19 global crisis. Eur Rev Aging Phys Act 2021;18:5. 33648448 10.1186/s11556-021-00258-w PMC7917372

