# Supplementary material for: Reduced mortality during the COVID-19 outbreak in Japan, 2020: a two-stage interrupted time-series design
Source: Int J Epidemiol. 2021 Oct 28;51(1):75–84. doi: 10.1093/ije/dyab216 (PMC8856001; doi:10.1093/ije/dyab216)
Supplement: dyab216_Supplementary_File [file dyab216_supplementary_file.docx]

**Supplementary data**

Supplement to: Onozuka D, et al. Reduced mortality during the COVID-19 outbreak in Japan, 2020: a two-stage interrupted time-series design.

**Protocol S1.** Additional information on the statistical methods.

**Figure S1.** Diagnostics of models: plots of model residuals in the 47 Japanese prefectures. Vertical dashed line represents the date of first confirmed COVID-19 case in Japan.

**Figure S2.** Diagnostics of models: plots of observed and fitted time series of deaths in the 47 Japanese prefectures. Vertical dashed line represents the date of first confirmed COVID-19 case in Japan.

**Figure S3.** Diagnostics of models: plots of autocorrelation function of the residuals in the 47 Japanese prefectures.

**Figure S4.** Diagnostics of models: plots of partial autocorrelation function of the residuals in the 47 Japanese prefectures.

**Figure S5.** Trends in estimated excess risk (relative risk) during the period 14 January–31 December 2020 in Japan by prefectures and pooled estimates (band for pooled estimates corresponds to 95% empirical confidence intervals).

**Figure S6.** Sensitivity analysis (A) without adjusting for influenza activity and ambient temperature; (B) with adjusting for influenza activity; (C) with adjusting for ambient temperature; (D) with 4 knots in the interrupted spline component; (E) with 5 knots in the interrupted spline component; (F) with 6 knots in the interrupted spline component: Trends in estimated excess risk (relative risk) during the period 14 January–31 December 2020 in Japan by sex and age groups, compared with the total (band corresponds to 95% empirical confidence intervals).

**Table S1.** Descriptive statistics before and during COVID-19 pandemic by 47 prefectures in Japan.

**Table S2.** Number of observed and estimated excess deaths (95% empirical confidence interval) during the period 13 February–31 December 2020 by prefecture in Japan.

**Table S3.** Sensitivity analysis without adjusting for influenza activity and ambient temperature: Number of observed and estimated excess deaths (95% empirical confidence interval) during the period 13 February–31 December 2020 in Japan.

**Table S4.** Sensitivity analysis with adjusting for influenza activity: Number of observed and estimated excess deaths (95% empirical confidence interval) during the period 13 February–31 December 2020 in Japan.

**Table S5.** Sensitivity analysis with adjusting for ambient temperature: Number of observed and estimated excess deaths (95% empirical confidence interval) during the period 13 February–31 December 2020 in Japan.

**Table S6.** Sensitivity analysis with 4 knots in the interrupted spline component: Number of observed and estimated excess deaths (95% empirical confidence interval) during the period 13 February–31 December 2020 in Japan.

**Table S7.** Sensitivity analysis with 5 knots in the interrupted spline component: Number of observed and estimated excess deaths (95% empirical confidence interval) during the period 13 February–31 December 2020 in Japan.

**Table S8.** Sensitivity analysis with 6 knots in the interrupted spline component: Number of observed and estimated excess deaths (95% empirical confidence interval) during the period 13 February–31 December 2020 in Japan.

**Table S9.** The Quasi-Akaike Information Criterion (QAIC) for main model and each sensitivity analysis.

**Protocol S1.** Additional information on the statistical methods.

***First stage***

For each prefecture $i$ we modelled the observed daily deaths counts $Y_{it}$ at time *t* with a specific Quasi-Poisson time-series model described by the following equation (1):

$$log\left[ E\left( Y_{it} \right) \right]=\alpha+h_{1}\left( days from first COVID19 case;\theta_{i} \right)+date+h_{2}\left( day of the year;\gamma_{i} \right)+dow+f\left( T_{it}\mathcal{,l;}\beta_{i} \right)+flu+\sum_{l=1}^{28} \log(Y_{it-l})$$

where $E\left( Y_{it} \right)$ denotes expected daily mortality.

The first component $h_{1}\left( days from first COVID19 case;\theta_{i} \right)$ represent the spline function that model temporal excess mortality associated with the COVID-19 outbreak and was defined using a constrained quadratic B-spline system. Three equally-spaced knots were used in the spline terms to control the smoothness for days from January 14 (date of first confirmed COVID-19 case) to December 31 2020).

To deal with time-varying confounders (or nuisance variables) we included a linear term for date to control for long-term trends, a cyclic cubic B-spline with 5 degrees of freedom (df) for day of the year $h_{2}\left( day of the year;\gamma_{i} \right)$ to account for seasonality, as well as dummy indicators for the day of the week (dow) to control for weekly variation in mortality.

To control for potential differences in underlying mortality arising from non-optimal temperature between the pre-outbreak and outbreak periods we model the complex relationship between temperature and mortality characterized by non-linearity and delayed (lagged) effects along the lag ℓ as a cross-basis term $f\left( T_{it}\mathcal{,l;}\beta_{i} \right)$of distributed lag non-linear models (DLNMs). In the cross-basis parametrization we considered a natural cubic spline function for the temperature with three internal knots, set at the 10th, 75th and 90th percentiles of prefecture-specific empirical distributions of the temperature; and we considered the lags of up to 21 days to account for the delayed impact of temperature. Influenza terms with lags up to 14 days (*flu*) were included to control for potential confounding of influenza epidemics and their delayed effects. To allow for autocorrelations, an autoregressive term of lagged deaths counts (up to 28 days) $\sum_{l=1}^{28} \log(Y_{it-l})$was incorporated into the models.

***Second stage***

The set of parameters $\theta_{i}$ characterize on each-prefecture $i$ the spline function modeling the temporal variations in risk associated with the COVID-19 outbreak; fixing a quadratic B-spline system with *k* internal knots, we obtained a set of *k+*2 coefficients vector $\theta_{i}$ , with dimension$((k+2)\times1)$, and their covariance matrix $\boldsymbol{S}_{i}$ , with dimension$((k+2)\times(k+2))$. This set of parameters $\theta_{i}$ was estimated from the model (1) in the first stage in each prefecture $i$ and were combined using the extended random effect meta-analysis that consider possible non-independence of estimates as measured by the covariance matrix $S_{i}$.

The extended framework random effect meta-analysis can be written in general form as:

$\theta_{i}=X_{i}\beta+Z_{i}b_{i}+\varepsilon_{i}$ (2)

with $b_{i}\sim N\left( 0,\Psi\right)$, and $\varepsilon_{i}\sim N\left( 0,S_{i} \right)$.

The matrix $X_{i}$ include fixed-effect predictors. Random terms are represented by the design matrix $Z_{i}$ with random coefficients $b_{i}$. The random coefficients have (co)variance matrices $\Psi$. The term $S_{i}$ represents the covariance matrix of the first-stage estimates $\theta_{i}$.

In the second stage analysis, we performed overall and stratified analysis by age groups and sex. On each analysis, in the second stage model the design matrices of the fixed and random effects are identity matrices with dimensions equal to the number of coefficients: $X_{i}=Z_{i}=I_{k+2}$, that is, this represents an example of multivariate meta-analysis. With this parametrization the fixed-effect coefficient vector $\beta$, with dimension$((k+2)\times1)$, represent the “pooled” coefficients set of the quadratic B-spline system.

***Third stage***

The estimated fixed-effects coefficients $\beta$ from the random effect meta-analysis (2) fitted in second stage were used to calculate nationwide relative risk (RR) of excess mortality for every day of the outbreak period.

From the random effect meta-analysis (2) we estimated the best linear unbiased prediction $\hat{\theta}_{i}$for each prefecture:

$\hat{\theta}_{bi}=\hat{\beta}+Z_{i}\hat{\Psi}Z_{i}^{T}\hat{\Sigma}^{-1}(\theta_{i}-\hat{\beta})$

and these were used to calculate prefecture-specific estimates relative risk (RR) of excess mortality for every day of the outbreak period.

In particular every day of the outbreak period was represented using the quadratic B-spline system and the nationwide and prefecture-specific estimates applied to the transformed values. The obtained predicted values were then exponentiated obtaining the relative risk (RR) of excess mortality for every day of the outbreak period.

The daily number of excess deaths was calculated as *n*(RR-1)/RR*, in which n is the number of deaths per day. We calculated empirical confidence intervals (eCIs) with 1000 Monte Carlo simulations established using a multivariate normal distribution for the best linear unbiased predictions for the reduced coefficients. Stratified analysis was performed by sex and age groups (<60 years, 60–69, 70–79, 80–89, and ≥90 years).

**Figure S1.** Diagnostics of models: plots of model residuals in the 47 Japanese prefectures. Vertical dashed line represents the date of first confirmed COVID-19 case in Japan.


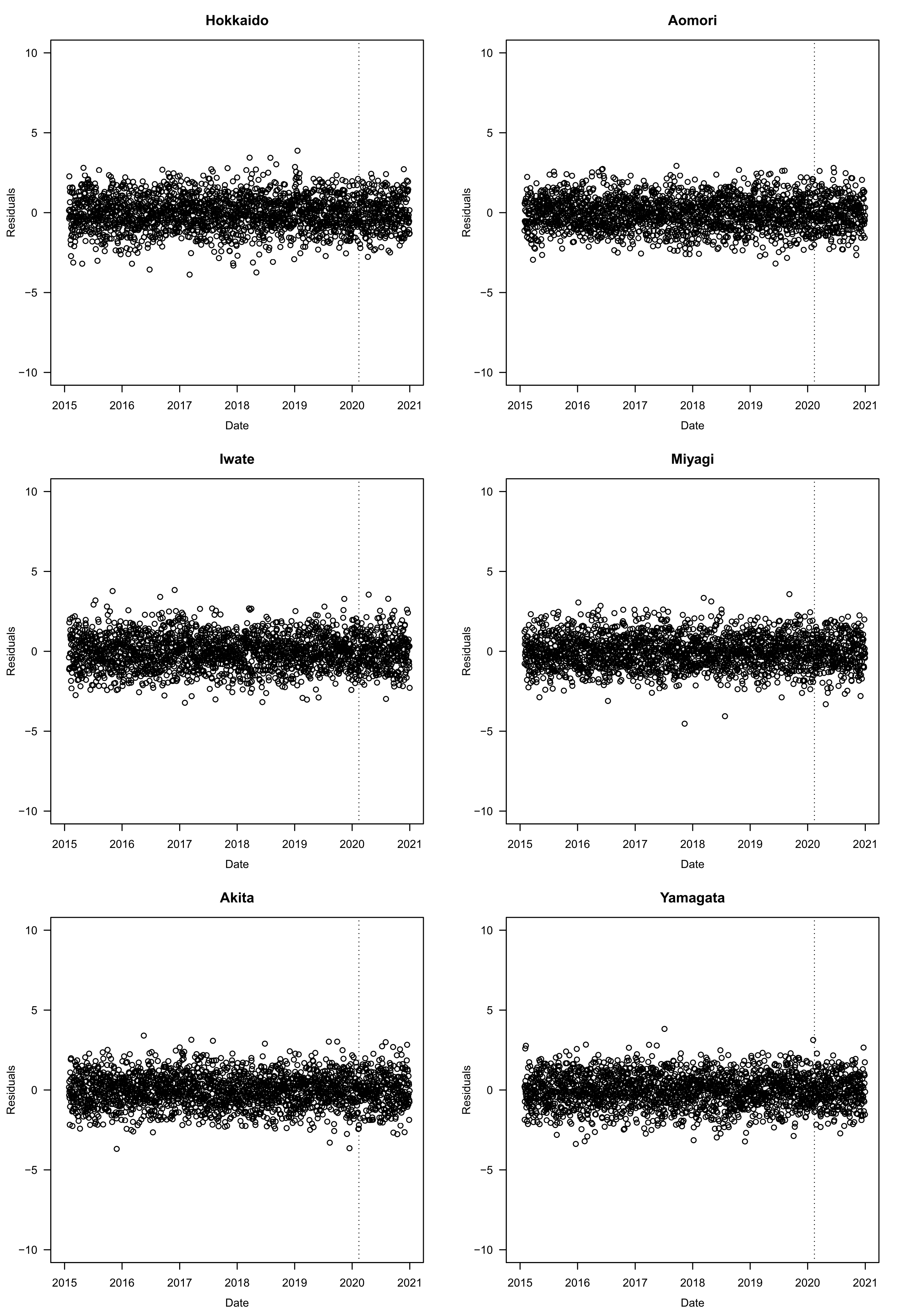


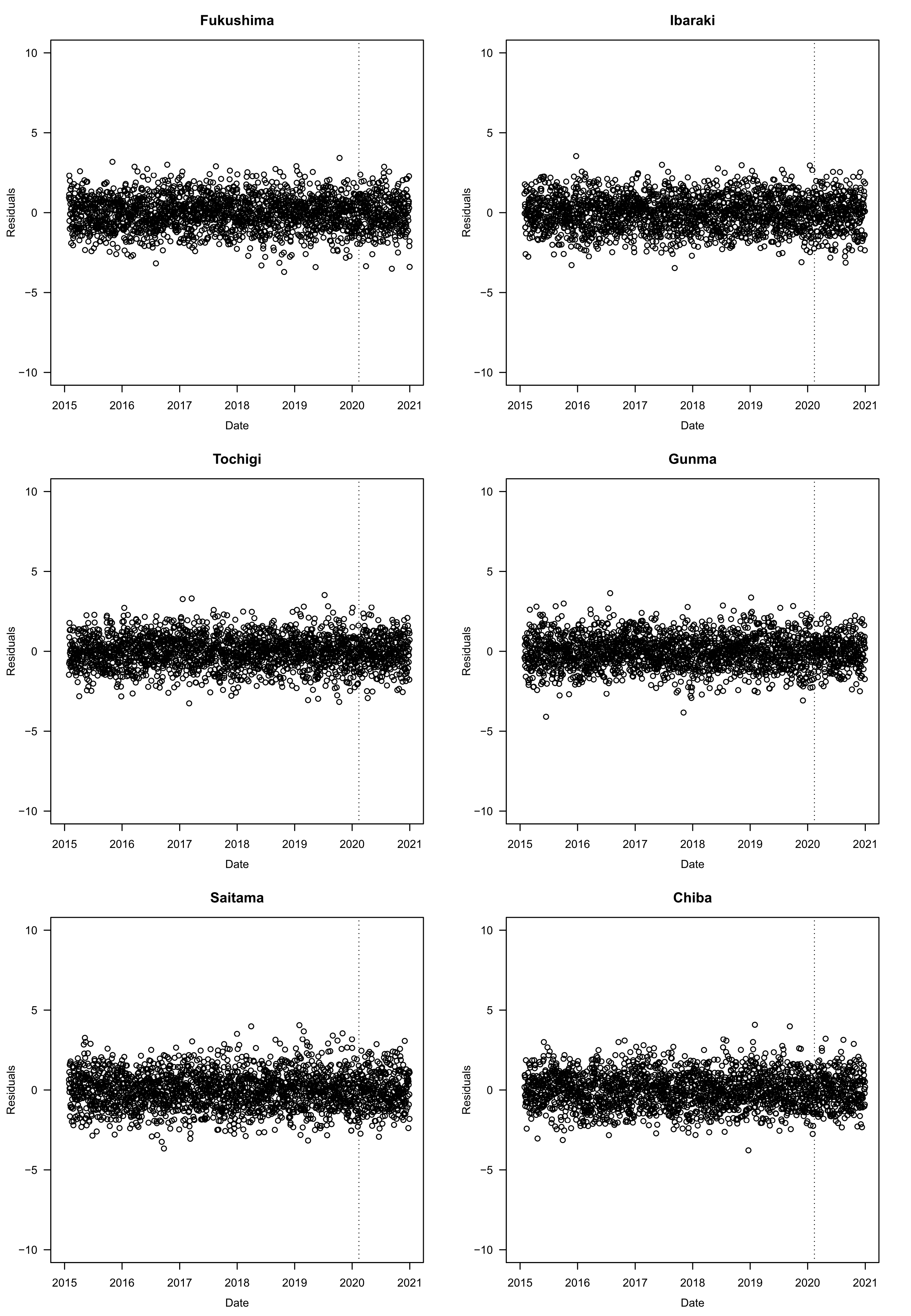


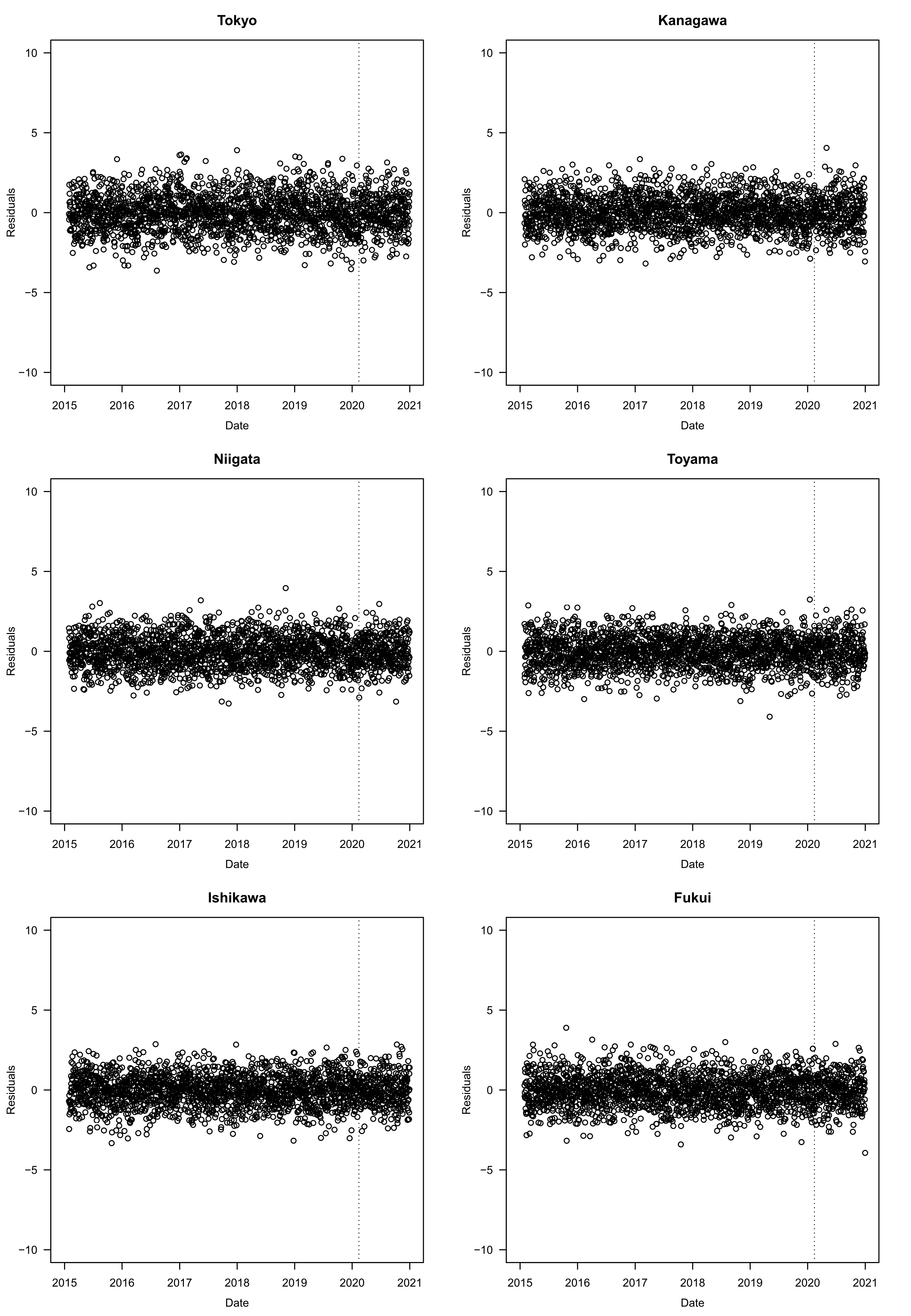


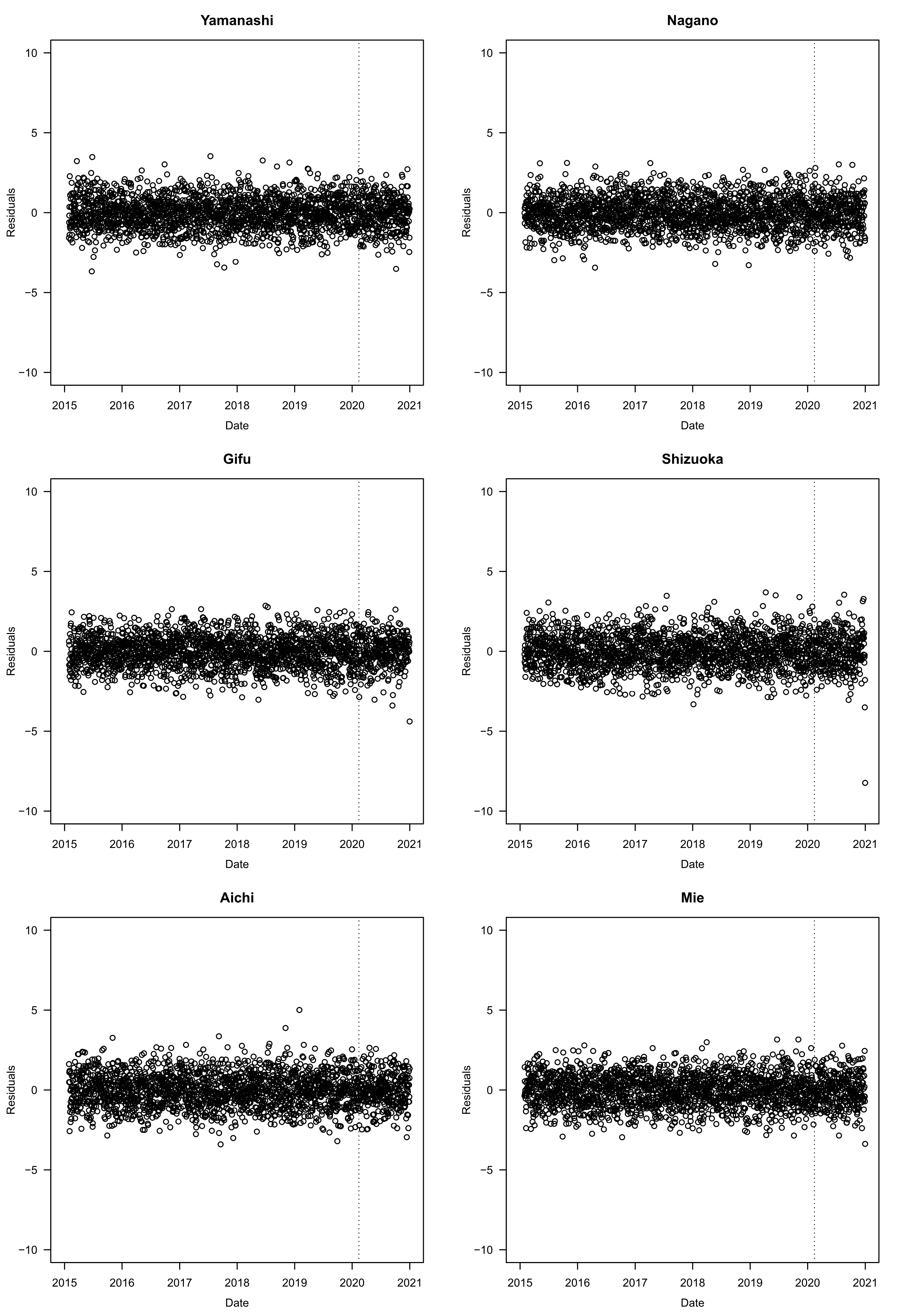


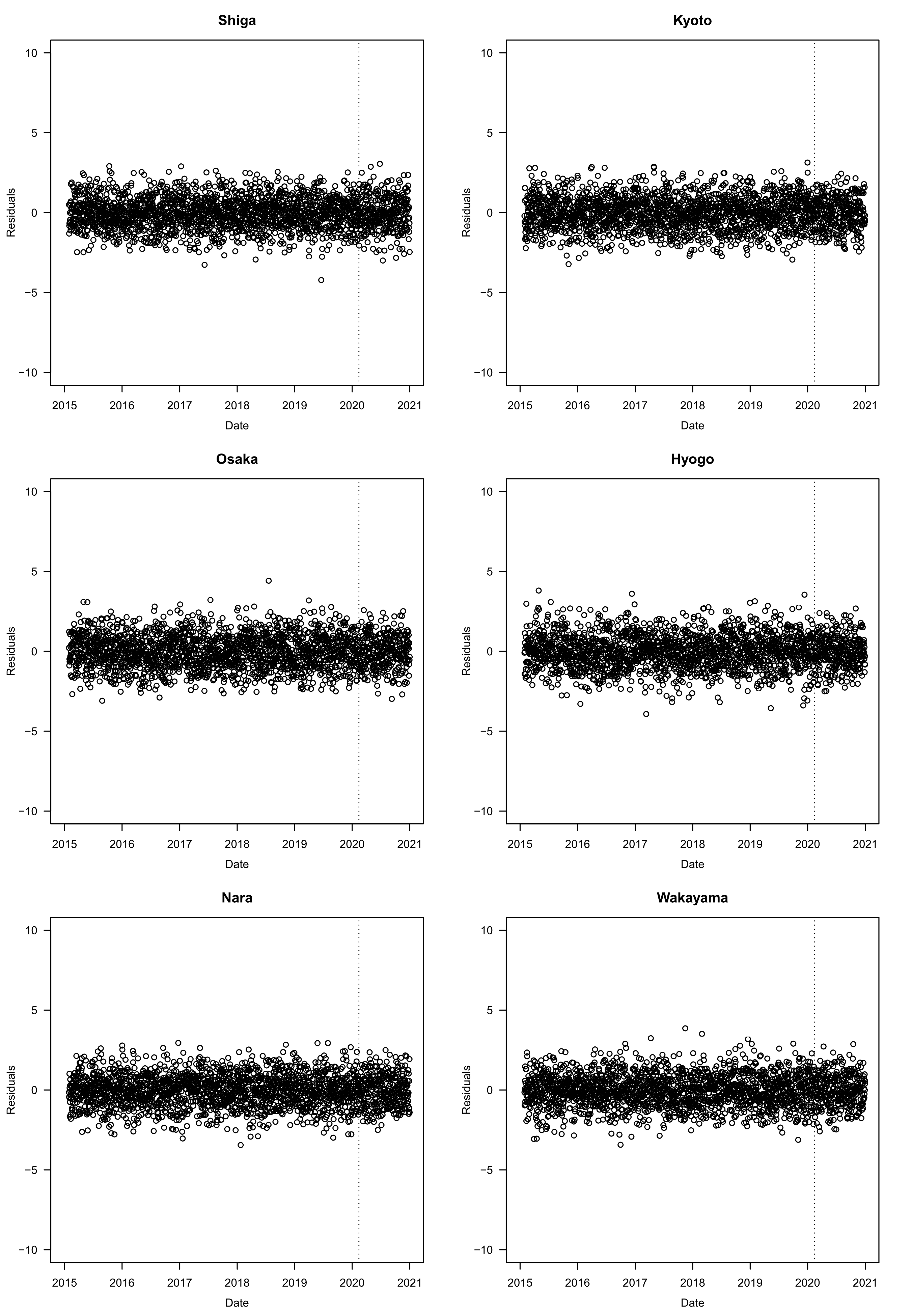


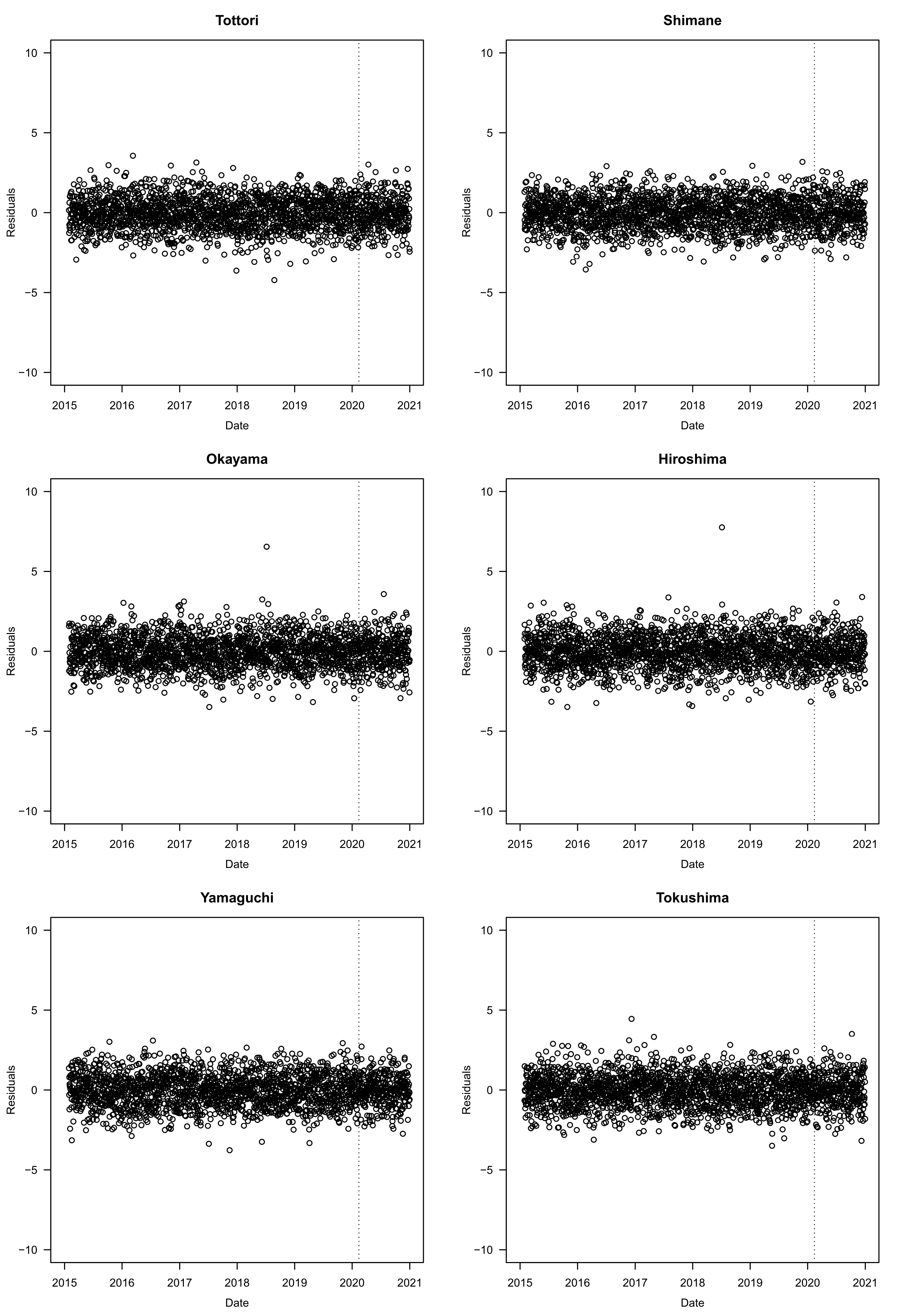


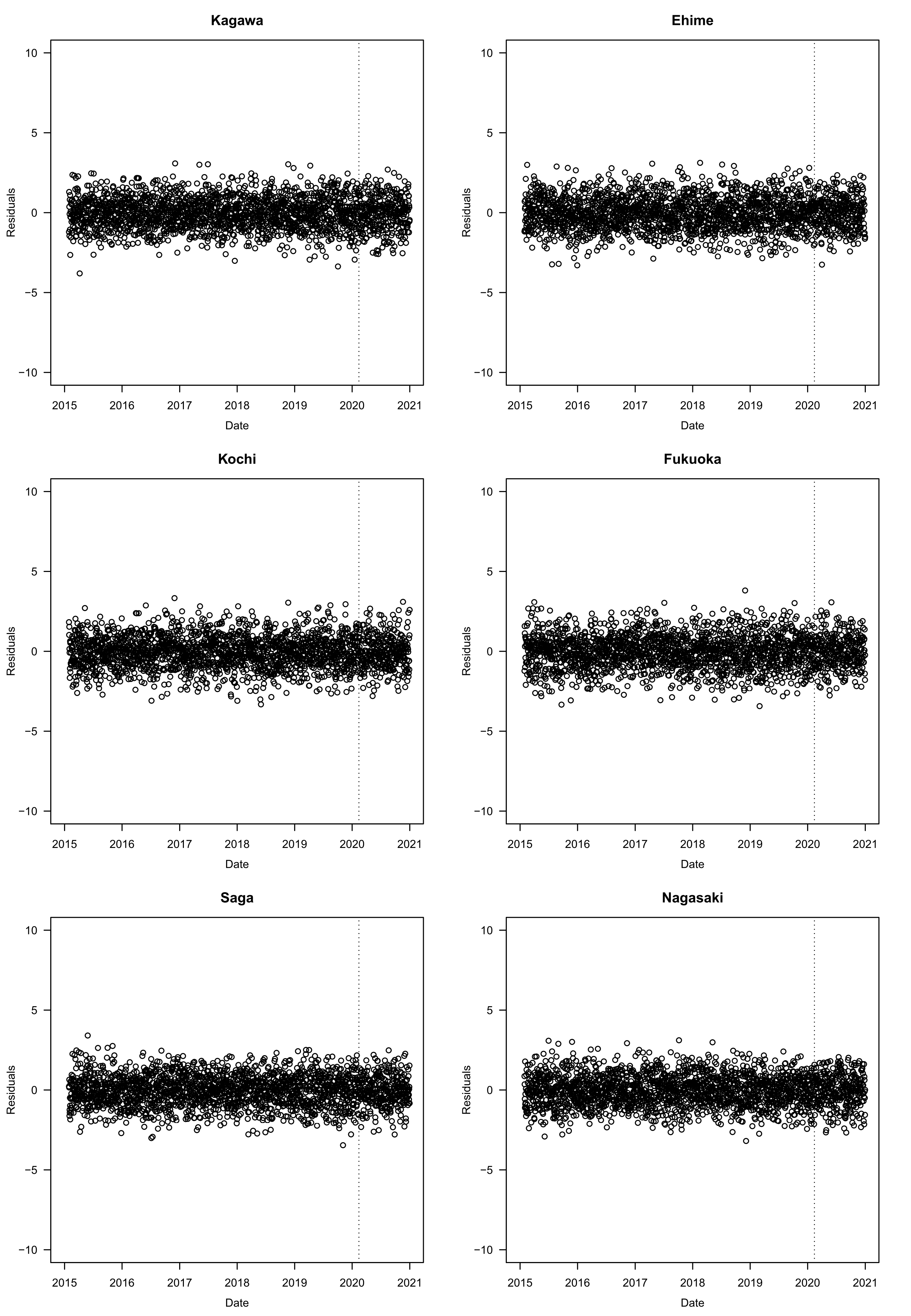


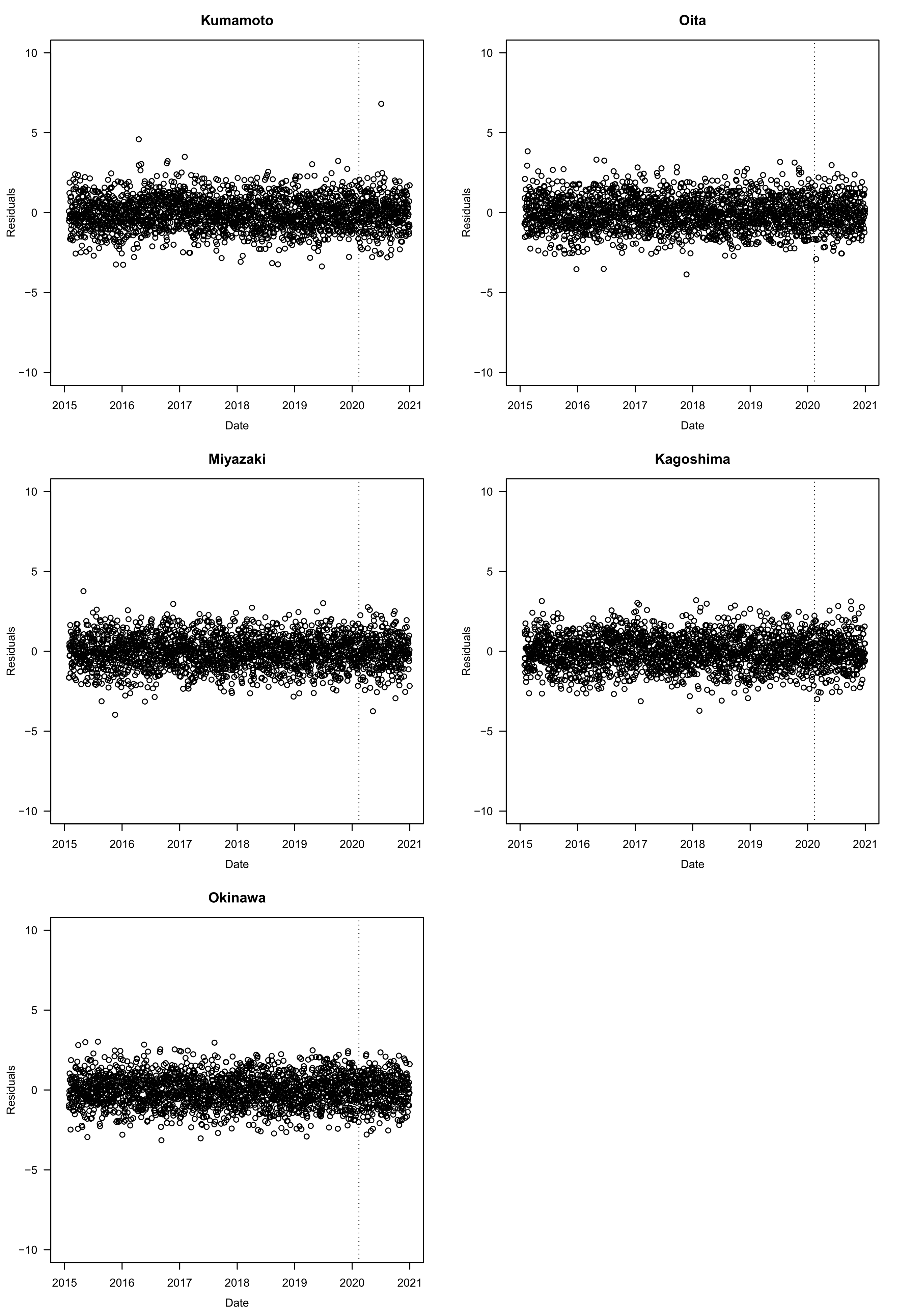


**Figure S2.** Diagnostics of models: observed and fitted time series plots of deaths in the 47 Japanese prefectures. Vertical dashed line represents the date of first confirmed COVID-19 case in Japan.


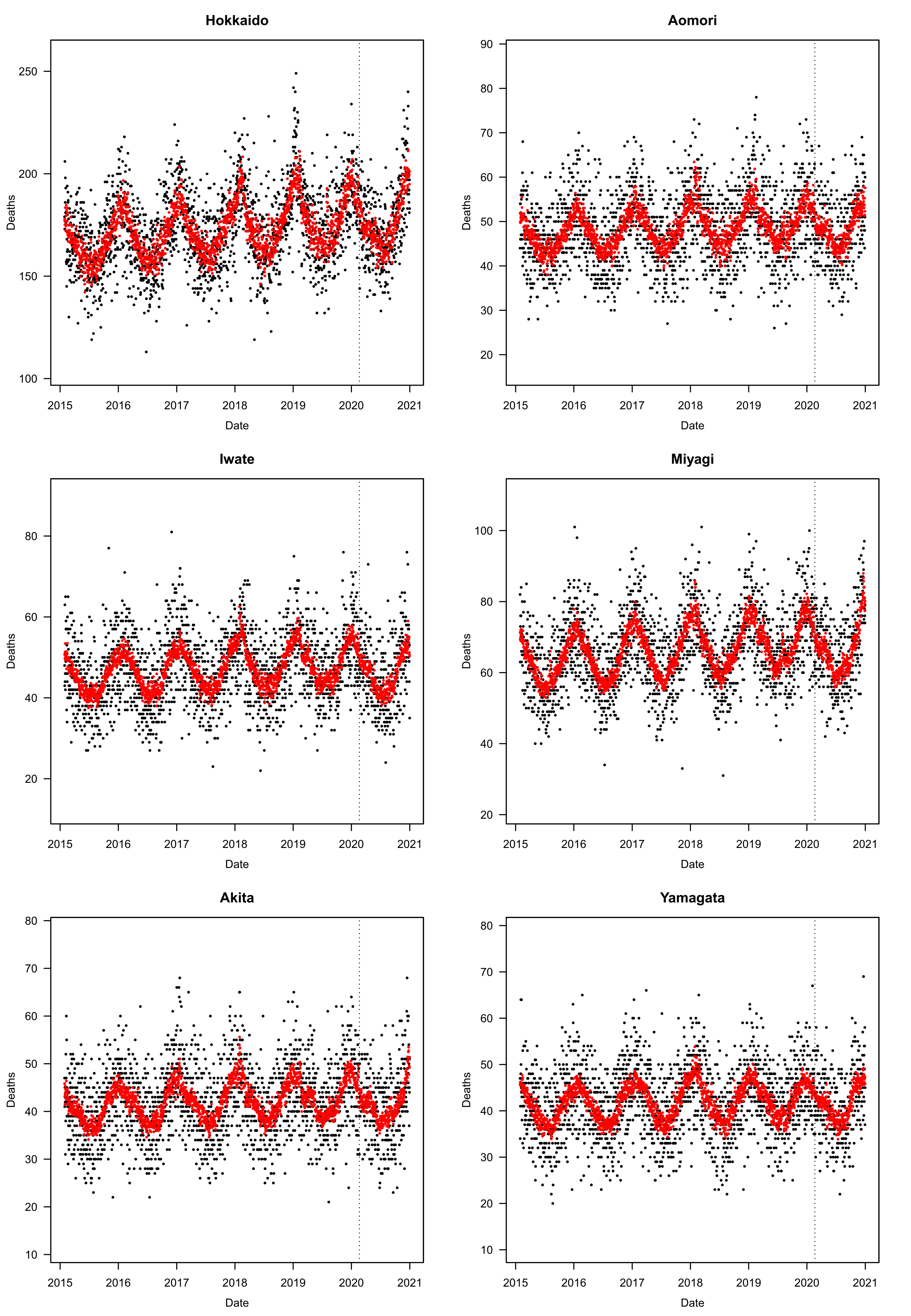


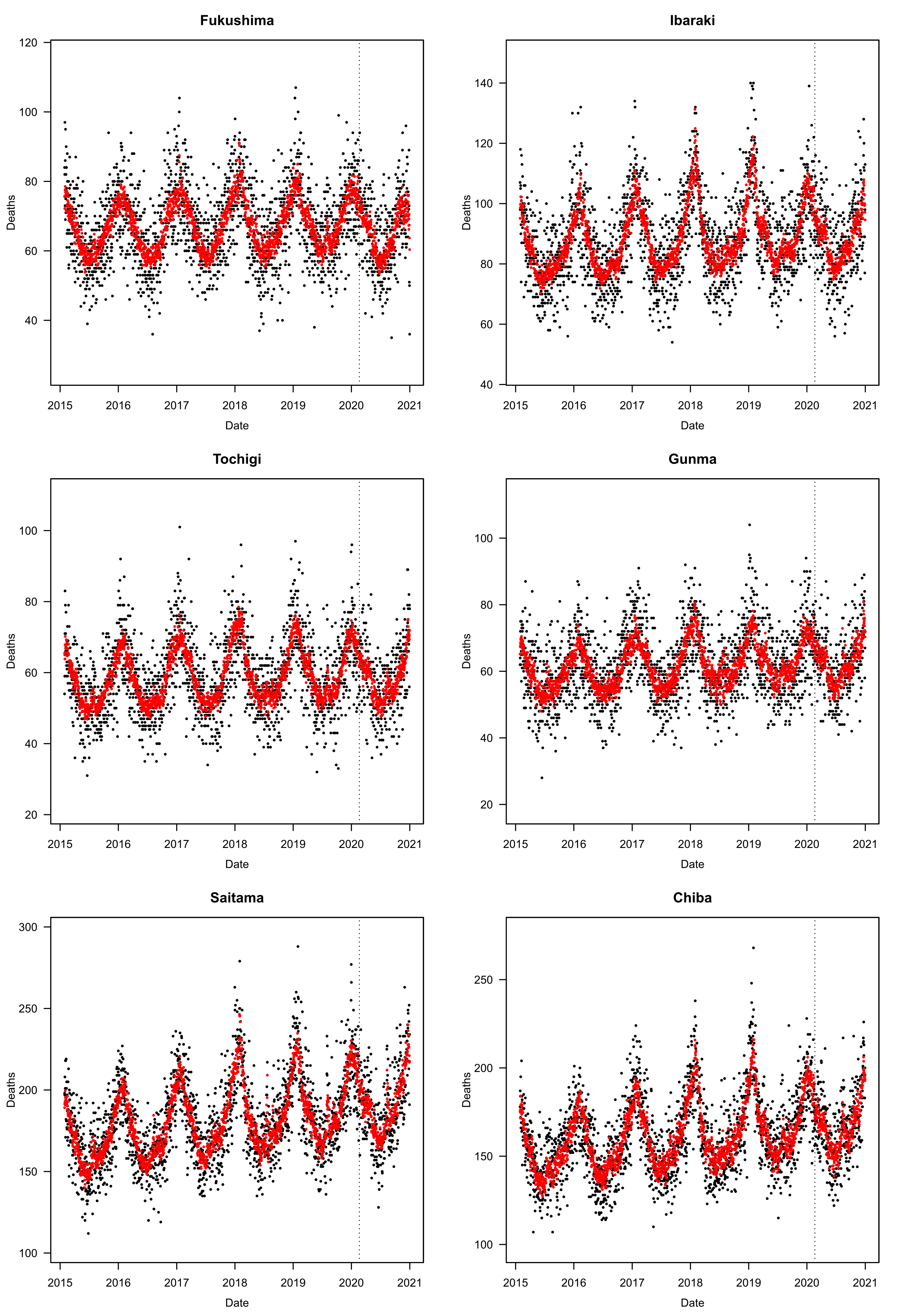


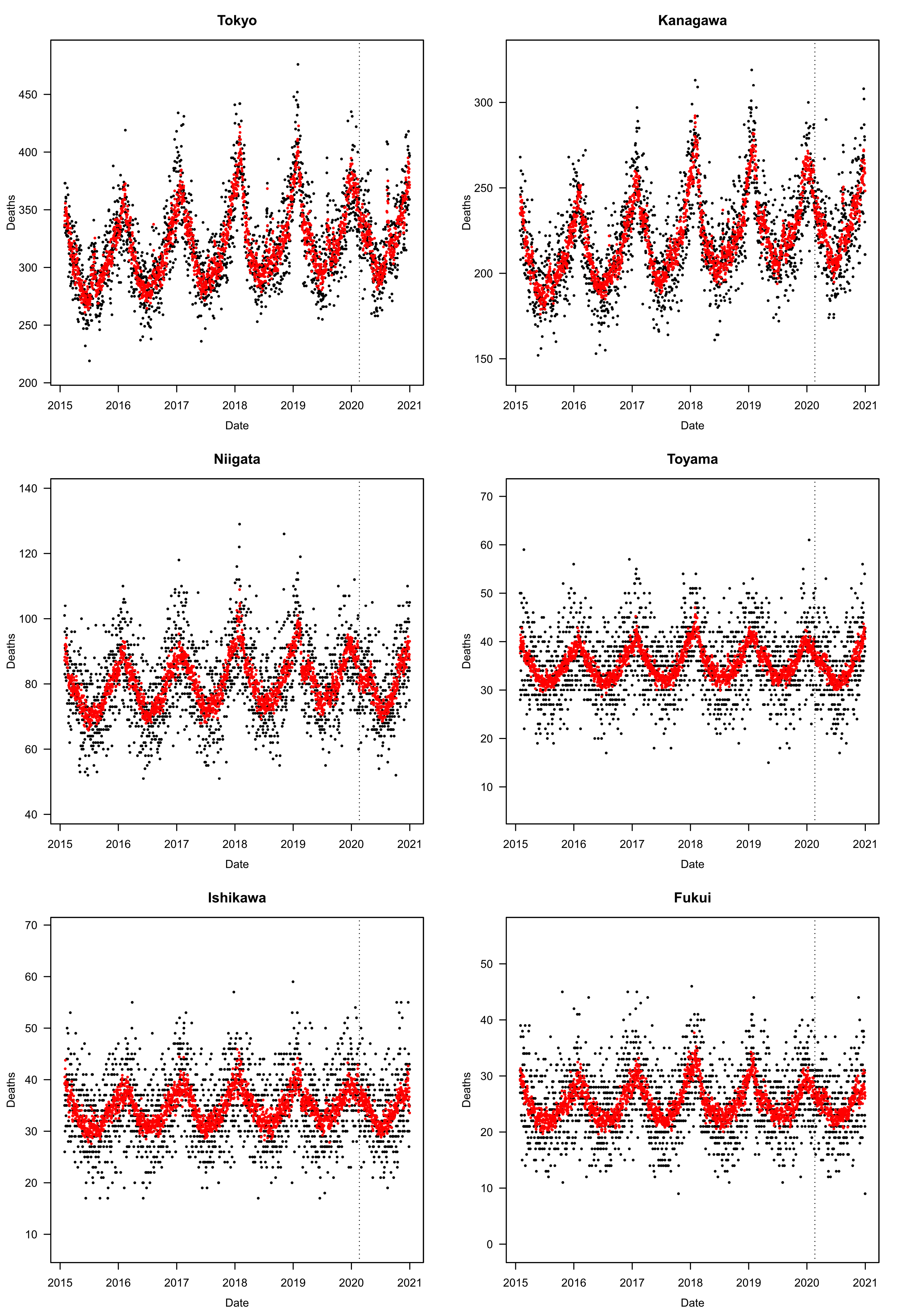


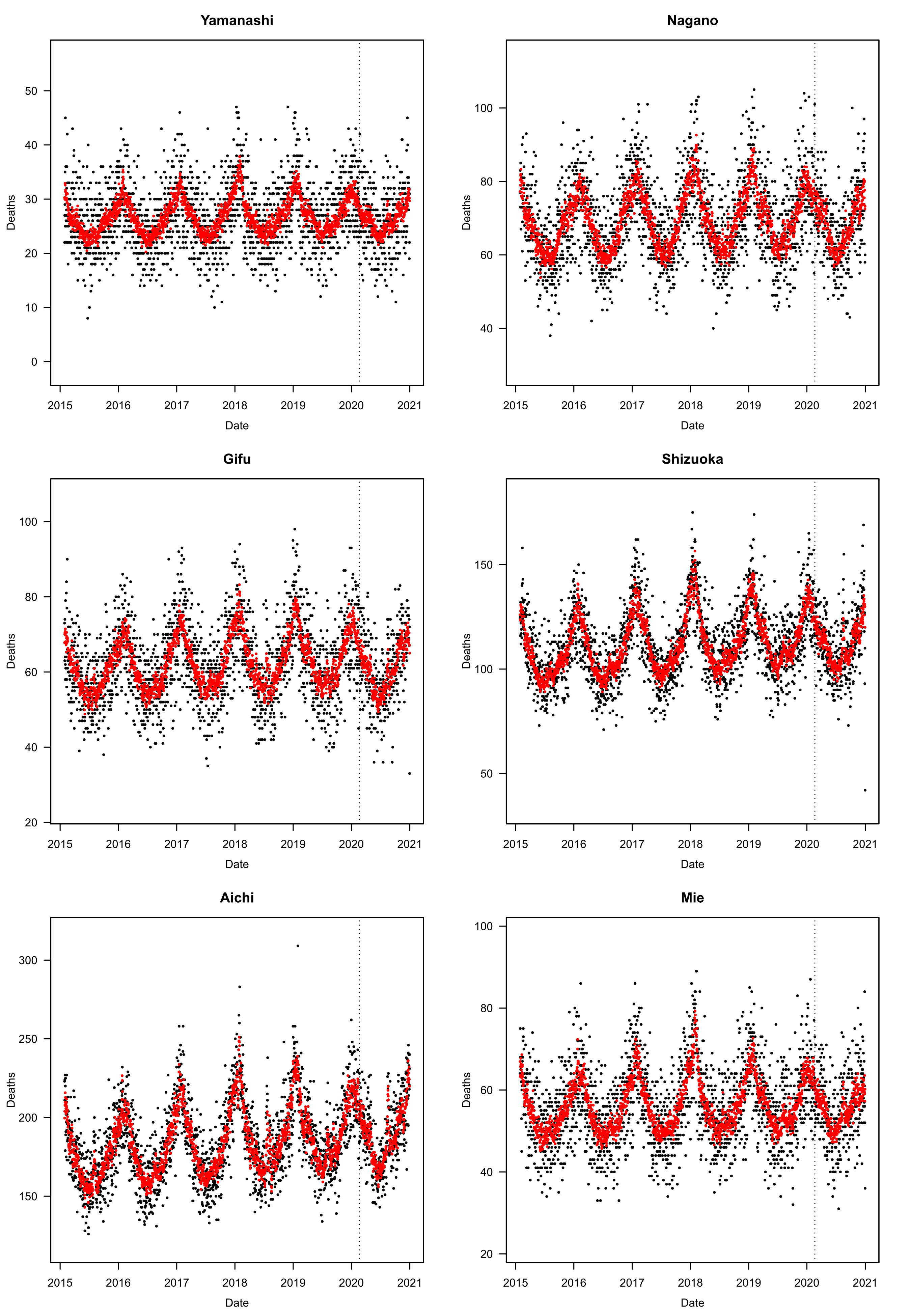


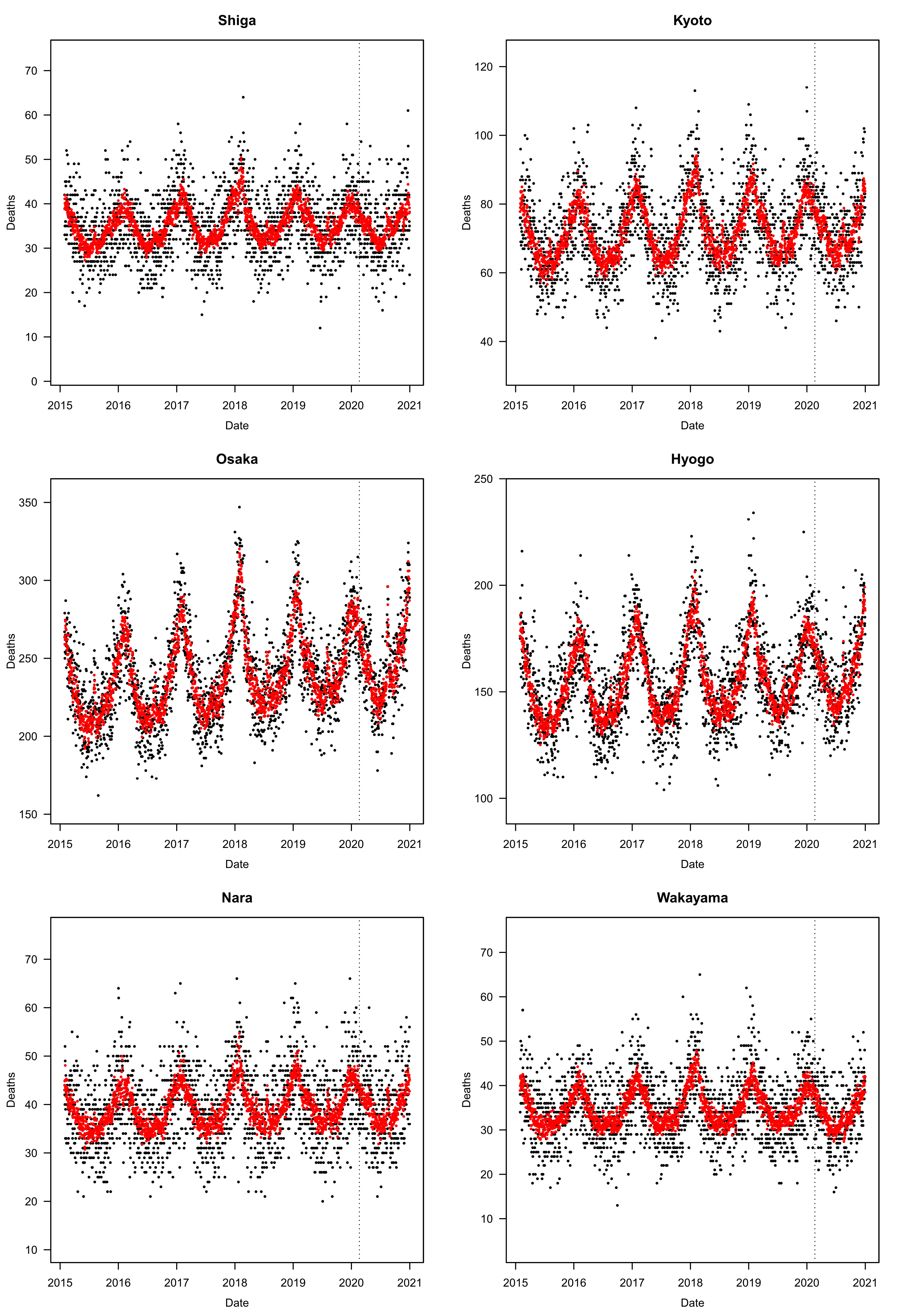


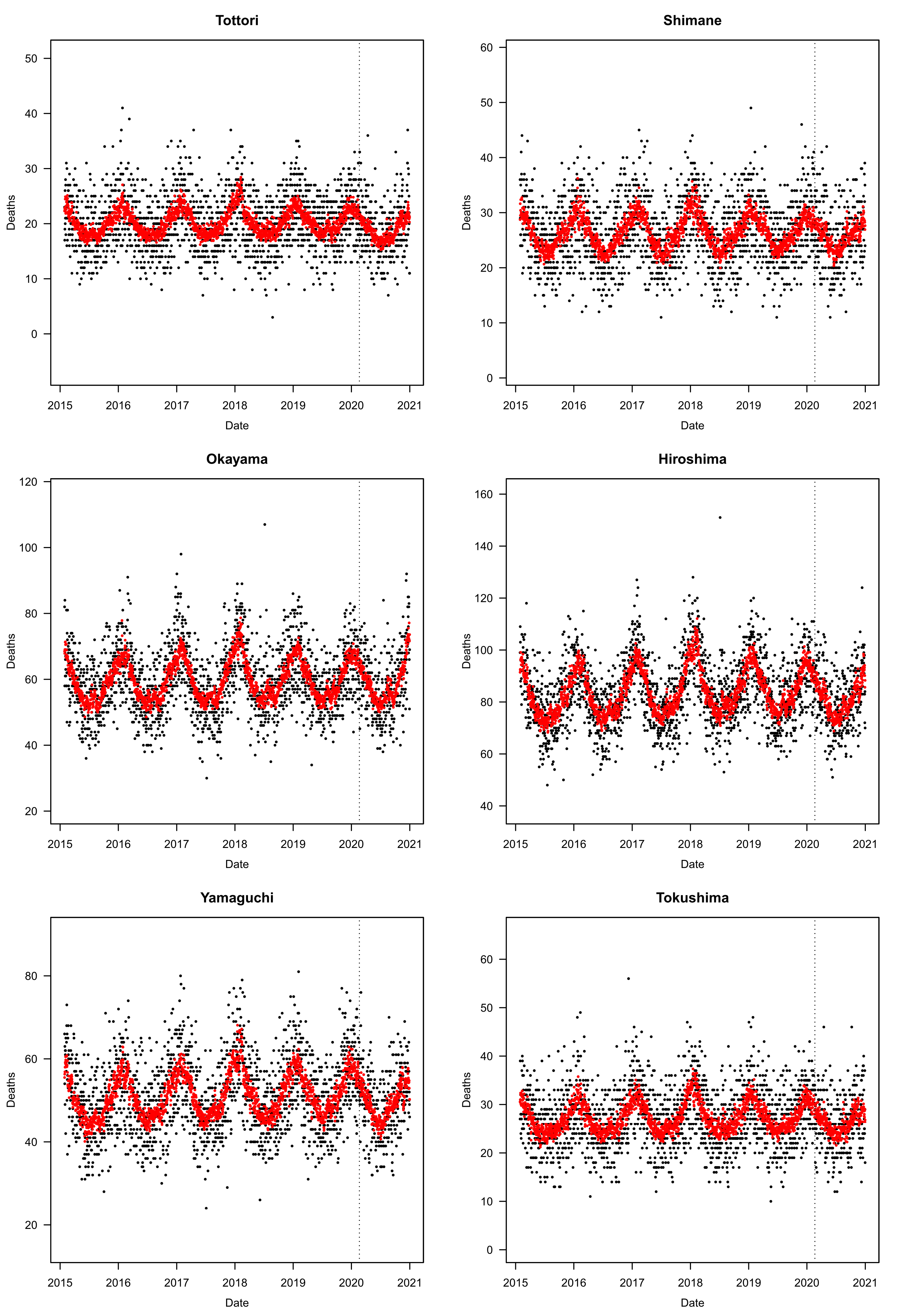


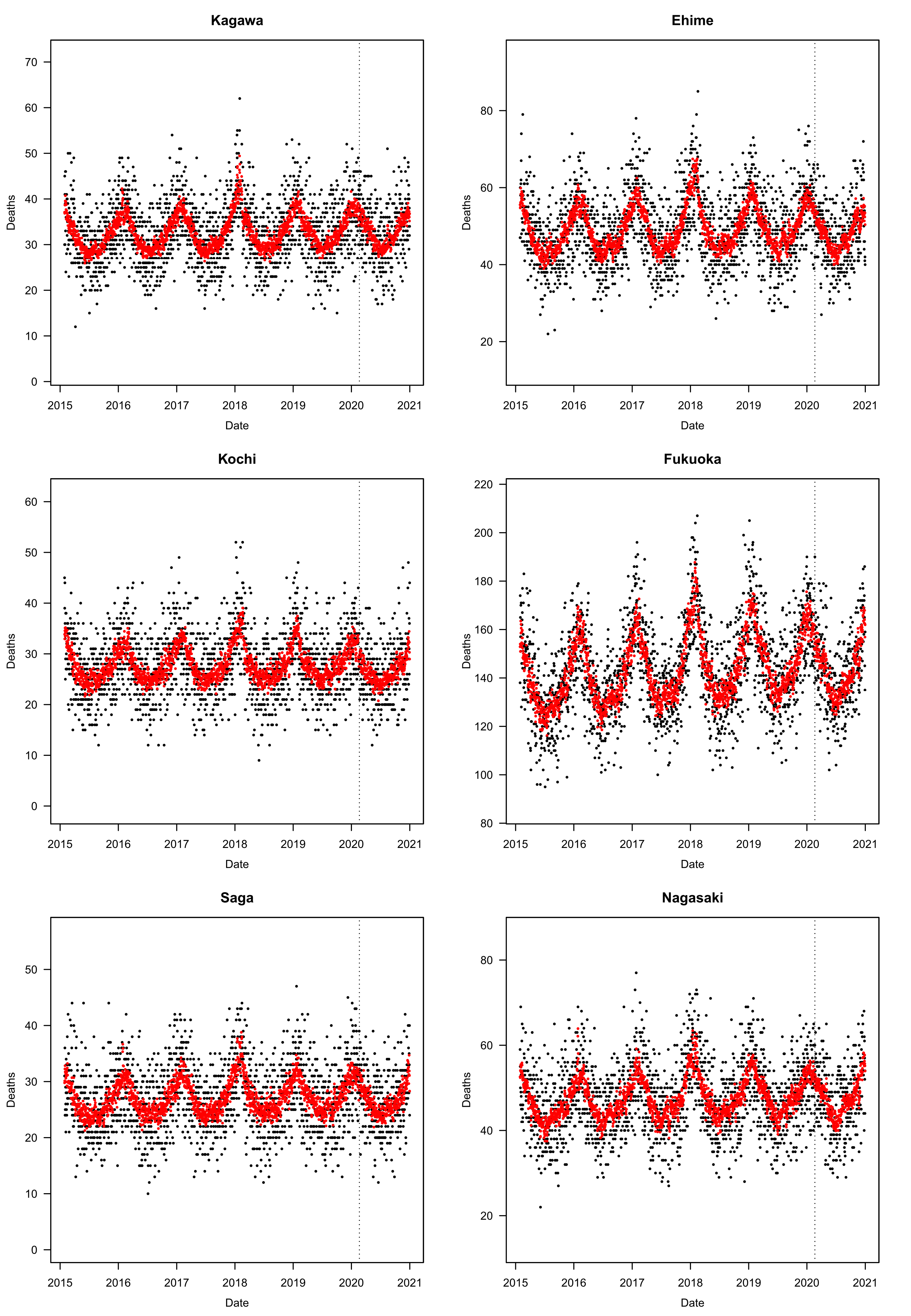


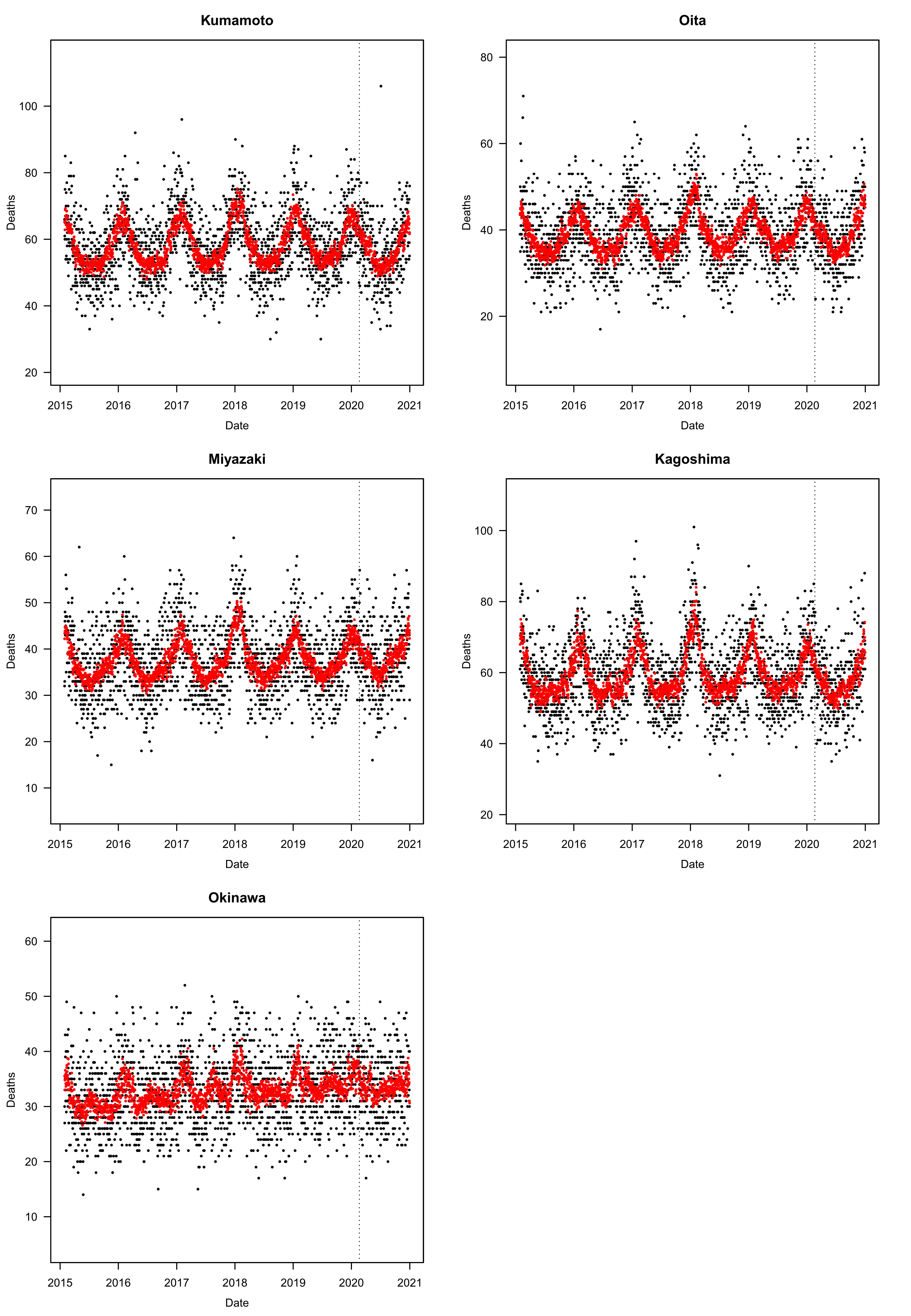


**Figure S3.** Diagnostics of models: autocorrelation function of the residuals in the 47 Japanese prefectures.


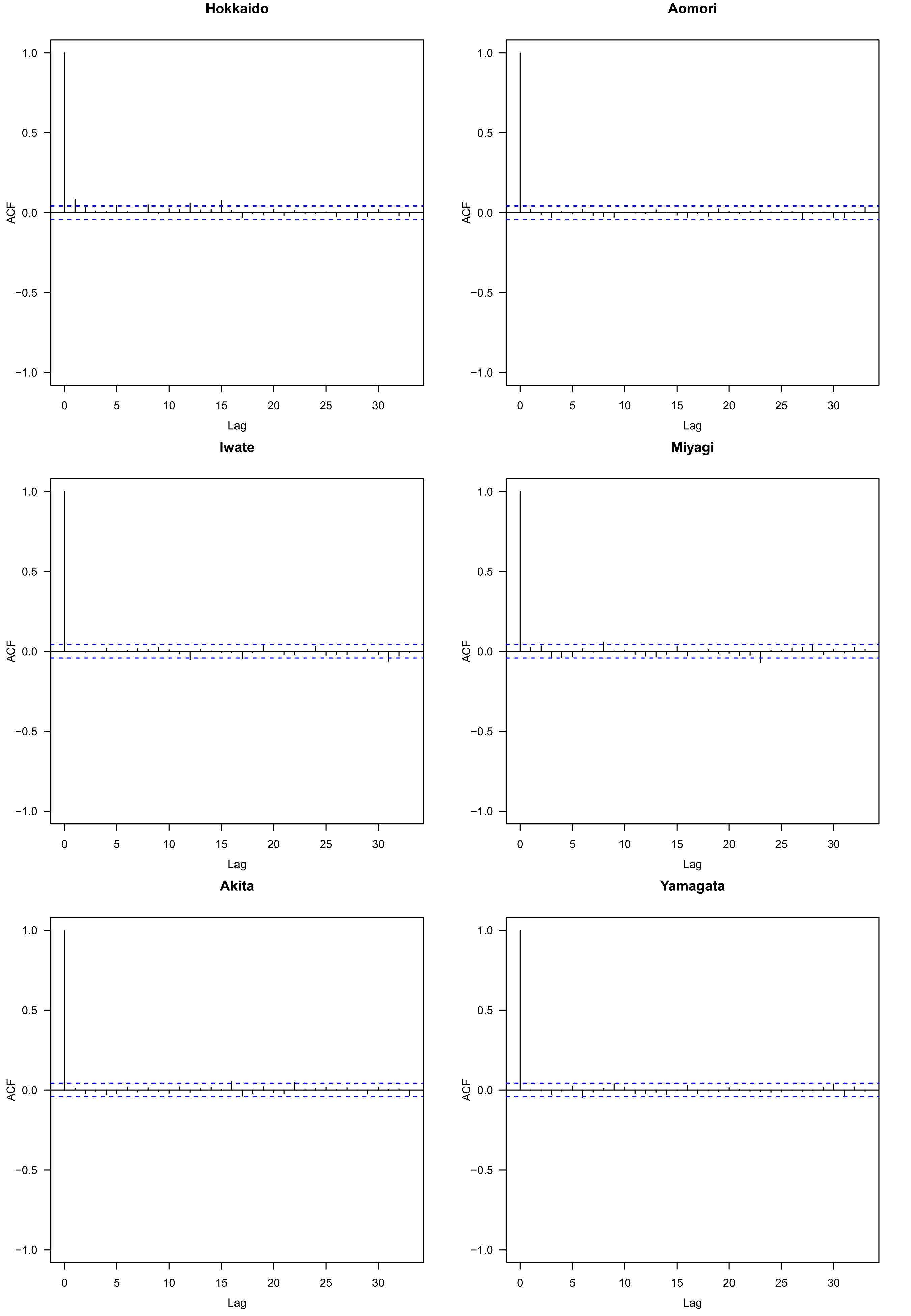


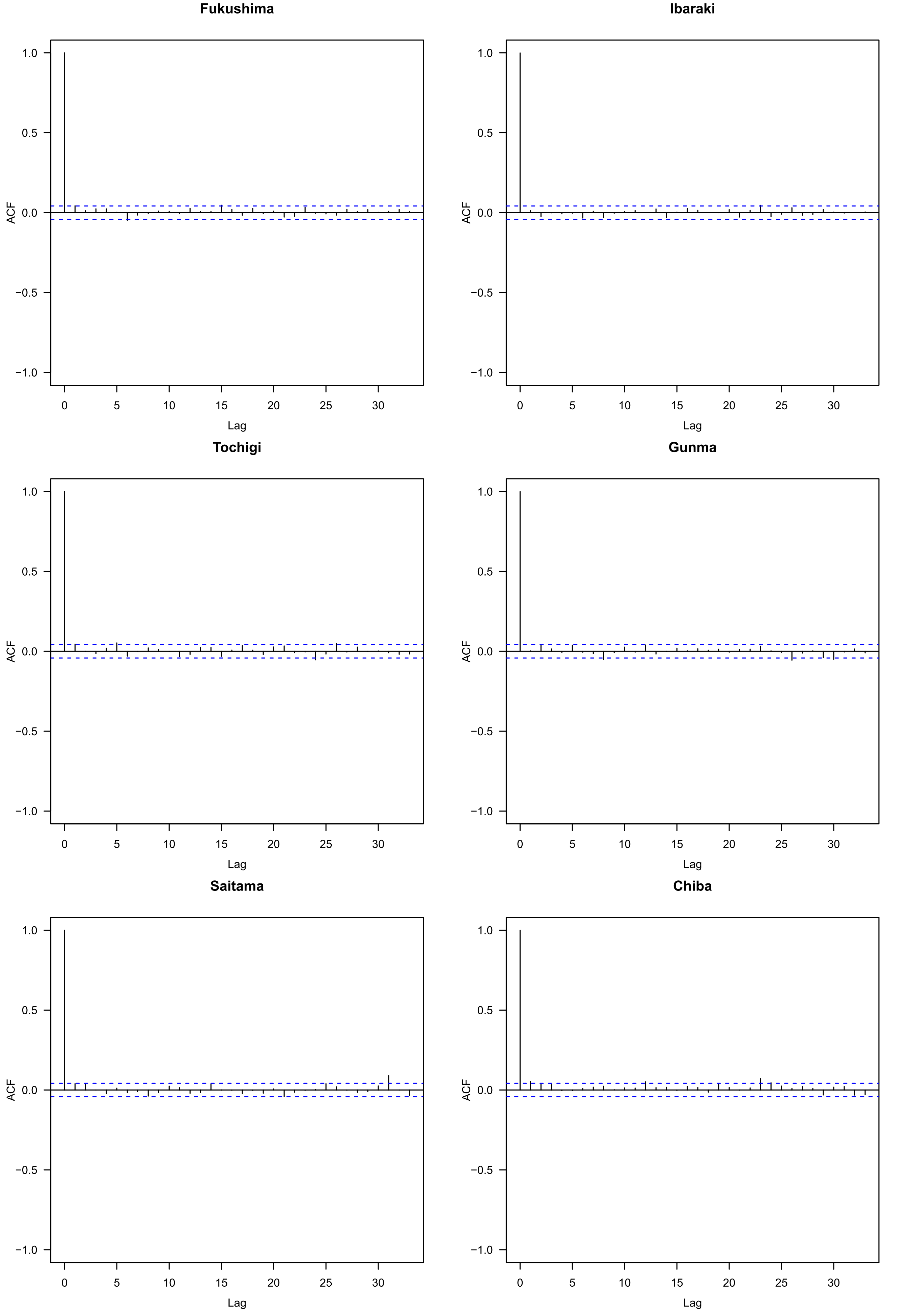


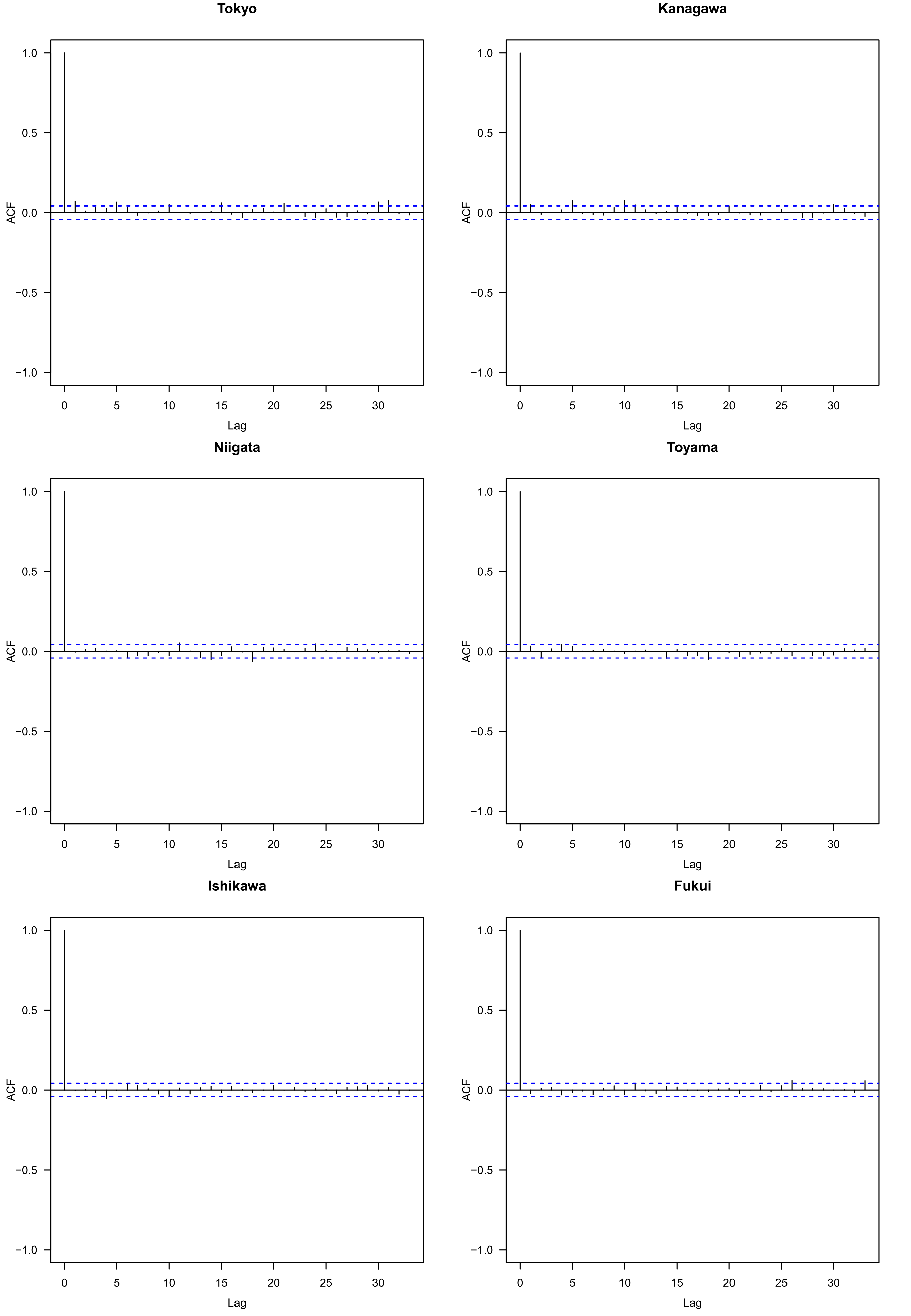


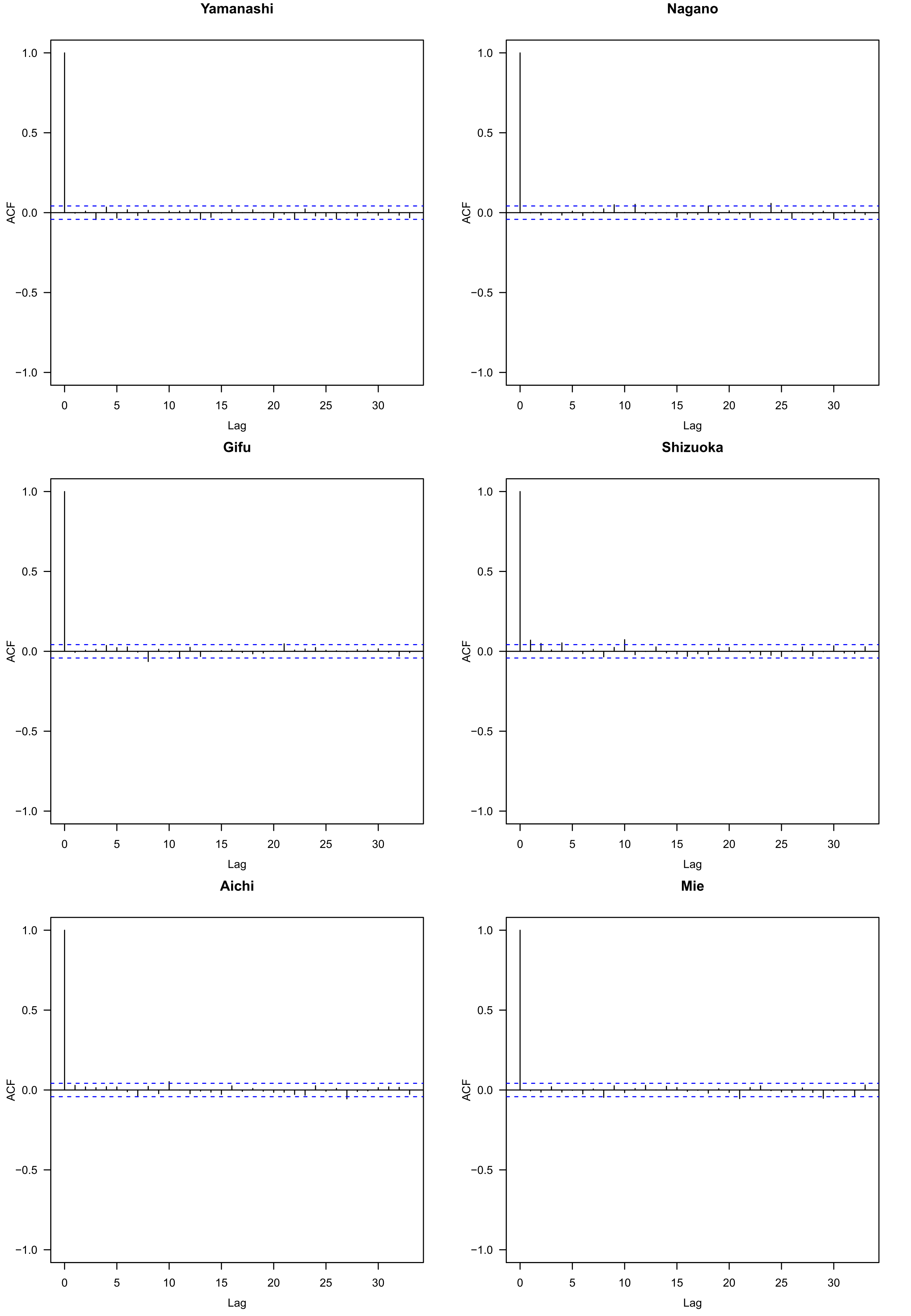


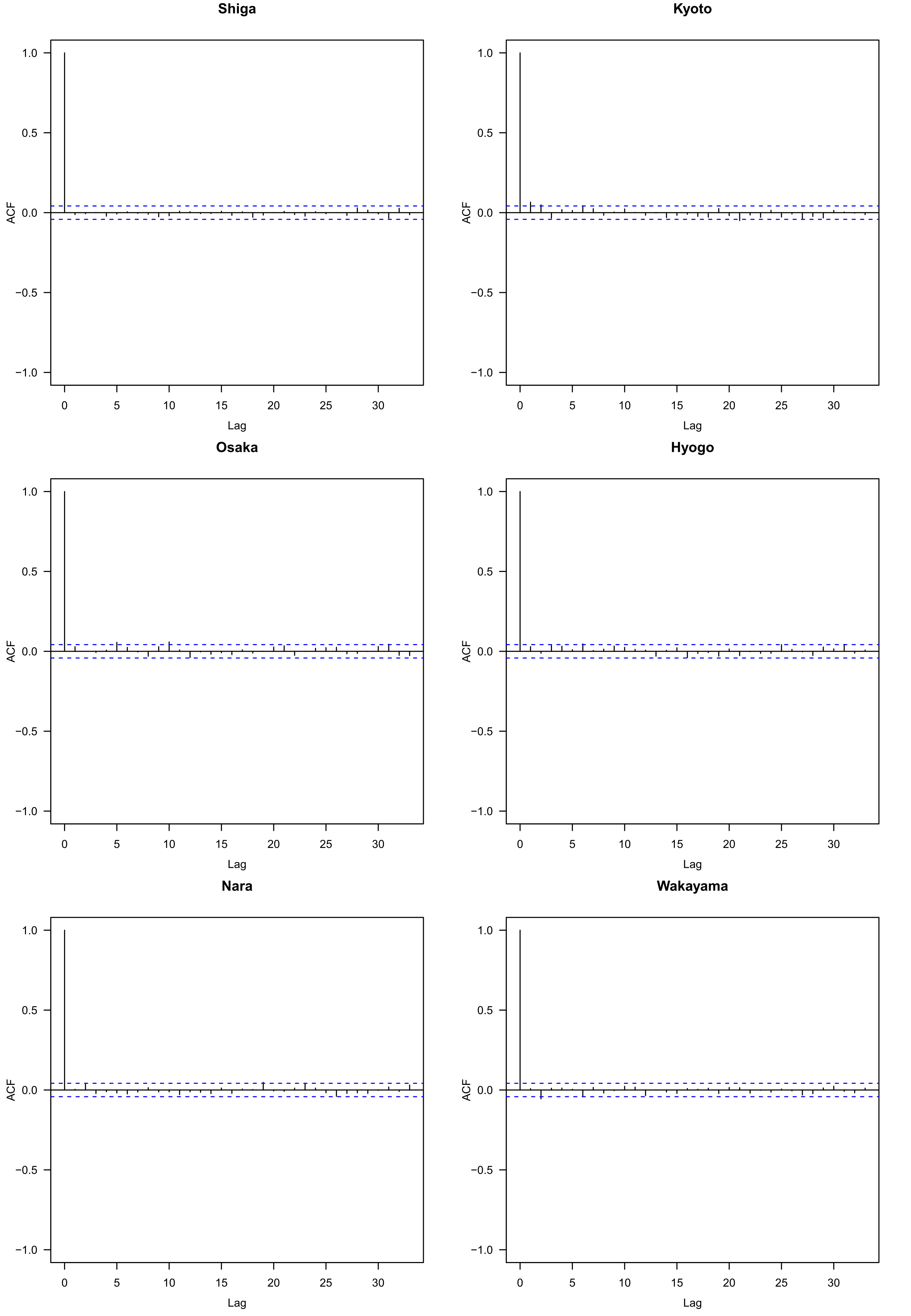


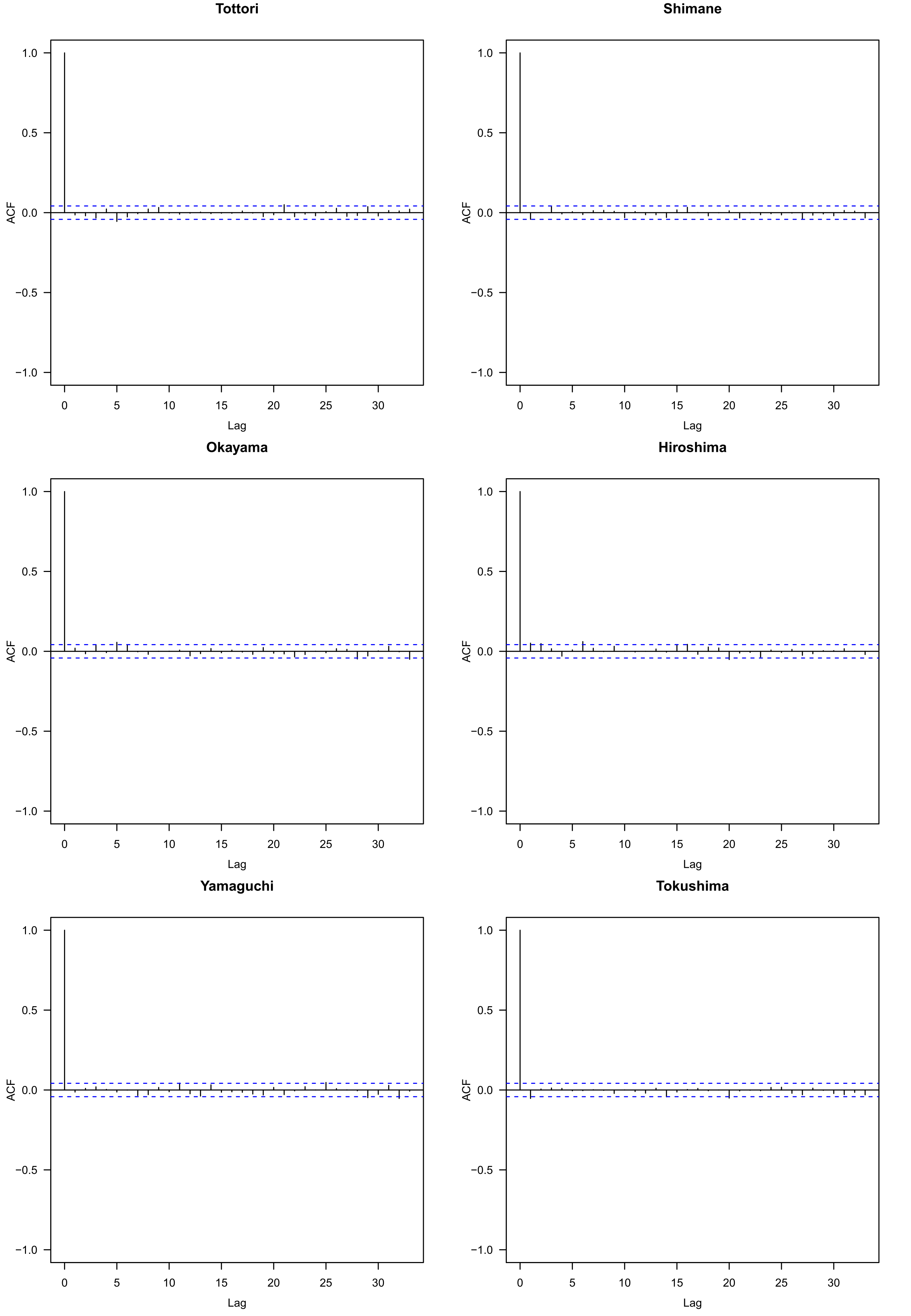


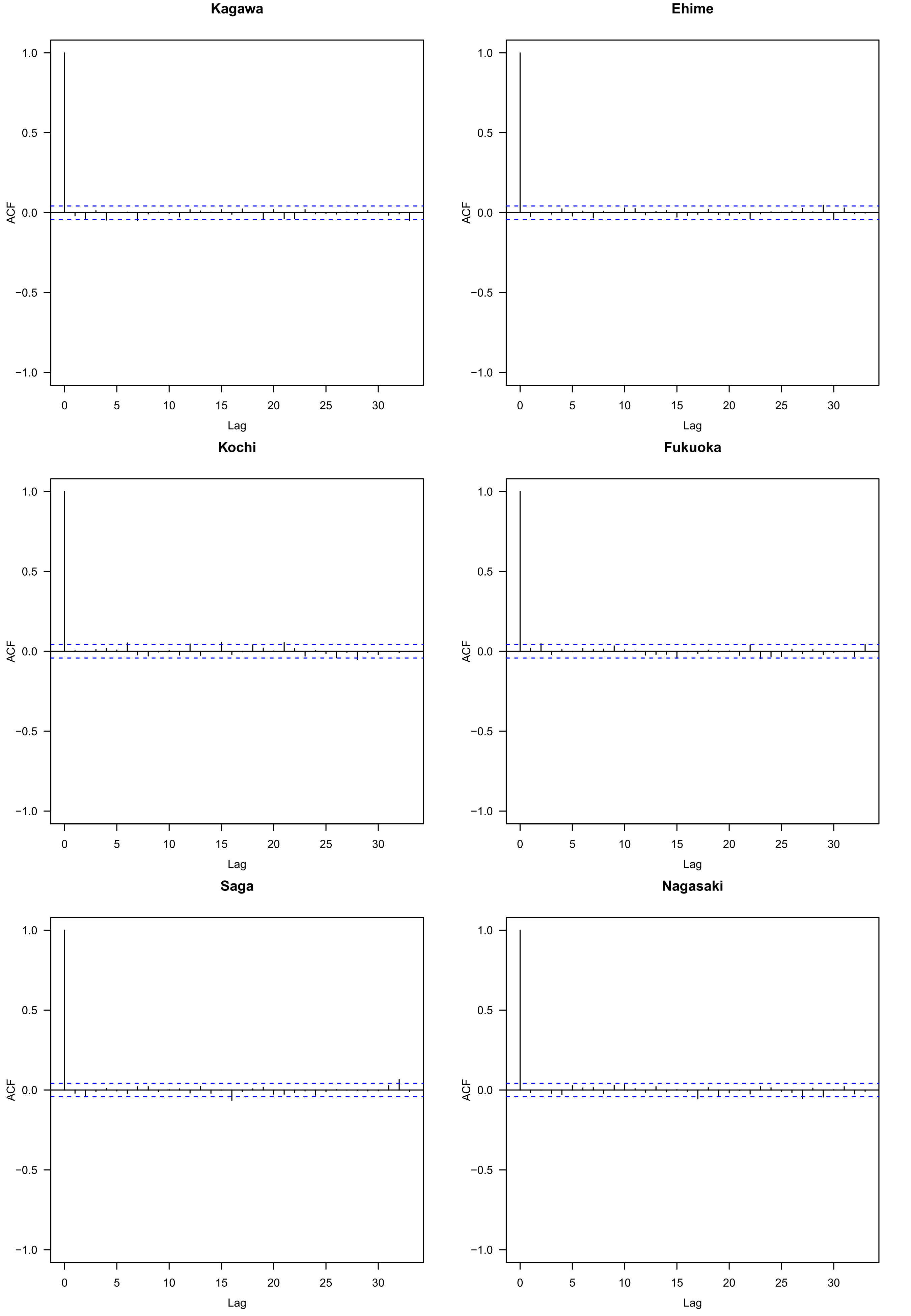


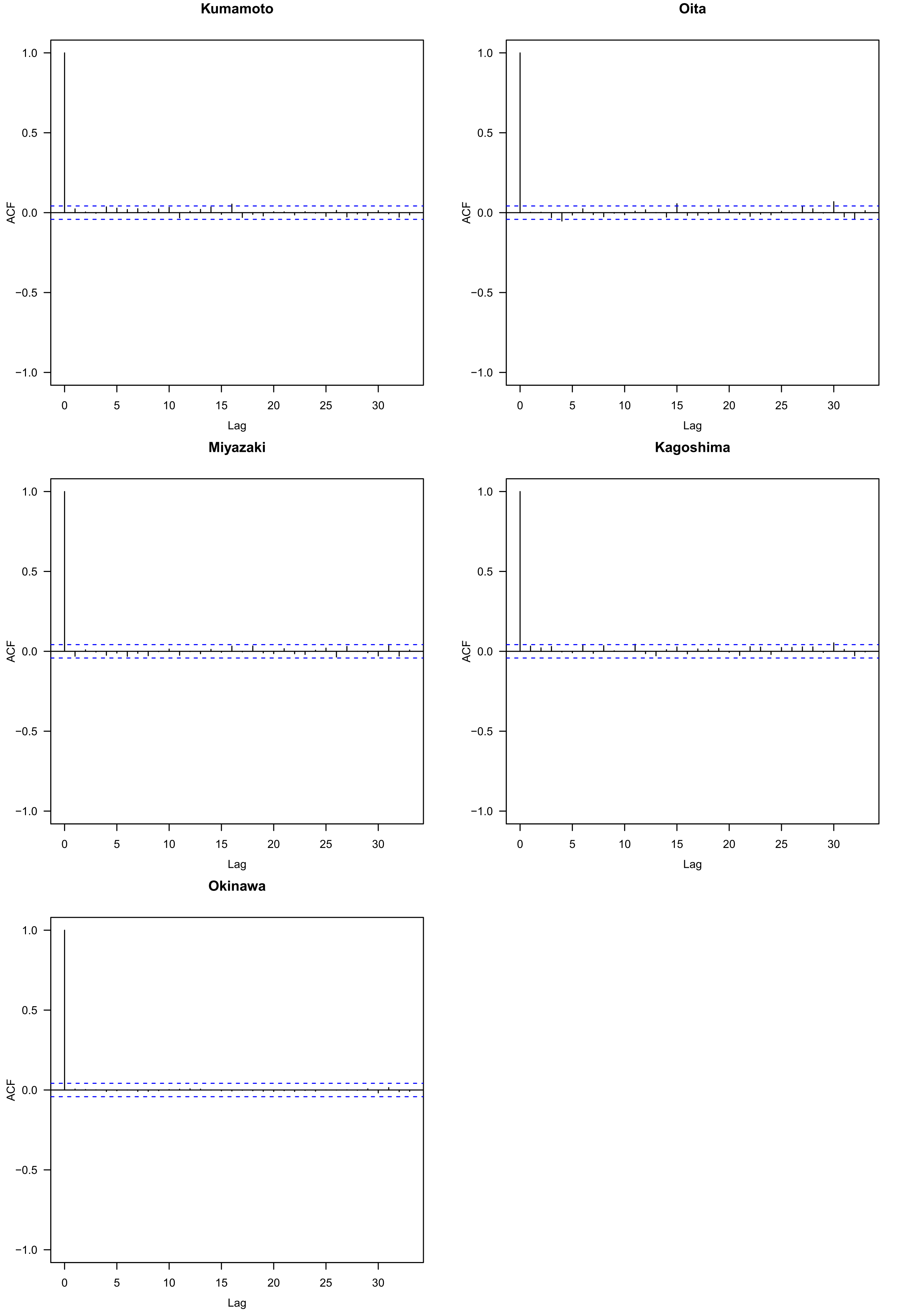


**Figure S4.** Diagnostics of models: partial autocorrelation function of the residuals in the 47 Japanese prefectures.


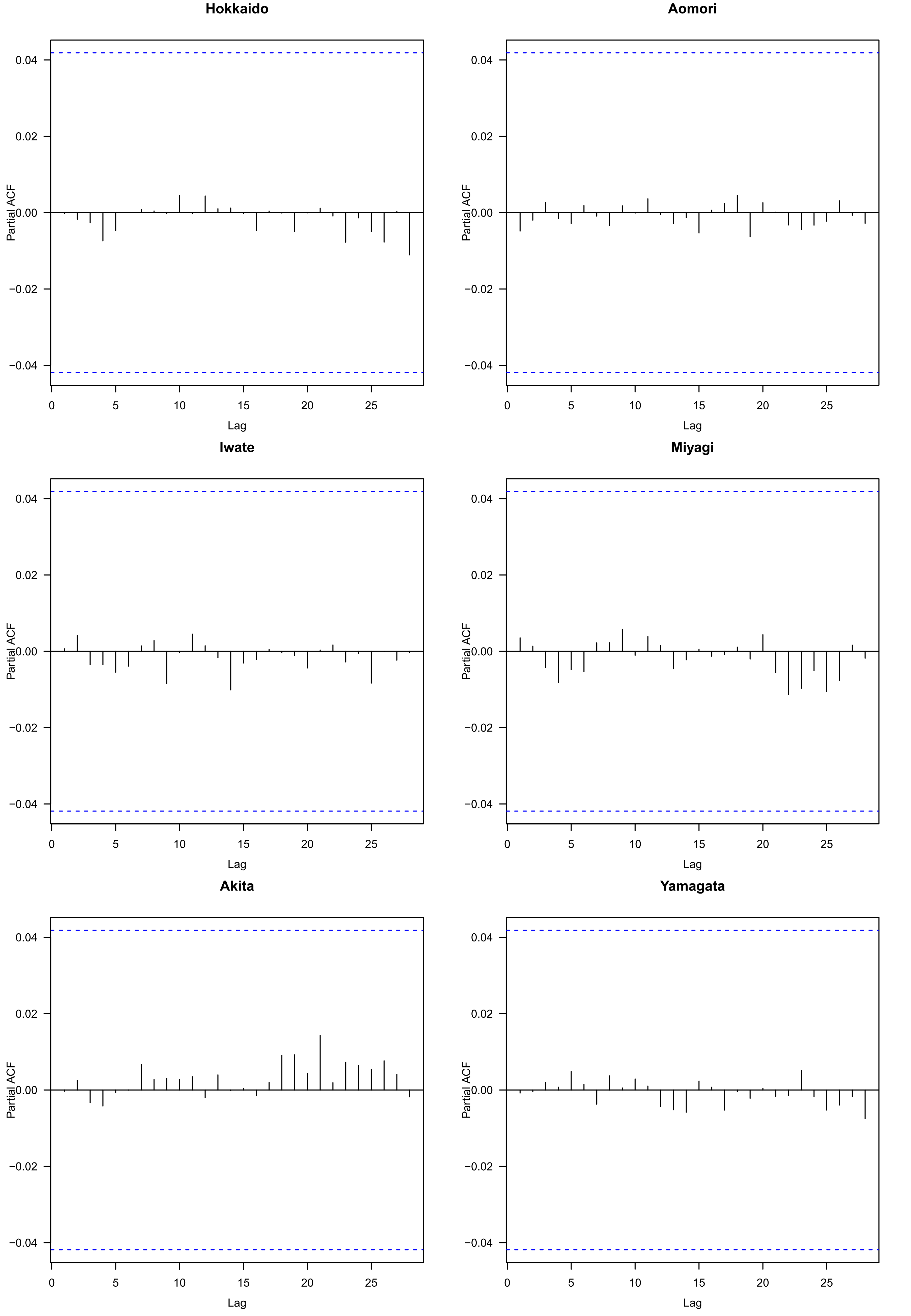


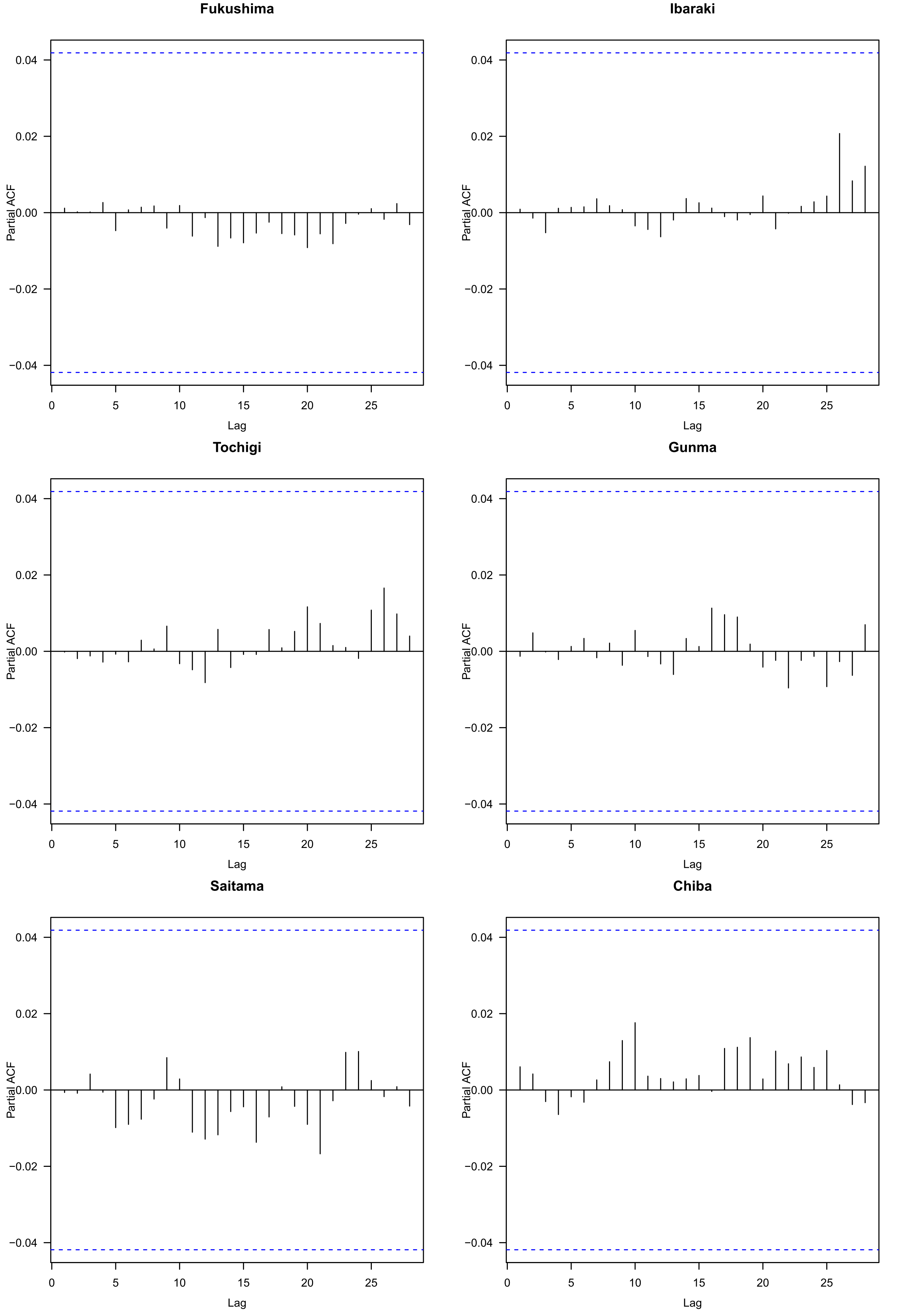


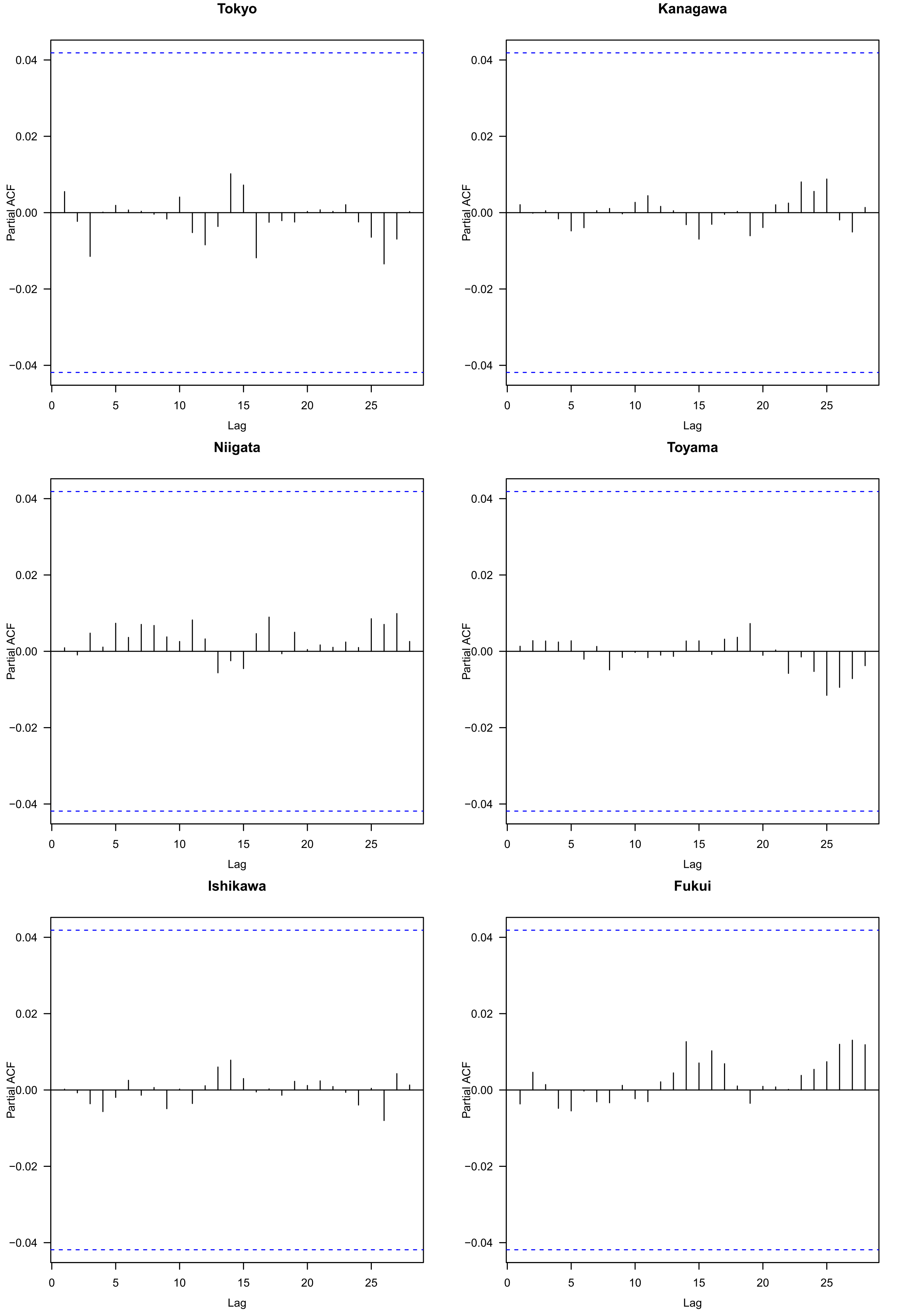


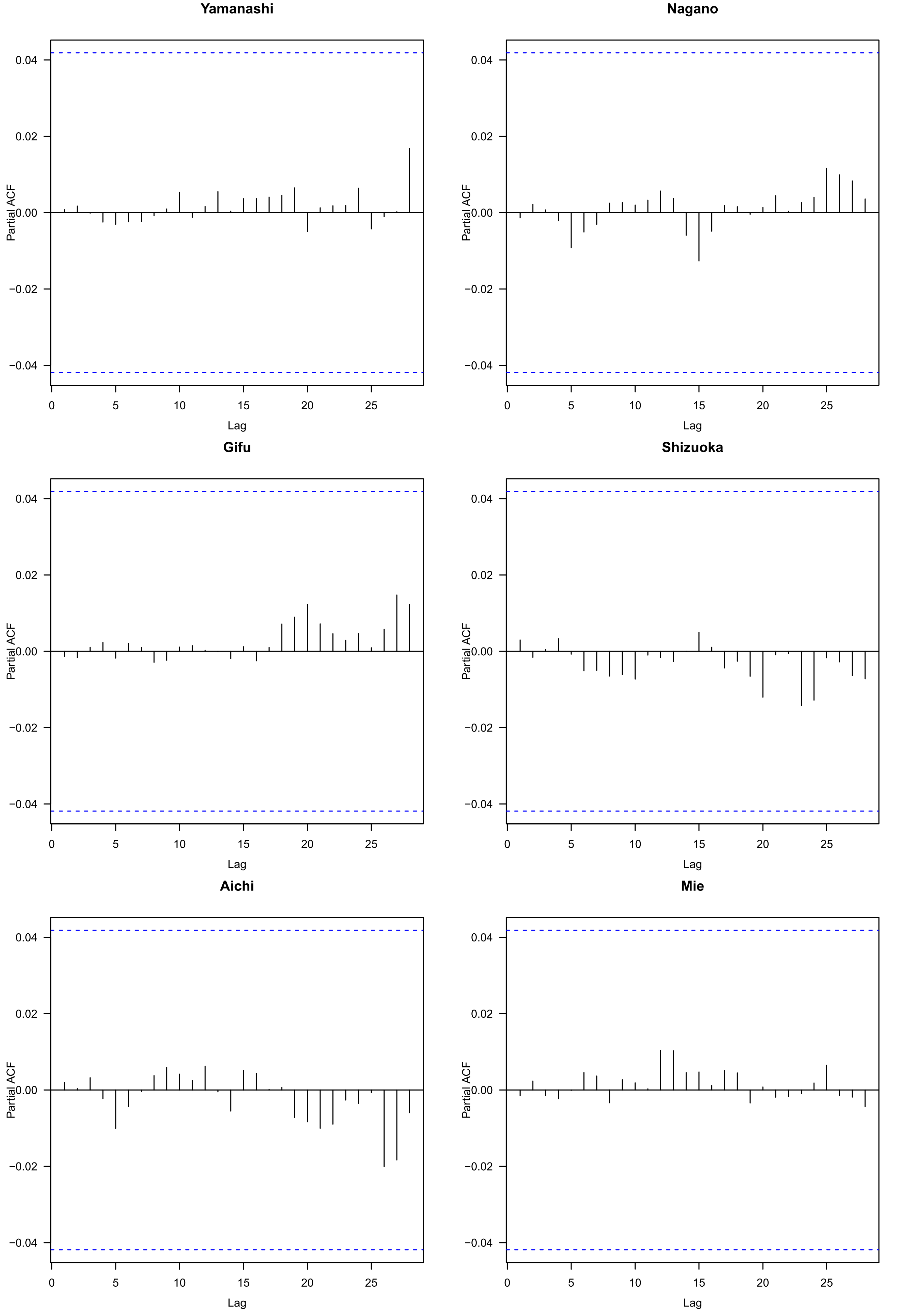


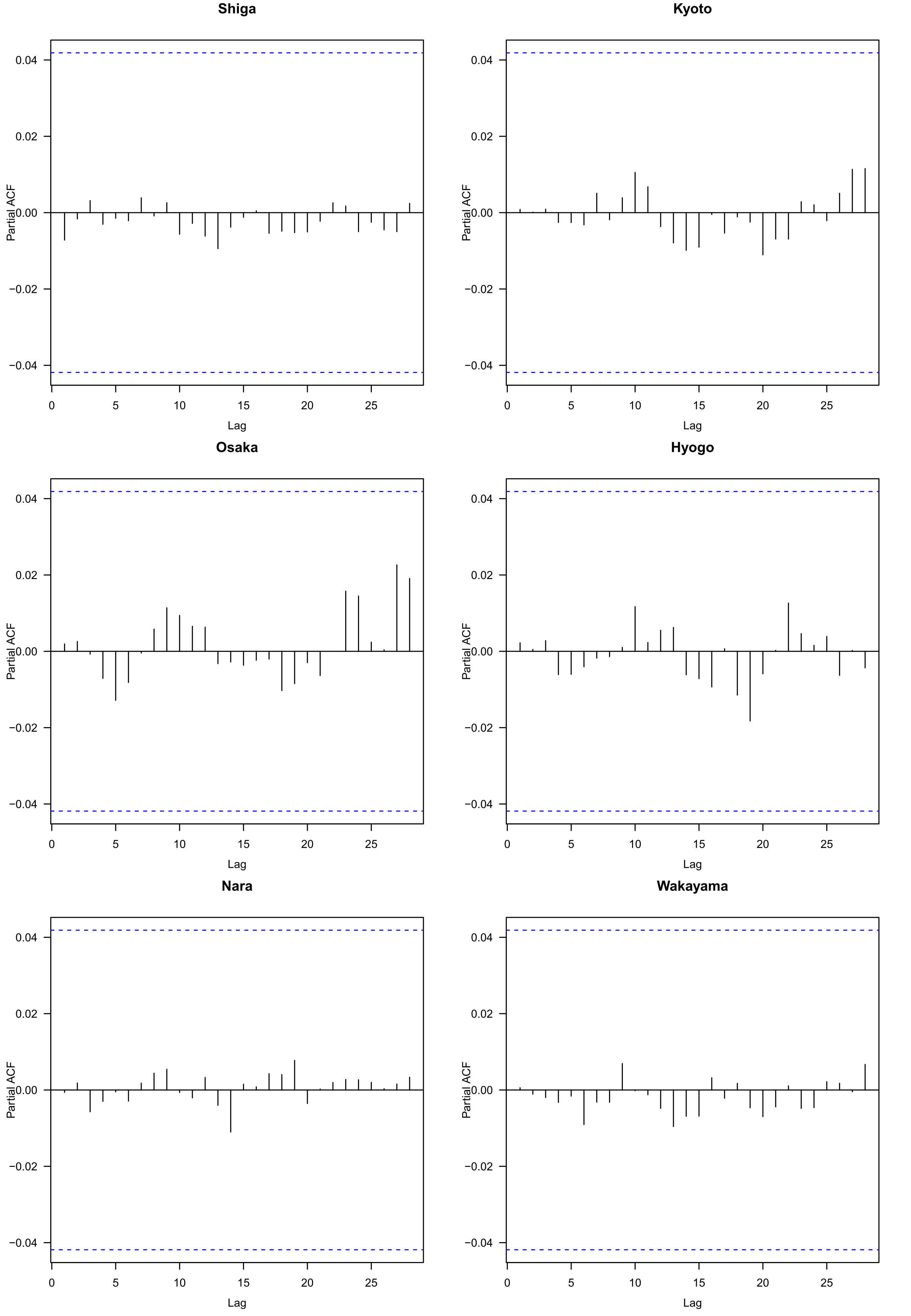


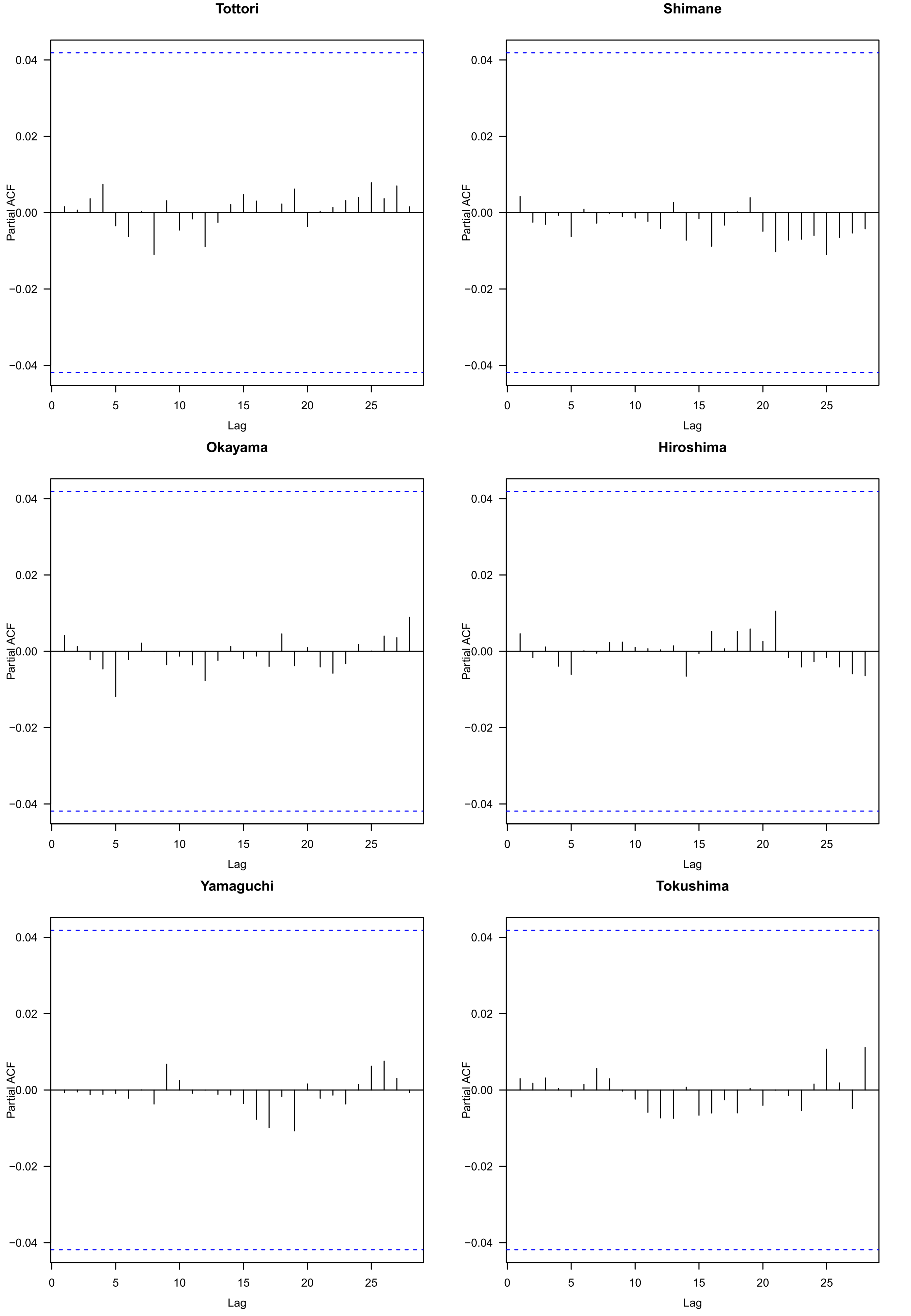


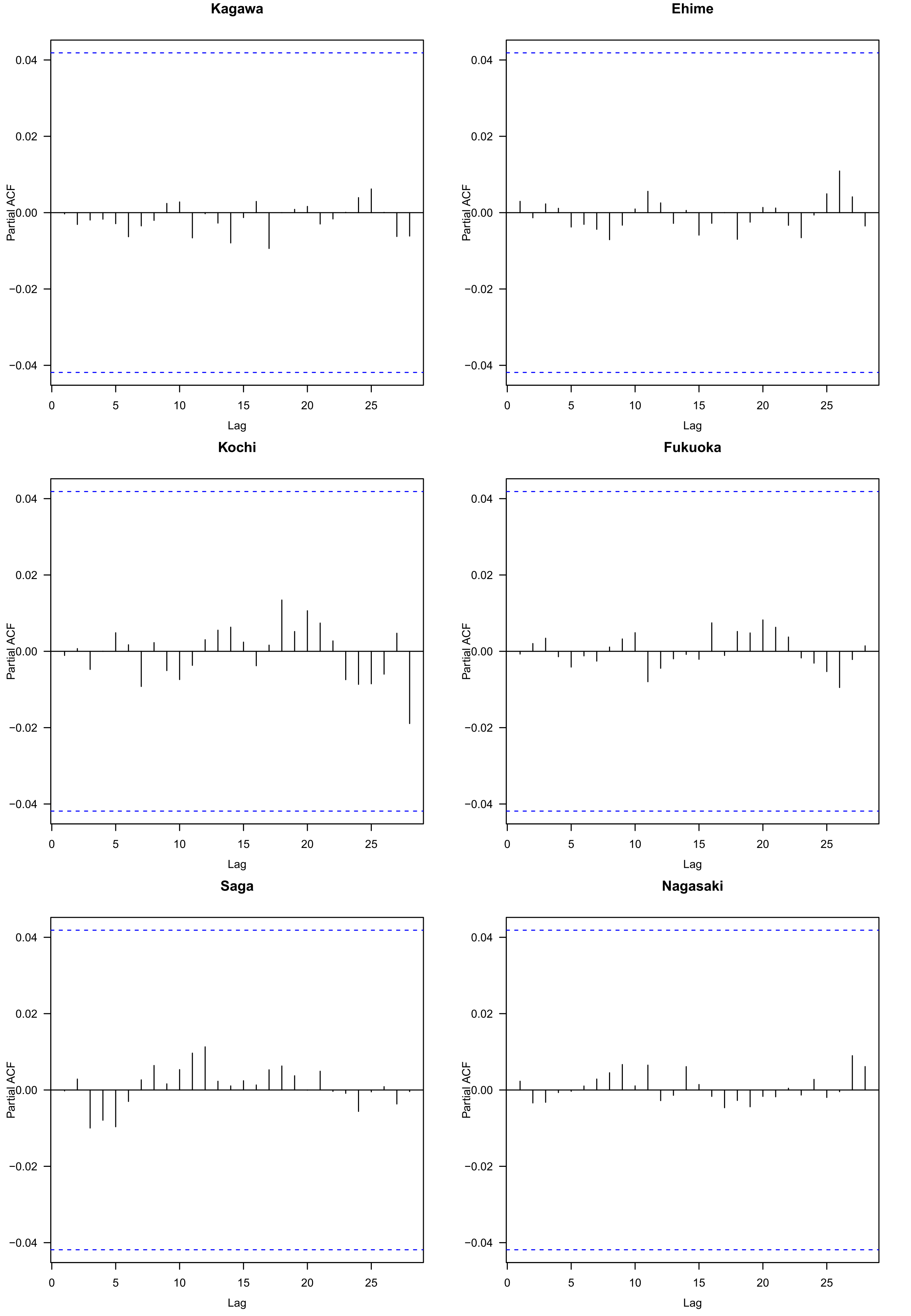


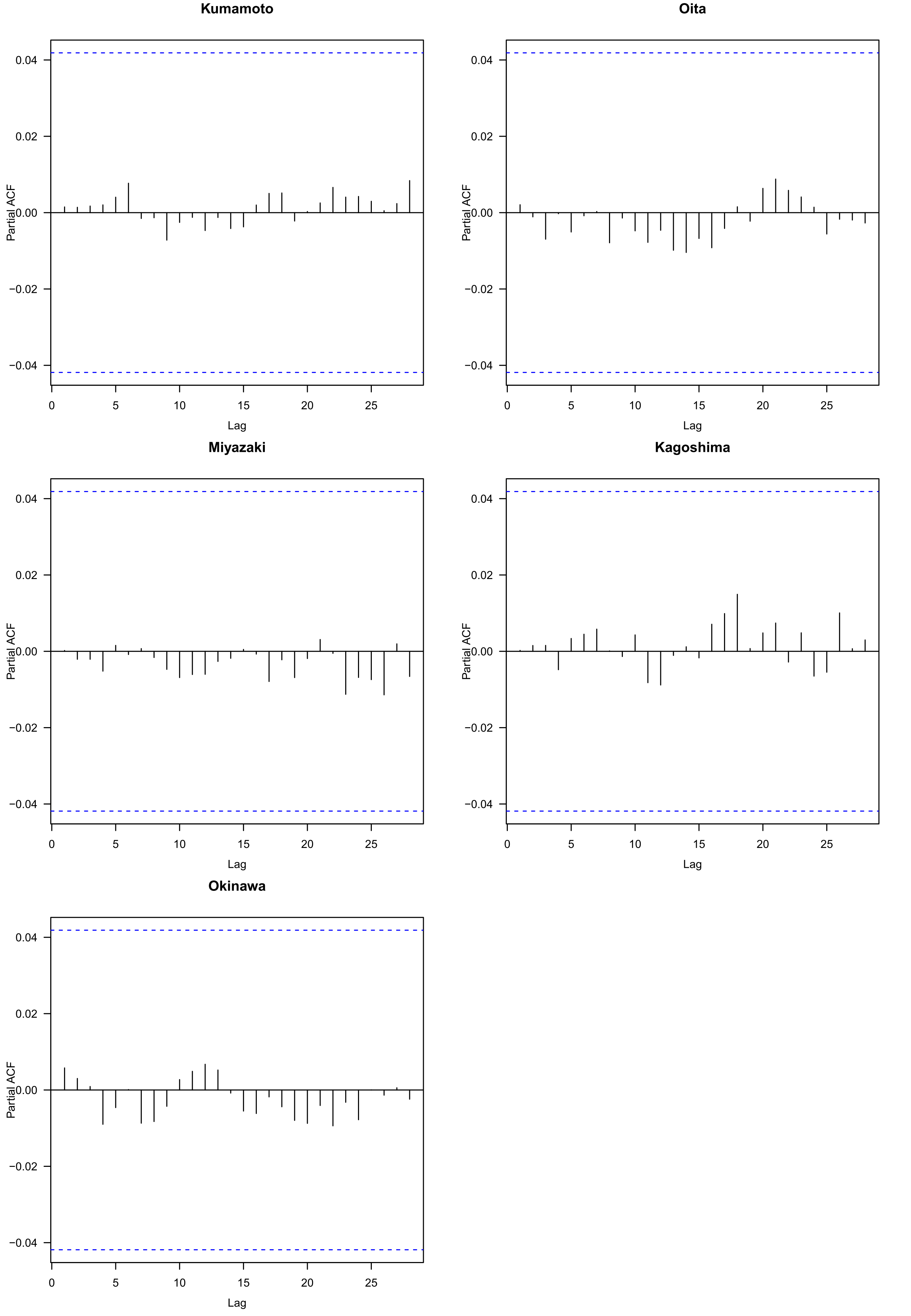


**Figure S5.** Trends in estimated excess risk (relative risk) during the period 14 January–31 December 2020 in Japan by prefectures and pooled estimates (band for pooled estimates corresponds to 95% empirical confidence intervals).


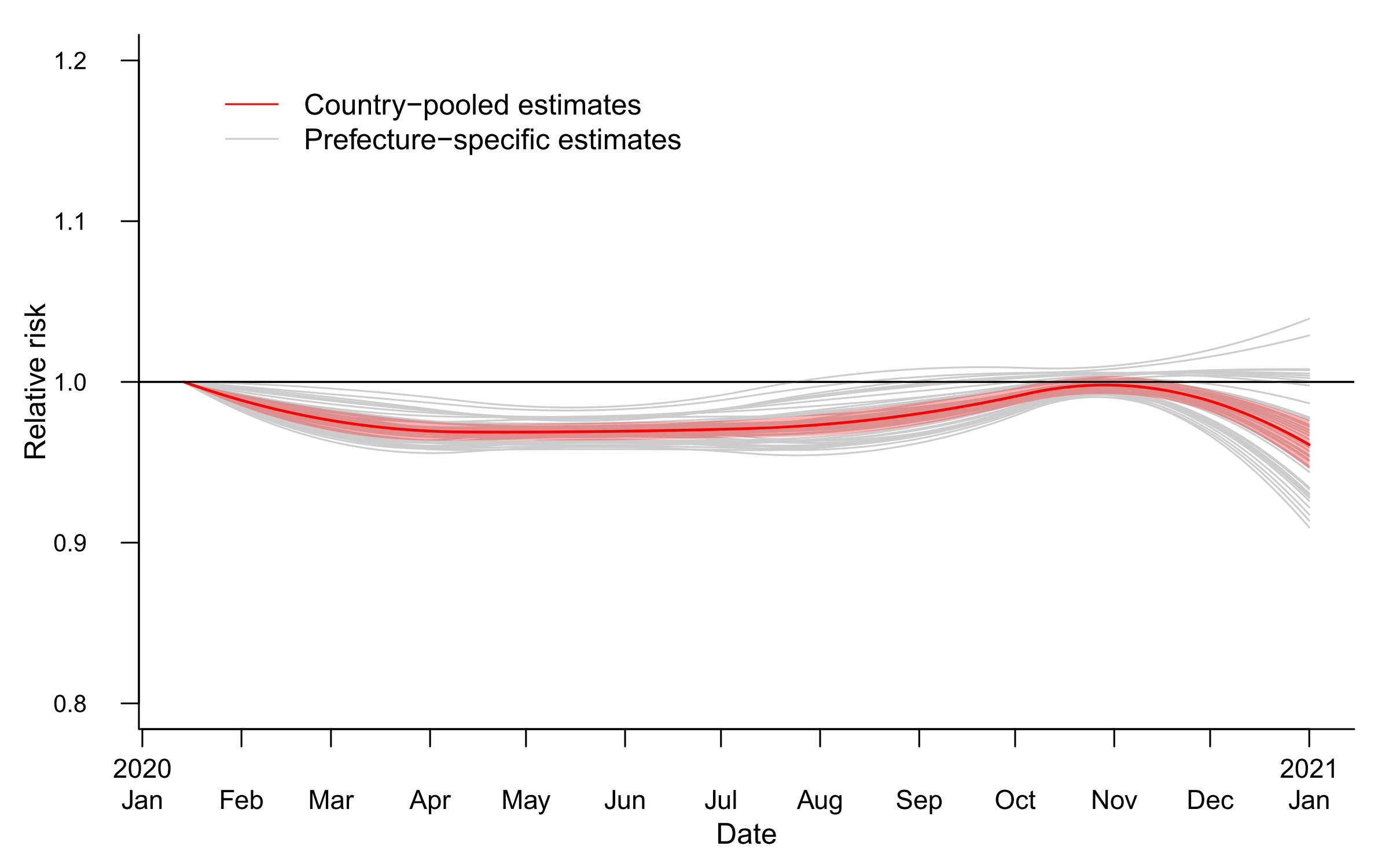


**Figure S6.** Sensitivity analysis (A) without adjusting for influenza activity and ambient temperature; (B) with adjusting for influenza activity; (C) with adjusting for ambient temperature; (D) with 4 knots in the interrupted spline component; (E) with 5 knots in the interrupted spline component; (F) with 6 knots in the interrupted spline component: Trends in estimated excess risk (relative risk) during the period 14 January–31 December 2020 in Japan by sex and age groups, compared with the total (band corresponds to 95% empirical confidence intervals).


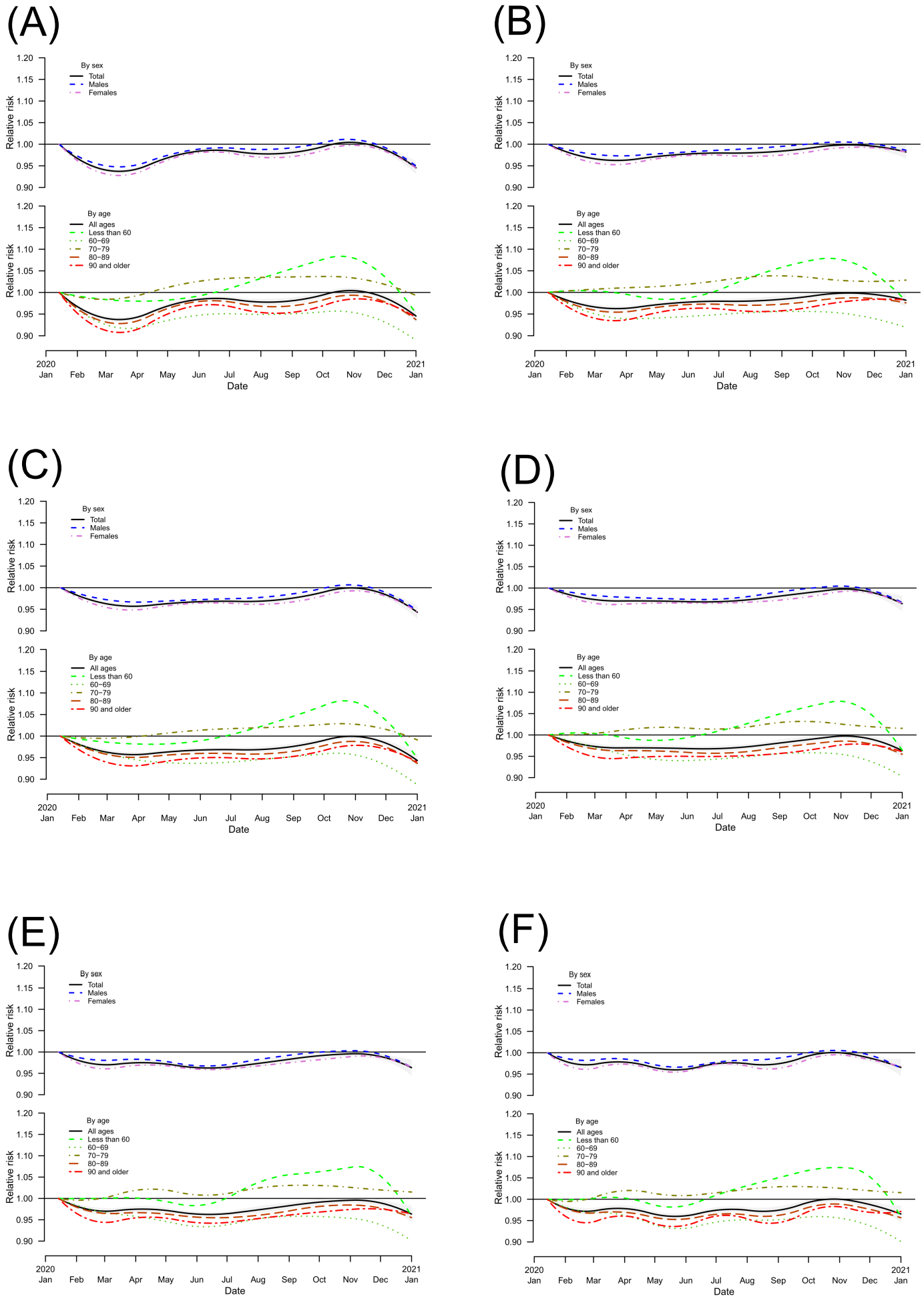


**Table S1.** Descriptive statistics before and during COVID-19 pandemic by 47 prefectures in Japan.

| Prefecture | Population sizes  in 2019 | Before COVID-19 pandemic (1 January 2015–12 February 2020) | |  | During COVID-19 pandemic (13 February–31 December 2020) | |
| --- | --- | --- | --- | --- | --- | --- |
|  |  | Total deaths | Daily mean deaths  per 100,000 population |  | Total deaths | Daily mean deaths  per 100,000 population |
| Hokkaido | 5,211,000 | 323,335 | 3.32 |  | 56,790 | 3.37 |
| Aomori | 1,240,000 | 90,790 | 3.92 |  | 15,581 | 3.89 |
| Iwate | 1,219,000 | 88,322 | 3.88 |  | 14,846 | 3.77 |
| Miyagi | 2,283,000 | 123,766 | 2.90 |  | 21,396 | 2.90 |
| Akita | 963,000 | 78,709 | 4.37 |  | 13,404 | 4.31 |
| Yamagata | 1,070,000 | 78,518 | 3.93 |  | 13,387 | 3.87 |
| Fukushima | 1,831,000 | 126,286 | 3.69 |  | 21,167 | 3.58 |
| Ibaraki | 2,810,000 | 165,608 | 3.15 |  | 28,446 | 3.13 |
| Tochigi | 1,906,000 | 110,786 | 3.11 |  | 18,811 | 3.06 |
| Gunma | 1,886,000 | 115,572 | 3.28 |  | 20,218 | 3.32 |
| Saitama | 7,174,000 | 338,780 | 2.53 |  | 61,511 | 2.65 |
| Chiba | 6,141,000 | 301,562 | 2.63 |  | 53,904 | 2.72 |
| Tokyo | 13,405,000 | 598,731 | 2.39 |  | 105,503 | 2.44 |
| Kanagawa | 8,997,000 | 411,713 | 2.45 |  | 73,390 | 2.53 |
| Niigata | 2,206,000 | 150,875 | 3.66 |  | 25,746 | 3.61 |
| Toyama | 1,026,000 | 66,722 | 3.48 |  | 11,338 | 3.42 |
| Ishikawa | 1,123,000 | 64,751 | 3.09 |  | 11,053 | 3.05 |
| Fukui | 756,000 | 47,613 | 3.37 |  | 8,034 | 3.29 |
| Yamanashi | 798,000 | 50,285 | 3.37 |  | 8,448 | 3.28 |
| Nagano | 2,016,000 | 130,196 | 3.46 |  | 22,071 | 3.39 |
| Gifu | 1,940,000 | 117,050 | 3.23 |  | 19,661 | 3.14 |
| Shizuoka | 3,557,000 | 210,066 | 3.16 |  | 36,166 | 3.15 |
| Aichi | 7,316,000 | 345,010 | 2.52 |  | 61,214 | 2.59 |
| Mie | 1,736,000 | 105,311 | 3.25 |  | 17,939 | 3.20 |
| Shiga | 1,385,000 | 66,317 | 2.56 |  | 11,295 | 2.52 |
| Kyoto | 2,527,000 | 135,206 | 2.86 |  | 23,267 | 2.85 |
| Osaka | 8,623,000 | 447,471 | 2.78 |  | 79,626 | 2.86 |
| Hyogo | 5,369,000 | 290,753 | 2.90 |  | 51,026 | 2.94 |
| Nara | 1,319,000 | 73,830 | 2.99 |  | 12,692 | 2.98 |
| Wakayama | 918,000 | 65,625 | 3.82 |  | 10,869 | 3.67 |
| Tottori | 551,000 | 38,034 | 3.69 |  | 6,133 | 3.45 |
| Shimane | 665,000 | 49,591 | 3.99 |  | 8,295 | 3.86 |
| Okayama | 1,866,000 | 111,927 | 3.21 |  | 18,952 | 3.14 |
| Hiroshima | 2,761,000 | 157,336 | 3.05 |  | 26,255 | 2.94 |
| Yamaguchi | 1,340,000 | 95,779 | 3.82 |  | 15,998 | 3.70 |
| Tokushima | 723,000 | 51,428 | 3.81 |  | 8,525 | 3.65 |
| Kagawa | 945,000 | 61,361 | 3.47 |  | 10,587 | 3.47 |
| Ehime | 1,328,000 | 92,522 | 3.73 |  | 15,507 | 3.62 |
| Kochi | 693,000 | 52,484 | 4.05 |  | 8,578 | 3.83 |
| Fukuoka | 5,039,000 | 268,484 | 2.85 |  | 46,226 | 2.84 |
| Saga | 808,000 | 50,865 | 3.37 |  | 8,603 | 3.30 |
| Nagasaki | 1,318,000 | 89,074 | 3.62 |  | 15,402 | 3.62 |
| Kumamoto | 1,731,000 | 109,588 | 3.39 |  | 18,324 | 3.28 |
| Oita | 1,123,000 | 73,732 | 3.51 |  | 12,488 | 3.44 |
| Miyazaki | 1,065,000 | 70,593 | 3.55 |  | 12,278 | 3.57 |
| Kagoshima | 1,589,000 | 111,633 | 3.76 |  | 18,602 | 3.62 |
| Okinawa | 1,434,000 | 61,309 | 2.29 |  | 10,857 | 2.34 |

**Table S2.** Number of observed and estimated excess deaths (95% empirical confidence interval) during the period 13 February–31 December 2020 by prefecture in Japan.

| Prefecture | Total | | |  | Males | | |  | Females | | |  | Less than 60 | | |  | 60-69 | | |  | 70-79 | | |  | 80-89 | | |  | 90 and older | | |
| --- | --- | --- | --- | --- | --- | --- | --- | --- | --- | --- | --- | --- | --- | --- | --- | --- | --- | --- | --- | --- | --- | --- | --- | --- | --- | --- | --- | --- | --- | --- | --- |
|  | Total deaths | Excess deaths | Percentage excess |  | Total deaths | Excess deaths | Percentage excess |  | Total deaths | Excess deaths | Percentage excess |  | Total deaths | Excess deaths | Percentage excess |  | Total deaths | Excess deaths | Percentage excess |  | Total deaths | Excess deaths | Percentage excess |  | Total deaths | Excess deaths | Percentage excess |  | Total deaths | Excess deaths | Percentage excess |
| Hokkaido | 56,790 | -790 (-1,490 to -152) | -1.4 (-2.6 to -0.3) |  | 28,604 | -288 (-752 to 129) | -1.0 (-2.6 to 0.5) |  | 28,186 | -616 (-1,015 to -200) | -2.1 (-3.5 to -0.7) |  | 3,405 | 106 (-19 to 217) | 3.2 (-0.6 to 6.8) |  | 5,103 | -205 (-383 to -38) | -3.9 (-7.0 to -0.7) |  | 11,898 | 338 (115 to 547) | 2.9 (1 to 4.8) |  | 20,281 | -340 (-726 to 42) | -1.6 (-3.5 to 0.2) |  | 16,103 | -822 (-1,165 to -517) | -4.9 (-6.7 to -3.1) |
| Aomori | 15,581 | -542 (-833 to -285) | -3.4 (-5.1 to -1.8) |  | 7,783 | -221 (-389 to -71) | -2.8 (-4.8 to -0.9) |  | 7,798 | -319 (-478 to -154) | -3.9 (-5.8 to -1.9) |  | 901 | 13 (-26 to 48) | 1.5 (-2.8 to 5.6) |  | 1,481 | -138 (-213 to -68) | -8.5 (-12.6 to -4.4) |  | 3,228 | 84 (8 to 156) | 2.7 (0.3 to 5.1) |  | 5,891 | -219 (-381 to -73) | -3.6 (-6.1 to -1.2) |  | 4,080 | -248 (-363 to -142) | -5.7 (-8.2 to -3.4) |
| Iwate | 14,846 | -490 (-775 to -236) | -3.2 (-5.0 to -1.6) |  | 7,300 | -256 (-417 to -89) | -3.4 (-5.4 to -1.2) |  | 7,546 | -243 (-399 to -75) | -3.1 (-5.0 to -1.0) |  | 773 | 4 (-30 to 34) | 0.6 (-3.8 to 4.7) |  | 1,264 | -95 (-157 to -37) | -7.0 (-11.0 to -2.8) |  | 2,693 | 37 (-28 to 99) | 1.4 (-1 to 3.8) |  | 5,517 | -194 (-351 to -54) | -3.4 (-6.0 to -1.0) |  | 4,599 | -242 (-366 to -129) | -5.0 (-7.4 to -2.7) |
| Miyagi | 21,396 | -575 (-942 to -249) | -2.6 (-4.2 to -1.2) |  | 10,844 | -192 (-416 to 9) | -1.7 (-3.7 to 0.1) |  | 10,552 | -396 (-610 to -195) | -3.6 (-5.5 to -1.8) |  | 1,351 | 21 (-34 to 71) | 1.6 (-2.4 to 5.5) |  | 1,863 | -140 (-227 to -58) | -7.0 (-10.9 to -3.0) |  | 4,019 | 85 (-6 to 173) | 2.2 (-0.2 to 4.5) |  | 7,711 | -232 (-431 to -43) | -2.9 (-5.3 to -0.6) |  | 6,452 | -369 (-531 to -215) | -5.4 (-7.6 to -3.2) |
| Akita | 13,404 | -262 (-516 to -36) | -1.9 (-3.7 to -0.3) |  | 6,640 | -87 (-228 to 62) | -1.3 (-3.3 to 0.9) |  | 6,764 | -218 (-358 to -74) | -3.1 (-5.0 to -1.1) |  | 593 | 8 (-18 to 31) | 1.4 (-3.0 to 5.6) |  | 1,106 | -62 (-116 to -11) | -5.3 (-9.5 to -1.0) |  | 2,353 | 62 (4 to 117) | 2.7 (0.2 to 5.2) |  | 5,027 | -168 (-310 to -38) | -3.2 (-5.8 to -0.7) |  | 4,325 | -200 (-314 to -92) | -4.4 (-6.8 to -2.1) |
| Yamagata | 13,387 | -322 (-573 to -99) | -2.3 (-4.1 to -0.7) |  | 6,409 | -173 (-312 to -29) | -2.6 (-4.6 to -0.5) |  | 6,978 | -165 (-305 to -15) | -2.3 (-4.2 to -0.2) |  | 634 | 26 (-2 to 50) | 4.3 (-0.3 to 8.5) |  | 949 | -77 (-124 to -32) | -7.5 (-11.6 to -3.2) |  | 2,143 | 16 (-35 to 65) | 0.8 (-1.6 to 3.1) |  | 4,926 | -195 (-338 to -68) | -3.8 (-6.4 to -1.4) |  | 4,735 | -177 (-305 to -67) | -3.6 (-6.0 to -1.4) |
| Fukushima | 21,167 | -678 (-1,046 to -356) | -3.1 (-4.7 to -1.7) |  | 10,599 | -271 (-496 to -69) | -2.5 (-4.5 to -0.6) |  | 10,568 | -443 (-658 to -244) | -4.0 (-5.9 to -2.3) |  | 1,147 | 7 (-43 to 50) | 0.6 (-3.6 to 4.6) |  | 1,933 | -124 (-208 to -41) | -6.0 (-9.7 to -2.1) |  | 3,885 | 36 (-52 to 120) | 0.9 (-1.3 to 3.2) |  | 7,477 | -277 (-469 to -99) | -3.6 (-5.9 to -1.3) |  | 6,725 | -284 (-446 to -130) | -4.0 (-6.2 to -1.9) |
| Ibaraki | 28,446 | -762 (-1,198 to -352) | -2.6 (-4.0 to -1.2) |  | 15,068 | -294 (-587 to -32) | -1.9 (-3.7 to -0.2) |  | 13,378 | -493 (-744 to -248) | -3.6 (-5.3 to -1.8) |  | 1,847 | 74 (0 to 138) | 4.2 (0.0 to 8.1) |  | 2,550 | -221 (-331 to -115) | -8.0 (-11.5 to -4.3) |  | 6,266 | 95 (-35 to 220) | 1.5 (-0.6 to 3.6) |  | 9,893 | -383 (-624 to -160) | -3.7 (-5.9 to -1.6) |  | 7,890 | -364 (-550 to -190) | -4.4 (-6.5 to -2.3) |
| Tochigi | 18,811 | -459 (-786 to -171) | -2.4 (-4.0 to -0.9) |  | 9,685 | -132 (-335 to 49) | -1.3 (-3.3 to 0.5) |  | 9,126 | -371 (-563 to -194) | -3.9 (-5.8 to -2.1) |  | 1,208 | 26 (-23 to 71) | 2.2 (-1.8 to 6.2) |  | 1,786 | -92 (-170 to -15) | -4.9 (-8.7 to -0.8) |  | 3,952 | 75 (-14 to 160) | 1.9 (-0.4 to 4.2) |  | 6,510 | -202 (-372 to -45) | -3.0 (-5.4 to -0.7) |  | 5,355 | -213 (-353 to -89) | -3.8 (-6.2 to -1.6) |
| Gunma | 20,218 | -328 (-664 to -34) | -1.6 (-3.2 to -0.2) |  | 10,435 | -158 (-370 to 32) | -1.5 (-3.4 to 0.3) |  | 9,783 | -260 (-454 to -77) | -2.6 (-4.4 to -0.8) |  | 1,148 | 34 (-15 to 76) | 3.0 (-1.3 to 7.1) |  | 1,626 | -109 (-186 to -36) | -6.3 (-10.3 to -2.2) |  | 4,242 | 19 (-75 to 110) | 0.5 (-1.7 to 2.7) |  | 7,162 | -199 (-381 to -24) | -2.7 (-5.1 to -0.3) |  | 6,040 | -192 (-336 to -56) | -3.1 (-5.3 to -0.9) |
| Saitama | 61,511 | -523 (-1,280 to 164) | -0.8 (-2.0 to 0.3) |  | 33,578 | -157 (-696 to 325) | -0.5 (-2.0 to 1.0) |  | 27,933 | -405 (-810 to 11) | -1.4 (-2.8 to 0.0) |  | 4,586 | 178 (28 to 330) | 4.0 (0.6 to 7.8) |  | 5,707 | -135 (-332 to 48) | -2.3 (-5.5 to 0.8) |  | 15,256 | 238 (-28 to 482) | 1.6 (-0.2 to 3.3) |  | 22,322 | -364 (-777 to 37) | -1.6 (-3.4 to 0.2) |  | 13,640 | -490 (-779 to -229) | -3.5 (-5.4 to -1.7) |
| Chiba | 53,904 | -408 (-1,080 to 201) | -0.8 (-2.0 to 0.4) |  | 29,286 | -265 (-734 to 153) | -0.9 (-2.4 to 0.5) |  | 24,618 | -318 (-695 to 60) | -1.3 (-2.7 to 0.2) |  | 3,871 | 95 (-45 to 217) | 2.5 (-1.1 to 5.9) |  | 4,781 | -120 (-286 to 38) | -2.4 (-5.7 to 0.8) |  | 12,651 | 113 (-122 to 330) | 0.9 (-1.0 to 2.7) |  | 19,481 | -280 (-626 to 97) | -1.4 (-3.1 to 0.5) |  | 13,120 | -472 (-755 to -219) | -3.5 (-5.4 to -1.6) |
| Tokyo | 105,503 | -844 (-1,935 to 173) | -0.8 (-1.8 to 0.2) |  | 55,227 | -243 (-985 to 443) | -0.4 (-1.8 to 0.8) |  | 50,276 | -810 (-1,407 to -196) | -1.6 (-2.7 to -0.4) |  | 8,082 | 259 (27 to 487) | 3.3 (0.3 to 6.4) |  | 8,954 | -88 (-353 to 151) | -1.0 (-3.8 to 1.7) |  | 22,666 | 295 (-76 to 630) | 1.3 (-0.3 to 2.9) |  | 37,795 | -798 (-1,352 to -181) | -2.1 (-3.5 to -0.5) |  | 28,006 | -594 (-1,071 to -167) | -2.1 (-3.7 to -0.6) |
| Kanagawa | 73,390 | -1,145 (-1,990 to -375) | -1.5 (-2.6 to -0.5) |  | 39,231 | -366 (-943 to 151) | -0.9 (-2.3 to 0.4) |  | 34,159 | -902 (-1,358 to -432) | -2.6 (-3.8 to -1.2) |  | 5,582 | 292 (109 to 478) | 5.5 (2.0 to 9.4) |  | 6,143 | -200 (-403 to -17) | -3.2 (-6.2 to -0.3) |  | 16,395 | 108 (-152 to 363) | 0.7 (-0.9 to 2.3) |  | 26,740 | -663 (-1,094 to -188) | -2.4 (-3.9 to -0.7) |  | 18,530 | -846 (-1,224 to -506) | -4.4 (-6.2 to -2.7) |
| Niigata | 25,746 | -756 (-1,177 to -384) | -2.9 (-4.4 to -1.5) |  | 12,856 | -137 (-387 to 88) | -1.1 (-2.9 to 0.7) |  | 12,890 | -596 (-854 to -355) | -4.4 (-6.2 to -2.7) |  | 1,253 | 30 (-20 to 76) | 2.5 (-1.6 to 6.4) |  | 1,963 | -109 (-194 to -25) | -5.3 (-9.0 to -1.3) |  | 4,639 | 143 (43 to 239) | 3.2 (0.9 to 5.4) |  | 9,289 | -372 (-599 to -155) | -3.9 (-6.1 to -1.6) |  | 8,602 | -605 (-824 to -397) | -6.6 (-8.7 to -4.4) |
| Toyama | 11,338 | -242 (-458 to -50) | -2.1 (-3.9 to -0.4) |  | 5,598 | -72 (-195 to 55) | -1.3 (-3.4 to 1.0) |  | 5,740 | -181 (-307 to -57) | -3.1 (-5.1 to -1.0) |  | 552 | 7 (-17 to 29) | 1.3 (-3.0 to 5.5) |  | 768 | -38 (-77 to -1) | -4.7 (-9.2 to -0.1) |  | 2,301 | 44 (-11 to 96) | 1.9 (-0.5 to 4.4) |  | 4,003 | -168 (-285 to -60) | -4.0 (-6.6 to -1.5) |  | 3,714 | -155 (-253 to -64) | -4.0 (-6.4 to -1.7) |
| Ishikawa | 11,053 | -249 (-460 to -63) | -2.2 (-4.0 to -0.6) |  | 5,531 | -79 (-199 to 45) | -1.4 (-3.5 to 0.8) |  | 5,522 | -182 (-297 to -64) | -3.2 (-5.1 to -1.2) |  | 563 | 17 (-7 to 39) | 3.2 (-1.3 to 7.4) |  | 770 | -38 (-76 to -1) | -4.6 (-9.0 to -0.2) |  | 2,288 | 66 (11 to 119) | 3.0 (0.5 to 5.5) |  | 3,878 | -204 (-321 to -99) | -5.0 (-7.7 to -2.5) |  | 3,554 | -131 (-227 to -42) | -3.6 (-6.0 to -1.2) |
| Fukui | 8,034 | -204 (-370 to -56) | -2.5 (-4.4 to -0.7) |  | 4,016 | -87 (-180 to 8) | -2.1 (-4.3 to 0.2) |  | 4,018 | -125 (-217 to -34) | -3.0 (-5.1 to -0.8) |  | 363 | 8 (-8 to 23) | 2.4 (-2.2 to 6.8) |  | 566 | -32 (-61 to -4) | -5.3 (-9.8 to -0.7) |  | 1,464 | 26 (-10 to 61) | 1.8 (-0.7 to 4.4) |  | 2,879 | -110 (-198 to -29) | -3.7 (-6.4 to -1.0) |  | 2,762 | -117 (-198 to -45) | -4.1 (-6.7 to -1.6) |
| Yamanashi | 8,448 | -224 (-397 to -70) | -2.6 (-4.5 to -0.8) |  | 4,300 | -96 (-197 to 6) | -2.2 (-4.4 to 0.1) |  | 4,148 | -132 (-225 to -33) | -3.1 (-5.1 to -0.8) |  | 465 | 10 (-10 to 28) | 2.2 (-2.1 to 6.5) |  | 636 | -38 (-72 to -6) | -5.6 (-10.2 to -1.0) |  | 1,532 | 15 (-24 to 51) | 1.0 (-1.5 to 3.5) |  | 2,932 | -81 (-171 to -1) | -2.7 (-5.5 to 0.0) |  | 2,883 | -118 (-200 to -42) | -3.9 (-6.5 to -1.4) |
| Nagano | 22,071 | -505 (-866 to -189) | -2.2 (-3.8 to -0.8) |  | 10,971 | -214 (-436 to -15) | -1.9 (-3.8 to -0.1) |  | 11,100 | -325 (-542 to -124) | -2.8 (-4.7 to -1.1) |  | 1,037 | 26 (-17 to 65) | 2.6 (-1.6 to 6.7) |  | 1,433 | -72 (-138 to -8) | -4.8 (-8.8 to -0.6) |  | 3,746 | 88 (3 to 170) | 2.4 (0.1 to 4.8) |  | 7,562 | -243 (-437 to -59) | -3.1 (-5.5 to -0.8) |  | 8,293 | -323 (-518 to -152) | -3.8 (-5.9 to -1.8) |
| Gifu | 19,661 | -585 (-921 to -292) | -2.9 (-4.5 to -1.5) |  | 9,930 | -293 (-492 to -113) | -2.9 (-4.7 to -1.1) |  | 9,731 | -309 (-507 to -126) | -3.1 (-4.9 to -1.3) |  | 992 | 3 (-39 to 41) | 0.3 (-3.8 to 4.3) |  | 1,376 | -83 (-146 to -22) | -5.7 (-9.6 to -1.6) |  | 3,918 | 1 (-82 to 86) | 0.0 (-2.0 to 2.2) |  | 7,403 | -270 (-460 to -89) | -3.5 (-5.8 to -1.2) |  | 5,972 | -222 (-370 to -87) | -3.6 (-5.8 to -1.4) |
| Shizuoka | 36,166 | -1,198 (-1,736 to -727) | -3.2 (-4.6 to -2.0) |  | 18,689 | -502 (-844 to -196) | -2.6 (-4.3 to -1.0) |  | 17,477 | -627 (-938 to -326) | -3.5 (-5.1 to -1.8) |  | 2,038 | 42 (-38 to 112) | 2.1 (-1.9 to 5.8) |  | 2,884 | -169 (-286 to -55) | -5.5 (-9.0 to -1.9) |  | 7,566 | 107 (-47 to 253) | 1.4 (-0.6 to 3.5) |  | 13,010 | -558 (-850 to -275) | -4.1 (-6.1 to -2.1) |  | 10,668 | -607 (-853 to -380) | -5.4 (-7.4 to -3.4) |
| Aichi | 61,214 | -459 (-1,166 to 195) | -0.7 (-1.9 to 0.3) |  | 32,688 | -302 (-804 to 151) | -0.9 (-2.4 to 0.5) |  | 28,526 | -282 (-671 to 119) | -1.0 (-2.3 to 0.4) |  | 4,093 | 166 (23 to 288) | 4.2 (0.6 to 7.6) |  | 4,866 | -273 (-453 to -108) | -5.3 (-8.5 to -2.2) |  | 14,155 | 353 (117 to 584) | 2.6 (0.8 to 4.3) |  | 22,604 | -585 (-1,010 to -174) | -2.5 (-4.3 to -0.8) |  | 15,496 | -345 (-655 to -79) | -2.2 (-4.1 to -0.5) |
| Mie | 17,939 | -397 (-704 to -119) | -2.2 (-3.8 to -0.7) |  | 9,101 | -199 (-395 to -25) | -2.1 (-4.2 to -0.3) |  | 8,838 | -231 (-407 to -68) | -2.6 (-4.4 to -0.8) |  | 993 | 41 (-1 to 78) | 4.3 (-0.1 to 8.5) |  | 1,327 | -59 (-120 to -1) | -4.3 (-8.3 to -0.1) |  | 3,596 | 49 (-33 to 128) | 1.4 (-0.9 to 3.7) |  | 6,493 | -306 (-468 to -137) | -4.5 (-6.7 to -2.1) |  | 5,530 | -171 (-306 to -42) | -3.0 (-5.2 to -0.8) |
| Shiga | 11,295 | -276 (-495 to -82) | -2.4 (-4.2 to -0.7) |  | 5,761 | -121 (-246 to 11) | -2.1 (-4.1 to 0.2) |  | 5,534 | -151 (-272 to -32) | -2.7 (-4.7 to -0.6) |  | 631 | 13 (-15 to 37) | 2.1 (-2.3 to 6.2) |  | 883 | -39 (-83 to 2) | -4.2 (-8.6 to 0.3) |  | 2,288 | 28 (-27 to 80) | 1.2 (-1.2 to 3.6) |  | 3,971 | -137 (-254 to -33) | -3.3 (-6.0 to -0.8) |  | 3,522 | -154 (-250 to -66) | -4.2 (-6.6 to -1.8) |
| Kyoto | 23,267 | -461 (-837 to -132) | -1.9 (-3.5 to -0.6) |  | 11,889 | -193 (-432 to 22) | -1.6 (-3.5 to 0.2) |  | 11,378 | -314 (-528 to -107) | -2.7 (-4.4 to -0.9) |  | 1,364 | 49 (-8 to 99) | 3.8 (-0.6 to 7.8) |  | 1,645 | -93 (-168 to -22) | -5.4 (-9.3 to -1.3) |  | 5,068 | 96 (-13 to 199) | 1.9 (-0.3 to 4.1) |  | 8,261 | -365 (-578 to -162) | -4.2 (-6.5 to -1.9) |  | 6,929 | -299 (-462 to -147) | -4.1 (-6.3 to -2.1) |
| Osaka | 79,626 | -127 (-960 to 652) | -0.2 (-1.2 to 0.8) |  | 42,553 | 114 (-452 to 628) | 0.3 (-1.0 to 1.5) |  | 37,073 | -437 (-917 to 65) | -1.2 (-2.4 to 0.2) |  | 5,521 | 166 (-3 to 333) | 3.1 (0.0 to 6.4) |  | 6,591 | -278 (-484 to -65) | -4.0 (-6.8 to -1.0) |  | 19,314 | 302 (-19 to 591) | 1.6 (-0.1 to 3.2) |  | 29,651 | -93 (-581 to 367) | -0.3 (-1.9 to 1.3) |  | 18,549 | -556 (-918 to -239) | -2.9 (-4.7 to -1.3) |
| Hyogo | 51,026 | 104 (-531 to 678) | 0.2 (-1.0 to 1.3) |  | 26,346 | 192 (-234 to 572) | 0.7 (-0.9 to 2.2) |  | 24,680 | -205 (-557 to 162) | -0.8 (-2.2 to 0.7) |  | 3,069 | 80 (-32 to 179) | 2.7 (-1.0 to 6.2) |  | 3,912 | -155 (-300 to -21) | -3.8 (-7.1 to -0.5) |  | 10,960 | 330 (125 to 522) | 3.1 (1.1 to 5.0) |  | 18,647 | -49 (-401 to 294) | -0.3 (-2.1 to 1.6) |  | 14,438 | -323 (-623 to -46) | -2.2 (-4.1 to -0.3) |
| Nara | 12,692 | -318 (-557 to -108) | -2.4 (-4.2 to -0.8) |  | 6,510 | -70 (-219 to 60) | -1.1 (-3.3 to 0.9) |  | 6,182 | -240 (-380 to -113) | -3.7 (-5.8 to -1.8) |  | 709 | 24 (-7 to 51) | 3.4 (-1.0 to 7.7) |  | 951 | -48 (-94 to -3) | -4.8 (-9.0 to -0.3) |  | 2,692 | 37 (-27 to 98) | 1.4 (-1.0 to 3.8) |  | 4,529 | -210 (-345 to -90) | -4.4 (-7.1 to -1.9) |  | 3,811 | -126 (-226 to -32) | -3.2 (-5.6 to -0.8) |
| Wakayama | 10,869 | -263 (-476 to -74) | -2.4 (-4.2 to -0.7) |  | 5,354 | -120 (-241 to 3) | -2.2 (-4.3 to 0.1) |  | 5,515 | -145 (-265 to -26) | -2.6 (-4.6 to -0.5) |  | 528 | 9 (-15 to 29) | 1.7 (-2.8 to 5.9) |  | 819 | -41 (-81 to -1) | -4.7 (-9.0 to -0.1) |  | 2,079 | 19 (-32 to 67) | 0.9 (-1.5 to 3.3) |  | 3,960 | -119 (-232 to -14) | -2.9 (-5.5 to -0.4) |  | 3,483 | -151 (-248 to -64) | -4.2 (-6.6 to -1.8) |
| Tottori | 6,133 | -237 (-373 to -115) | -3.7 (-5.7 to -1.8) |  | 2,964 | -90 (-164 to -14) | -2.9 (-5.2 to -0.5) |  | 3,169 | -134 (-210 to -60) | -4.1 (-6.2 to -1.9) |  | 284 | 5 (-8 to 16) | 1.7 (-2.9 to 6.0) |  | 505 | -24 (-51 to 2) | -4.5 (-9.2 to 0.4) |  | 1,062 | 11 (-16 to 37) | 1.1 (-1.5 to 3.7) |  | 2,088 | -105 (-169 to -38) | -4.8 (-7.5 to -1.8) |  | 2,194 | -123 (-188 to -64) | -5.3 (-7.9 to -2.8) |
| Shimane | 8,295 | -191 (-358 to -40) | -2.2 (-4.1 to -0.5) |  | 3,962 | -63 (-156 to 31) | -1.6 (-3.8 to 0.8) |  | 4,333 | -138 (-231 to -41) | -3.1 (-5.1 to -0.9) |  | 365 | 14 (-2 to 29) | 4.1 (-0.7 to 8.6) |  | 568 | -21 (-49 to 9) | -3.5 (-8.0 to 1.7) |  | 1,388 | 30 (-5 to 63) | 2.2 (-0.4 to 4.7) |  | 2,835 | -128 (-214 to -48) | -4.3 (-7.0 to -1.6) |  | 3,139 | -116 (-203 to -38) | -3.6 (-6.1 to -1.2) |
| Okayama | 18,952 | -195 (-516 to 86) | -1.0 (-2.7 to 0.5) |  | 9,549 | -41 (-240 to 138) | -0.4 (-2.5 to 1.5) |  | 9,403 | -186 (-379 to -15) | -1.9 (-3.9 to -0.2) |  | 930 | 14 (-26 to 49) | 1.6 (-2.7 to 5.6) |  | 1,339 | -78 (-142 to -17) | -5.5 (-9.6 to -1.2) |  | 3,674 | 100 (15 to 181) | 2.8 (0.4 to 5.2) |  | 6,760 | -148 (-321 to 17) | -2.1 (-4.5 to 0.3) |  | 6,249 | -157 (-304 to -19) | -2.5 (-4.6 to -0.3) |
| Hiroshima | 26,255 | -893 (-1,335 to -506) | -3.3 (-4.8 to -1.9) |  | 13,242 | -490 (-765 to -244) | -3.6 (-5.5 to -1.8) |  | 13,013 | -446 (-692 to -207) | -3.3 (-5.0 to -1.6) |  | 1,501 | 39 (-23 to 94) | 2.7 (-1.5 to 6.6) |  | 2,001 | -140 (-230 to -54) | -6.6 (-10.3 to -2.6) |  | 5,437 | 62 (-54 to 173) | 1.2 (-1.0 to 3.3) |  | 8,880 | -497 (-729 to -282) | -5.3 (-7.6 to -3.1) |  | 8,436 | -418 (-623 to -231) | -4.7 (-6.9 to -2.7) |
| Yamaguchi | 15,998 | -590 (-884 to -331) | -3.6 (-5.2 to -2.0) |  | 7,935 | -204 (-381 to -48) | -2.5 (-4.6 to -0.6) |  | 8,063 | -371 (-545 to -208) | -4.4 (-6.3 to -2.5) |  | 738 | 19 (-13 to 48) | 2.7 (-1.8 to 6.9) |  | 1,213 | -87 (-146 to -29) | -6.7 (-10.7 to -2.4) |  | 3,185 | 32 (-44 to 103) | 1.0 (-1.4 to 3.3) |  | 5,653 | -303 (-465 to -162) | -5.1 (-7.6 to -2.8) |  | 5,209 | -236 (-367 to -112) | -4.3 (-6.6 to -2.1) |
| Tokushima | 8,525 | -302 (-480 to -143) | -3.4 (-5.3 to -1.7) |  | 4,141 | -116 (-213 to -17) | -2.7 (-4.9 to -0.4) |  | 4,384 | -168 (-275 to -75) | -3.7 (-5.9 to -1.7) |  | 429 | 13 (-5 to 30) | 3.2 (-1.2 to 7.5) |  | 655 | -44 (-79 to -11) | -6.3 (-10.8 to -1.7) |  | 1,584 | 23 (-16 to 61) | 1.5 (-1.0 to 4.0) |  | 3,096 | -134 (-228 to -47) | -4.2 (-6.9 to -1.5) |  | 2,761 | -150 (-232 to -77) | -5.2 (-7.8 to -2.7) |
| Kagawa | 10,587 | -176 (-380 to 5) | -1.6 (-3.5 to 0.1) |  | 5,331 | -33 (-150 to 88) | -0.6 (-2.7 to 1.7) |  | 5,256 | -156 (-266 to -42) | -2.9 (-4.8 to -0.8) |  | 529 | 17 (-6 to 38) | 3.4 (-1.2 to 7.7) |  | 805 | -50 (-92 to -11) | -5.9 (-10.3 to -1.4) |  | 2,035 | 58 (9 to 105) | 2.9 (0.4 to 5.4) |  | 3,645 | -136 (-244 to -37) | -3.6 (-6.3 to -1.0) |  | 3,573 | -115 (-209 to -27) | -3.1 (-5.5 to -0.8) |
| Ehime | 15,507 | -485 (-769 to -236) | -3.0 (-4.7 to -1.5) |  | 7,562 | -180 (-350 to -31) | -2.3 (-4.4 to -0.4) |  | 7,945 | -286 (-453 to -128) | -3.5 (-5.4 to -1.6) |  | 792 | 22 (-11 to 53) | 2.9 (-1.4 to 7.1) |  | 1,256 | -81 (-143 to -25) | -6.1 (-10.2 to -2.0) |  | 2,975 | 29 (-41 to 96) | 1.0 (-1.4 to 3.3) |  | 5,415 | -235 (-386 to -101) | -4.2 (-6.6 to -1.8) |  | 5,069 | -210 (-341 to -93) | -4.0 (-6.3 to -1.8) |
| Kochi | 8,578 | -213 (-386 to -47) | -2.4 (-4.3 to -0.5) |  | 4,215 | -89 (-183 to 11) | -2.1 (-4.2 to 0.3) |  | 4,363 | -132 (-226 to -35) | -2.9 (-4.9 to -0.8) |  | 402 | 6 (-13 to 22) | 1.5 (-3.1 to 5.7) |  | 641 | -37 (-71 to -6) | -5.5 (-10.0 to -0.9) |  | 1,590 | 36 (-4 to 74) | 2.3 (-0.3 to 4.9) |  | 2,959 | -104 (-195 to -23) | -3.4 (-6.2 to -0.8) |  | 2,986 | -126 (-210 to -49) | -4.1 (-6.6 to -1.6) |
| Fukuoka | 46,226 | -1,377 (-2,013 to -807) | -2.9 (-4.2 to -1.7) |  | 22,970 | -658 (-1,069 to -291) | -2.8 (-4.4 to -1.3) |  | 23,256 | -711 (-1,063 to -340) | -3.0 (-4.4 to -1.4) |  | 2,867 | 57 (-50 to 150) | 2.0 (-1.7 to 5.5) |  | 4,046 | -318 (-474 to -165) | -7.3 (-10.5 to -3.9) |  | 9,602 | 104 (-85 to 282) | 1.1 (-0.9 to 3.0) |  | 16,041 | -680 (-1,019 to -358) | -4.1 (-6.0 to -2.2) |  | 13,670 | -621 (-912 to -358) | -4.3 (-6.3 to -2.5) |
| Saga | 8,603 | -179 (-345 to -26) | -2.0 (-3.9 to -0.3) |  | 4,168 | -77 (-174 to 22) | -1.8 (-4.0 to 0.5) |  | 4,435 | -118 (-213 to -20) | -2.6 (-4.6 to -0.4) |  | 442 | 11 (-9 to 29) | 2.6 (-2.0 to 6.9) |  | 722 | -33 (-71 to 2) | -4.4 (-8.9 to 0.2) |  | 1,482 | 22 (-16 to 58) | 1.5 (-1.0 to 4.1) |  | 3,057 | -101 (-195 to -19) | -3.2 (-6.0 to -0.6) |  | 2,900 | -126 (-207 to -53) | -4.2 (-6.7 to -1.8) |
| Nagasaki | 15,402 | -230 (-502 to 10) | -1.5 (-3.2 to 0.1) |  | 7,444 | -114 (-282 to 33) | -1.5 (-3.6 to 0.5) |  | 7,958 | -149 (-311 to 4) | -1.8 (-3.8 to 0.1) |  | 828 | 36 (2 to 68) | 4.6 (0.2 to 8.9) |  | 1,332 | -50 (-112 to 9) | -3.6 (-7.8 to 0.7) |  | 2,746 | 23 (-43 to 86) | 0.8 (-1.6 to 3.2) |  | 5,477 | -116 (-262 to 18) | -2.1 (-4.6 to 0.3) |  | 5,019 | -195 (-324 to -78) | -3.7 (-6.1 to -1.5) |
| Kumamoto | 18,324 | -575 (-888 to -284) | -3.0 (-4.6 to -1.5) |  | 8,989 | -155 (-348 to 17) | -1.7 (-3.7 to 0.2) |  | 9,335 | -391 (-573 to -200) | -4.0 (-5.8 to -2.1) |  | 960 | 40 (0 to 76) | 4.4 (0.0 to 8.6) |  | 1,430 | -72 (-137 to -8) | -4.8 (-8.7 to -0.5) |  | 3,008 | 84 (18 to 152) | 2.9 (0.6 to 5.3) |  | 6,452 | -281 (-454 to -115) | -4.2 (-6.6 to -1.8) |  | 6,474 | -336 (-498 to -191) | -4.9 (-7.1 to -2.9) |
| Oita | 12,488 | -230 (-468 to -19) | -1.8 (-3.6 to -0.2) |  | 6,181 | -22 (-152 to 115) | -0.4 (-2.4 to 1.9) |  | 6,307 | -227 (-359 to -91) | -3.5 (-5.4 to -1.4) |  | 563 | 11 (-14 to 33) | 2.0 (-2.5 to 6.2) |  | 935 | -54 (-101 to -10) | -5.5 (-9.8 to -1.1) |  | 2,289 | 80 (25 to 133) | 3.6 (1.1 to 6.2) |  | 4,413 | -156 (-281 to -40) | -3.4 (-6.0 to -0.9) |  | 4,288 | -240 (-357 to -132) | -5.3 (-7.7 to -3.0) |
| Miyazaki | 12,278 | -93 (-324 to 110) | -0.8 (-2.6 to 0.9) |  | 6,083 | -23 (-155 to 113) | -0.4 (-2.5 to 1.9) |  | 6,195 | -119 (-259 to 5) | -1.9 (-4.0 to 0.1) |  | 665 | 20 (-9 to 46) | 3.1 (-1.4 to 7.4) |  | 1,076 | -17 (-70 to 32) | -1.6 (-6.1 to 3.0) |  | 2,176 | 52 (-1 to 102) | 2.4 (0.0 to 4.9) |  | 4,365 | -133 (-256 to -20) | -3.0 (-5.5 to -0.5) |  | 3,996 | -122 (-228 to -24) | -3.0 (-5.4 to -0.6) |
| Kagoshima | 18,602 | -361 (-668 to -61) | -1.9 (-3.5 to -0.3) |  | 9,003 | -189 (-382 to -19) | -2.1 (-4.1 to -0.2) |  | 9,599 | -231 (-425 to -54) | -2.3 (-4.2 to -0.6) |  | 961 | 27 (-14 to 63) | 2.9 (-1.4 to 7.0) |  | 1,648 | -89 (-164 to -18) | -5.1 (-9.1 to -1.1) |  | 3,093 | 68 (-4 to 136) | 2.2 (-0.1 to 4.6) |  | 6,476 | -206 (-377 to -43) | -3.1 (-5.5 to -0.7) |  | 6,424 | -240 (-394 to -95) | -3.6 (-5.8 to -1.5) |
| Okinawa | 10,857 | -364 (-580 to -169) | -3.2 (-5.1 to -1.5) |  | 5,849 | -191 (-325 to -53) | -3.2 (-5.3 to -0.9) |  | 5,008 | -157 (-269 to -45) | -3.0 (-5.1 to -0.9) |  | 1,133 | 21 (-27 to 64) | 1.9 (-2.3 to 5.9) |  | 1,344 | -99 (-165 to -37) | -6.8 (-10.9 to -2.7) |  | 1,892 | 35 (-12 to 80) | 1.9 (-0.7 to 4.4) |  | 3,565 | -149 (-255 to -50) | -4.0 (-6.7 to -1.4) |  | 2,923 | -156 (-241 to -81) | -5.1 (-7.6 to -2.7) |
| Japan | 1,190,409 | -20,982 (-38,367 to -5,472) | -1.7 (-3.1 to -0.5) |  | 612,370 | -8,021 (-15,155 to -1,779) | -1.3 (-2.4 to -0.3) |  | 578,039 | -14,561 (-19,506 to -9,685) | -2.5 (-3.3 to -1.6) |  | 73,688 | 2,213 (386 to 3,895) | 3.1 (0.5 to 5.6) |  | 98,152 | -4,662 (-7,972 to -1,492) | -4.5 (-7.5 to -1.5) |  | 248,471 | 4,153 (23 to 8,080) | 1.7 (0.0 to 3.4) |  | 426,982 | -11,995 (-18,517 to -6,138) | -2.7 (-4.2 to -1.4) |  | 343,116 | -13,604 (-20,585 to -7,189) | -3.8 (-5.7 to -2.1) |

**Table S3.** Sensitivity analysis without adjusting for influenza activity and ambient temperature: Number of observed and estimated excess deaths (95% empirical confidence interval) during the period 13 February–31 December 2020 in Japan.

| Prefecture | Total | | |
| --- | --- | --- | --- |
|  | Total deaths | Excess deaths | Percentage excess |
| Hokkaido | 56,790 | -1,394 (-2,067 to -749) | -2.4 (-3.5 to -1.4) |
| Aomori | 15,581 | -547 (-810 to -296) | -1.8 (-3.1 to -0.6) |
| Iwate | 14,846 | -641 (-901 to -391) | -2.4 (-4.0 to -0.8) |
| Miyagi | 21,396 | -700 (-1,028 to -404) | -2.8 (-4.5 to -1.3) |
| Akita | 13,404 | -388 (-622 to -163) | -2.1 (-3.6 to -0.6) |
| Yamagata | 13,387 | -342 (-574 to -119) | -1.5 (-3.2 to 0.1) |
| Fukushima | 21,167 | -727 (-1,058 to -427) | -2.1 (-3.8 to -0.5) |
| Ibaraki | 28,446 | -931 (-1,342 to -555) | -2.3 (-3.8 to -0.9) |
| Tochigi | 18,811 | -574 (-874 to -287) | -2.2 (-3.6 to -0.8) |
| Gunma | 20,218 | -435 (-750 to -152) | -1.6 (-3.1 to -0.1) |
| Saitama | 61,511 | -730 (-1,470 to -29) | -1.6 (-3.1 to -0.1) |
| Chiba | 53,904 | -904 (-1,554 to -312) | -1.2 (-2.5 to 0.1) |
| Tokyo | 105,503 | -1,861 (-2,985 to -754) | -1.5 (-2.8 to -0.2) |
| Kanagawa | 73,390 | -1,556 (-2,327 to -760) | -1.4 (-2.6 to -0.3) |
| Niigata | 25,746 | -1,003 (-1,373 to -662) | -1.3 (-2.5 to -0.1) |
| Toyama | 11,338 | -287 (-488 to -95) | -1.9 (-3.4 to -0.5) |
| Ishikawa | 11,053 | -286 (-468 to -91) | -1.4 (-3.1 to 0.3) |
| Fukui | 8,034 | -237 (-396 to -87) | -1.4 (-3.1 to 0.2) |
| Yamanashi | 8,448 | -233 (-398 to -77) | -2.1 (-3.9 to -0.5) |
| Nagano | 22,071 | -667 (-992 to -342) | -2.0 (-3.7 to -0.3) |
| Gifu | 19,661 | -664 (-972 to -386) | -2.2 (-3.7 to -0.8) |
| Shizuoka | 36,166 | -1,129 (-1,615 to -678) | -2.5 (-4.0 to -1.1) |
| Aichi | 61,214 | -702 (-1,411 to -58) | -2.2 (-3.6 to -0.9) |
| Mie | 17,939 | -370 (-658 to -113) | -1.2 (-2.5 to 0.0) |
| Shiga | 11,295 | -288 (-494 to -91) | -1.9 (-3.6 to -0.3) |
| Kyoto | 23,267 | -517 (-869 to -179) | -1.9 (-3.6 to -0.2) |
| Osaka | 79,626 | -459 (-1,286 to 374) | -1.7 (-3.2 to -0.1) |
| Hyogo | 51,026 | -59 (-662 to 493) | -0.1 (-1.3 to 1.1) |
| Nara | 12,692 | -310 (-534 to -98) | 0.0 (-1.3 to 1.3) |
| Wakayama | 10,869 | -329 (-528 to -138) | -1.7 (-3.3 to -0.1) |
| Tottori | 6,133 | -247 (-376 to -127) | -2.3 (-4.1 to -0.7) |
| Shimane | 8,295 | -181 (-341 to -35) | -2.2 (-4.0 to -0.5) |
| Okayama | 18,952 | -311 (-609 to -44) | -1.7 (-3.5 to 0.1) |
| Hiroshima | 26,255 | -1,034 (-1,429 to -660) | -1.1 (-2.8 to 0.5) |
| Yamaguchi | 15,998 | -571 (-839 to -332) | -2.9 (-4.4 to -1.5) |
| Tokushima | 8,525 | -265 (-432 to -106) | -1.9 (-3.5 to -0.4) |
| Kagawa | 10,587 | -221 (-414 to -36) | -2.0 (-3.7 to -0.4) |
| Ehime | 15,507 | -478 (-748 to -235) | -1.2 (-3.0 to 0.5) |
| Kochi | 8,578 | -255 (-421 to -97) | -2.1 (-3.7 to -0.6) |
| Fukuoka | 46,226 | -1,563 (-2,139 to -1,040) | -2.0 (-3.7 to -0.3) |
| Saga | 8,603 | -211 (-376 to -60) | -2.5 (-3.8 to -1.2) |
| Nagasaki | 15,402 | -288 (-541 to -63) | -1.8 (-3.5 to -0.1) |
| Kumamoto | 18,324 | -586 (-882 to -321) | -1.6 (-3.2 to -0.1) |
| Oita | 12,488 | -299 (-523 to -84) | -1.7 (-3.3 to -0.2) |
| Miyazaki | 12,278 | -79 (-291 to 112) | -1.0 (-2.7 to 0.6) |
| Kagoshima | 18,602 | -480 (-780 to -209) | -0.8 (-2.6 to 0.8) |
| Okinawa | 10,857 | -358 (-557 to -166) | -1.8 (-3.4 to -0.3) |
| Japan | 1,190,409 | -26,697 (-36,292 to -18,177) | -2.2 (-3.0 to -1.5) |

**Table S4.** Sensitivity analysis with adjusting for influenza activity: Number of observed and estimated excess deaths (95% empirical confidence interval) during the period 13 February–31 December 2020 in Japan.

| Prefecture | Total | | |
| --- | --- | --- | --- |
|  | Total deaths | Excess deaths | Percentage excess |
| Hokkaido | 56,790 | -970 (-1,630 to -318) | -1.7 (-2.8 to -0.6) |
| Aomori | 15,581 | -446 (-703 to -199) | -2.8 (-4.3 to -1.3) |
| Iwate | 14,846 | -518 (-776 to -283) | -3.4 (-5.0 to -1.9) |
| Miyagi | 21,396 | -591 (-936 to -287) | -2.7 (-4.2 to -1.3) |
| Akita | 13,404 | -277 (-514 to -63) | -2.0 (-3.7 to -0.5) |
| Yamagata | 13,387 | -268 (-500 to -57) | -2.0 (-3.6 to -0.4) |
| Fukushima | 21,167 | -614 (-953 to -307) | -2.8 (-4.3 to -1.4) |
| Ibaraki | 28,446 | -635 (-1,035 to -242) | -2.2 (-3.5 to -0.8) |
| Tochigi | 18,811 | -447 (-739 to -167) | -2.3 (-3.8 to -0.9) |
| Gunma | 20,218 | -302 (-608 to -4) | -1.5 (-2.9 to 0.0) |
| Saitama | 61,511 | -385 (-1,082 to 343) | -0.6 (-1.7 to 0.6) |
| Chiba | 53,904 | -479 (-1,099 to 167) | -0.9 (-2.0 to 0.3) |
| Tokyo | 105,503 | -932 (-1,994 to 178) | -0.9 (-1.9 to 0.2) |
| Kanagawa | 73,390 | -924 (-1,692 to -126) | -1.2 (-2.3 to -0.2) |
| Niigata | 25,746 | -768 (-1,132 to -411) | -2.9 (-4.2 to -1.6) |
| Toyama | 11,338 | -213 (-408 to -22) | -1.8 (-3.5 to -0.2) |
| Ishikawa | 11,053 | -222 (-420 to -42) | -2.0 (-3.7 to -0.4) |
| Fukui | 8,034 | -182 (-334 to -40) | -2.2 (-4.0 to -0.5) |
| Yamanashi | 8,448 | -163 (-324 to -19) | -1.9 (-3.7 to -0.2) |
| Nagano | 22,071 | -473 (-794 to -157) | -2.1 (-3.5 to -0.7) |
| Gifu | 19,661 | -530 (-831 to -242) | -2.6 (-4.1 to -1.2) |
| Shizuoka | 36,166 | -962 (-1,439 to -488) | -2.6 (-3.8 to -1.3) |
| Aichi | 61,214 | -264 (-964 to 420) | -0.4 (-1.6 to 0.7) |
| Mie | 17,939 | -238 (-516 to 33) | -1.3 (-2.8 to 0.2) |
| Shiga | 11,295 | -208 (-412 to -22) | -1.8 (-3.5 to -0.2) |
| Kyoto | 23,267 | -382 (-723 to -50) | -1.6 (-3.0 to -0.2) |
| Osaka | 79,626 | 55 (-753 to 897) | 0.1 (-0.9 to 1.1) |
| Hyogo | 51,026 | 154 (-426 to 759) | 0.3 (-0.8 to 1.5) |
| Nara | 12,692 | -254 (-476 to -50) | -2.0 (-3.6 to -0.4) |
| Wakayama | 10,869 | -267 (-464 to -88) | -2.4 (-4.1 to -0.8) |
| Tottori | 6,133 | -203 (-325 to -90) | -3.2 (-5.0 to -1.4) |
| Shimane | 8,295 | -140 (-294 to 3) | -1.7 (-3.4 to 0.0) |
| Okayama | 18,952 | -192 (-483 to 89) | -1.0 (-2.5 to 0.5) |
| Hiroshima | 26,255 | -894 (-1,273 to -521) | -3.3 (-4.6 to -1.9) |
| Yamaguchi | 15,998 | -471 (-732 to -222) | -2.9 (-4.4 to -1.4) |
| Tokushima | 8,525 | -238 (-402 to -90) | -2.7 (-4.5 to -1.0) |
| Kagawa | 10,587 | -156 (-344 to 16) | -1.5 (-3.1 to 0.2) |
| Ehime | 15,507 | -380 (-644 to -142) | -2.4 (-4.0 to -0.9) |
| Kochi | 8,578 | -185 (-348 to -38) | -2.1 (-3.9 to -0.4) |
| Fukuoka | 46,226 | -1,267 (-1,824 to -689) | -2.7 (-3.8 to -1.5) |
| Saga | 8,603 | -152 (-309 to 0) | -1.7 (-3.5 to 0.0) |
| Nagasaki | 15,402 | -187 (-435 to 48) | -1.2 (-2.7 to 0.3) |
| Kumamoto | 18,324 | -478 (-766 to -199) | -2.5 (-4.0 to -1.1) |
| Oita | 12,488 | -218 (-437 to -17) | -1.7 (-3.4 to -0.1) |
| Miyazaki | 12,278 | -7 (-217 to 184) | -0.1 (-1.7 to 1.5) |
| Kagoshima | 18,602 | -349 (-639 to -67) | -1.8 (-3.3 to -0.4) |
| Okinawa | 10,857 | -255 (-452 to -75) | -2.3 (-4.0 to -0.7) |
| Japan | 1,190,409 | -18,476 (-24,939 to -11,957) | -1.5 (-2.1 to -1.0) |

**Table S5.** Sensitivity analysis with adjusting for ambient temperature: Number of observed and estimated excess deaths (95% empirical confidence interval) during the period 13 February–31 December 2020 in Japan.

| Prefecture | Total | | |
| --- | --- | --- | --- |
|  | Total deaths | Excess deaths | Percentage excess |
| Hokkaido | 56,790 | -1,348 (-1,974 to -706) | -2.3 (-3.4 to -1.2) |
| Aomori | 15,581 | -593 (-866 to -359) | -3.7 (-5.3 to -2.3) |
| Iwate | 14,846 | -626 (-895 to -388) | -4.0 (-5.7 to -2.5) |
| Miyagi | 21,396 | -726 (-1,031 to -404) | -3.3 (-4.6 to -1.9) |
| Akita | 13,404 | -353 (-596 to -144) | -2.6 (-4.3 to -1.1) |
| Yamagata | 13,387 | -349 (-578 to -127) | -2.5 (-4.1 to -0.9) |
| Fukushima | 21,167 | -832 (-1,164 to -520) | -3.8 (-5.2 to -2.4) |
| Ibaraki | 28,446 | -1,035 (-1,417 to -635) | -3.5 (-4.7 to -2.2) |
| Tochigi | 18,811 | -575 (-884 to -311) | -3.0 (-4.5 to -1.6) |
| Gunma | 20,218 | -417 (-726 to -122) | -2.0 (-3.5 to -0.6) |
| Saitama | 61,511 | -881 (-1,612 to -204) | -1.4 (-2.6 to -0.3) |
| Chiba | 53,904 | -1,016 (-1,614 to -401) | -1.8 (-2.9 to -0.7) |
| Tokyo | 105,503 | -1,920 (-2,936 to -886) | -1.8 (-2.7 to -0.8) |
| Kanagawa | 73,390 | -1,676 (-2,424 to -912) | -2.2 (-3.2 to -1.2) |
| Niigata | 25,746 | -913 (-1,258 to -548) | -3.4 (-4.7 to -2.1) |
| Toyama | 11,338 | -273 (-481 to -93) | -2.4 (-4.1 to -0.8) |
| Ishikawa | 11,053 | -306 (-512 to -128) | -2.7 (-4.4 to -1.1) |
| Fukui | 8,034 | -248 (-412 to -106) | -3.0 (-4.9 to -1.3) |
| Yamanashi | 8,448 | -271 (-442 to -123) | -3.1 (-5.0 to -1.4) |
| Nagano | 22,071 | -662 (-968 to -339) | -2.9 (-4.2 to -1.5) |
| Gifu | 19,661 | -729 (-1,036 to -437) | -3.6 (-5.0 to -2.2) |
| Shizuoka | 36,166 | -1,459 (-1,921 to -974) | -3.9 (-5.0 to -2.6) |
| Aichi | 61,214 | -830 (-1,473 to -171) | -1.3 (-2.4 to -0.3) |
| Mie | 17,939 | -451 (-753 to -189) | -2.5 (-4.0 to -1.0) |
| Shiga | 11,295 | -325 (-537 to -142) | -2.8 (-4.5 to -1.2) |
| Kyoto | 23,267 | -562 (-881 to -224) | -2.4 (-3.6 to -1.0) |
| Osaka | 79,626 | -558 (-1,328 to 230) | -0.7 (-1.6 to 0.3) |
| Hyogo | 51,026 | -44 (-600 to 529) | -0.1 (-1.2 to 1.0) |
| Nara | 12,692 | -374 (-607 to -174) | -2.9 (-4.6 to -1.3) |
| Wakayama | 10,869 | -298 (-498 to -107) | -2.7 (-4.4 to -1.0) |
| Tottori | 6,133 | -264 (-396 to -150) | -4.1 (-6.1 to -2.4) |
| Shimane | 8,295 | -217 (-376 to -66) | -2.6 (-4.3 to -0.8) |
| Okayama | 18,952 | -263 (-558 to 21) | -1.4 (-2.9 to 0.1) |
| Hiroshima | 26,255 | -1,098 (-1,461 to -714) | -4.0 (-5.3 to -2.6) |
| Yamaguchi | 15,998 | -663 (-932 to -407) | -4.0 (-5.5 to -2.5) |
| Tokushima | 8,525 | -314 (-484 to -155) | -3.5 (-5.4 to -1.8) |
| Kagawa | 10,587 | -233 (-432 to -60) | -2.2 (-3.9 to -0.6) |
| Ehime | 15,507 | -556 (-833 to -320) | -3.5 (-5.1 to -2.0) |
| Kochi | 8,578 | -278 (-451 to -129) | -3.1 (-5.0 to -1.5) |
| Fukuoka | 46,226 | -1,640 (-2,195 to -1,056) | -3.4 (-4.5 to -2.2) |
| Saga | 8,603 | -219 (-378 to -67) | -2.5 (-4.2 to -0.8) |
| Nagasaki | 15,402 | -321 (-585 to -94) | -2.0 (-3.7 to -0.6) |
| Kumamoto | 18,324 | -671 (-983 to -404) | -3.5 (-5.1 to -2.2) |
| Oita | 12,488 | -316 (-535 to -105) | -2.5 (-4.1 to -0.8) |
| Miyazaki | 12,278 | -171 (-384 to 33) | -1.4 (-3.0 to 0.3) |
| Kagoshima | 18,602 | -528 (-842 to -260) | -2.8 (-4.3 to -1.4) |
| Okinawa | 10,857 | -433 (-640 to -254) | -3.8 (-5.6 to -2.3) |
| Japan | 1,190,409 | -28,834 (-33,025 to -24,510) | -2.4 (-2.7 to -2.0) |

**Table S6.** Sensitivity analysis with 4 knots in the interrupted spline component: Number of observed and estimated excess deaths (95% empirical confidence interval) during the period 13 February–31 December 2020 in Japan.

| Prefecture | Total | | |
| --- | --- | --- | --- |
|  | Total deaths | Excess deaths | Percentage excess |
| Hokkaido | 56,790 | -930 (-1,598 to -262) | -1.6 (-2.7 to -0.5) |
| Aomori | 15,581 | -498 (-755 to -253) | -3.1 (-4.6 to -1.6) |
| Iwate | 14,846 | -535 (-790 to -289) | -3.5 (-5.1 to -1.9) |
| Miyagi | 21,396 | -667 (-991 to -343) | -3.0 (-4.4 to -1.6) |
| Akita | 13,404 | -334 (-563 to -114) | -2.4 (-4.0 to -0.8) |
| Yamagata | 13,387 | -305 (-530 to -85) | -2.2 (-3.8 to -0.6) |
| Fukushima | 21,167 | -687 (-1,005 to -363) | -3.1 (-4.5 to -1.7) |
| Ibaraki | 28,446 | -732 (-1,131 to -340) | -2.5 (-3.8 to -1.2) |
| Tochigi | 18,811 | -501 (-793 to -225) | -2.6 (-4.0 to -1.2) |
| Gunma | 20,218 | -328 (-637 to -35) | -1.6 (-3.1 to -0.2) |
| Saitama | 61,511 | -496 (-1,213 to 209) | -0.8 (-1.9 to 0.3) |
| Chiba | 53,904 | -612 (-1,241 to 23) | -1.1 (-2.3 to 0.0) |
| Tokyo | 105,503 | -1,281 (-2,333 to -218) | -1.2 (-2.2 to -0.2) |
| Kanagawa | 73,390 | -1,182 (-1,960 to -414) | -1.6 (-2.6 to -0.6) |
| Niigata | 25,746 | -750 (-1,117 to -385) | -2.8 (-4.2 to -1.5) |
| Toyama | 11,338 | -182 (-375 to 6) | -1.6 (-3.2 to 0.1) |
| Ishikawa | 11,053 | -262 (-454 to -76) | -2.3 (-3.9 to -0.7) |
| Fukui | 8,034 | -209 (-358 to -61) | -2.5 (-4.3 to -0.8) |
| Yamanashi | 8,448 | -222 (-377 to -68) | -2.6 (-4.3 to -0.8) |
| Nagano | 22,071 | -514 (-843 to -199) | -2.3 (-3.7 to -0.9) |
| Gifu | 19,661 | -613 (-917 to -319) | -3.0 (-4.5 to -1.6) |
| Shizuoka | 36,166 | -1,316 (-1,805 to -833) | -3.5 (-4.8 to -2.3) |
| Aichi | 61,214 | -497 (-1,160 to 177) | -0.8 (-1.9 to 0.3) |
| Mie | 17,939 | -331 (-616 to -55) | -1.8 (-3.3 to -0.3) |
| Shiga | 11,295 | -276 (-472 to -83) | -2.4 (-4.0 to -0.7) |
| Kyoto | 23,267 | -446 (-791 to -116) | -1.9 (-3.3 to -0.5) |
| Osaka | 79,626 | -313 (-1,119 to 468) | -0.4 (-1.4 to 0.6) |
| Hyogo | 51,026 | 195 (-384 to 775) | 0.4 (-0.7 to 1.5) |
| Nara | 12,692 | -310 (-527 to -101) | -2.4 (-4.0 to -0.8) |
| Wakayama | 10,869 | -262 (-451 to -75) | -2.4 (-4.0 to -0.7) |
| Tottori | 6,133 | -229 (-350 to -109) | -3.6 (-5.4 to -1.7) |
| Shimane | 8,295 | -184 (-334 to -33) | -2.2 (-3.9 to -0.4) |
| Okayama | 18,952 | -197 (-478 to 85) | -1.0 (-2.5 to 0.5) |
| Hiroshima | 26,255 | -1,019 (-1,412 to -642) | -3.7 (-5.1 to -2.4) |
| Yamaguchi | 15,998 | -584 (-847 to -333) | -3.5 (-5.0 to -2.0) |
| Tokushima | 8,525 | -272 (-429 to -114) | -3.1 (-4.8 to -1.3) |
| Kagawa | 10,587 | -150 (-334 to 29) | -1.4 (-3.1 to 0.3) |
| Ehime | 15,507 | -455 (-711 to -209) | -2.8 (-4.4 to -1.3) |
| Kochi | 8,578 | -220 (-375 to -64) | -2.5 (-4.2 to -0.7) |
| Fukuoka | 46,226 | -1,409 (-1,981 to -835) | -3.0 (-4.1 to -1.8) |
| Saga | 8,603 | -174 (-327 to -22) | -2.0 (-3.7 to -0.3) |
| Nagasaki | 15,402 | -275 (-523 to -36) | -1.8 (-3.3 to -0.2) |
| Kumamoto | 18,324 | -589 (-881 to -307) | -3.1 (-4.6 to -1.6) |
| Oita | 12,488 | -243 (-458 to -34) | -1.9 (-3.5 to -0.3) |
| Miyazaki | 12,278 | -76 (-285 to 126) | -0.6 (-2.3 to 1.0) |
| Kagoshima | 18,602 | -400 (-686 to -115) | -2.1 (-3.6 to -0.6) |
| Okinawa | 10,857 | -345 (-539 to -152) | -3.1 (-4.7 to -1.4) |
| Japan | 1,190,409 | -22,215 (-37,939 to -6,485) | -1.8 (-3.1 to -0.5) |

**Table S7.** Sensitivity analysis with 5 knots in the interrupted spline component: Number of observed and estimated excess deaths (95% empirical confidence interval) during the period 13 February–31 December 2020 in Japan.

| Prefecture | Total | | |
| --- | --- | --- | --- |
|  | Total deaths | Excess deaths | Percentage excess |
| Hokkaido | 56,790 | -1,079 (-1,764 to -475) | -1.9 (-3.0 to -0.8) |
| Aomori | 15,581 | -530 (-798 to -286) | -3.3 (-4.9 to -1.8) |
| Iwate | 14,846 | -519 (-786 to -273) | -3.4 (-5.0 to -1.8) |
| Miyagi | 21,396 | -674 (-982 to -371) | -3.1 (-4.4 to -1.7) |
| Akita | 13,404 | -295 (-532 to -73) | -2.2 (-3.8 to -0.5) |
| Yamagata | 13,387 | -325 (-562 to -107) | -2.4 (-4.0 to -0.8) |
| Fukushima | 21,167 | -710 (-1,055 to -398) | -3.2 (-4.7 to -1.8) |
| Ibaraki | 28,446 | -746 (-1,174 to -365) | -2.6 (-4.0 to -1.3) |
| Tochigi | 18,811 | -518 (-819 to -248) | -2.7 (-4.2 to -1.3) |
| Gunma | 20,218 | -353 (-668 to -68) | -1.7 (-3.2 to -0.3) |
| Saitama | 61,511 | -548 (-1,288 to 95) | -0.9 (-2.1 to 0.2) |
| Chiba | 53,904 | -549 (-1,202 to 28) | -1.0 (-2.2 to 0.1) |
| Tokyo | 105,503 | -1,060 (-2,089 to -72) | -1.0 (-1.9 to -0.1) |
| Kanagawa | 73,390 | -1,164 (-1,937 to -428) | -1.6 (-2.6 to -0.6) |
| Niigata | 25,746 | -733 (-1,113 to -390) | -2.8 (-4.1 to -1.5) |
| Toyama | 11,338 | -240 (-439 to -54) | -2.1 (-3.7 to -0.5) |
| Ishikawa | 11,053 | -258 (-455 to -72) | -2.3 (-4.0 to -0.7) |
| Fukui | 8,034 | -200 (-358 to -54) | -2.4 (-4.3 to -0.7) |
| Yamanashi | 8,448 | -204 (-367 to -50) | -2.4 (-4.2 to -0.6) |
| Nagano | 22,071 | -547 (-887 to -242) | -2.4 (-3.9 to -1.1) |
| Gifu | 19,661 | -620 (-933 to -340) | -3.1 (-4.5 to -1.7) |
| Shizuoka | 36,166 | -1,234 (-1,758 to -772) | -3.3 (-4.6 to -2.1) |
| Aichi | 61,214 | -481 (-1,161 to 121) | -0.8 (-1.9 to 0.2) |
| Mie | 17,939 | -324 (-622 to -63) | -1.8 (-3.4 to -0.4) |
| Shiga | 11,295 | -261 (-464 to -68) | -2.3 (-3.9 to -0.6) |
| Kyoto | 23,267 | -509 (-865 to -188) | -2.1 (-3.6 to -0.8) |
| Osaka | 79,626 | -106 (-891 to 657) | -0.1 (-1.1 to 0.8) |
| Hyogo | 51,026 | 103 (-508 to 637) | 0.2 (-1.0 to 1.3) |
| Nara | 12,692 | -287 (-513 to -76) | -2.2 (-3.9 to -0.6) |
| Wakayama | 10,869 | -243 (-421 to -56) | -2.2 (-3.7 to -0.5) |
| Tottori | 6,133 | -226 (-345 to -107) | -3.6 (-5.3 to -1.7) |
| Shimane | 8,295 | -187 (-343 to -38) | -2.2 (-4.0 to -0.5) |
| Okayama | 18,952 | -198 (-497 to 73) | -1.0 (-2.6 to 0.4) |
| Hiroshima | 26,255 | -979 (-1,383 to -615) | -3.6 (-5.0 to -2.3) |
| Yamaguchi | 15,998 | -584 (-855 to -336) | -3.5 (-5.1 to -2.1) |
| Tokushima | 8,525 | -276 (-440 to -120) | -3.1 (-4.9 to -1.4) |
| Kagawa | 10,587 | -167 (-346 to 12) | -1.6 (-3.2 to 0.1) |
| Ehime | 15,507 | -465 (-731 to -219) | -2.9 (-4.5 to -1.4) |
| Kochi | 8,578 | -225 (-388 to -70) | -2.6 (-4.3 to -0.8) |
| Fukuoka | 46,226 | -1,452 (-2,038 to -922) | -3.0 (-4.2 to -2.0) |
| Saga | 8,603 | -191 (-341 to -41) | -2.2 (-3.8 to -0.5) |
| Nagasaki | 15,402 | -222 (-477 to 15) | -1.4 (-3.0 to 0.1) |
| Kumamoto | 18,324 | -600 (-901 to -325) | -3.2 (-4.7 to -1.7) |
| Oita | 12,488 | -258 (-481 to -46) | -2.0 (-3.7 to -0.4) |
| Miyazaki | 12,278 | -96 (-298 to 104) | -0.8 (-2.4 to 0.9) |
| Kagoshima | 18,602 | -441 (-743 to -169) | -2.3 (-3.8 to -0.9) |
| Okinawa | 10,857 | -380 (-584 to -187) | -3.4 (-5.1 to -1.7) |
| Japan | 1,190,409 | -22,161 (-36,894 to -9,184) | -1.8 (-3.0 to -0.8) |

**Table S8.** Sensitivity analysis with 6 knots in the interrupted spline component: Number of observed and estimated excess deaths (95% empirical confidence interval) during the period 13 February–31 December 2020 in Japan.

| Prefecture | Total | | |
| --- | --- | --- | --- |
|  | Total deaths | Excess deaths | Percentage excess |
| Hokkaido | 56,790 | -1,029 (-1,708 to -391) | -1.8 (-2.9 to -0.7) |
| Aomori | 15,581 | -515 (-763 to -277) | -3.2 (-4.7 to -1.7) |
| Iwate | 14,846 | -510 (-763 to -262) | -3.3 (-4.9 to -1.7) |
| Miyagi | 21,396 | -670 (-997 to -349) | -3.0 (-4.5 to -1.6) |
| Akita | 13,404 | -303 (-525 to -83) | -2.2 (-3.8 to -0.6) |
| Yamagata | 13,387 | -331 (-564 to -116) | -2.4 (-4.0 to -0.9) |
| Fukushima | 21,167 | -703 (-1,029 to -393) | -3.2 (-4.6 to -1.8) |
| Ibaraki | 28,446 | -764 (-1,136 to -391) | -2.6 (-3.8 to -1.4) |
| Tochigi | 18,811 | -482 (-771 to -200) | -2.5 (-3.9 to -1.1) |
| Gunma | 20,218 | -331 (-626 to -50) | -1.6 (-3.0 to -0.2) |
| Saitama | 61,511 | -556 (-1,293 to 133) | -0.9 (-2.1 to 0.2) |
| Chiba | 53,904 | -549 (-1,202 to 55) | -1.0 (-2.2 to 0.1) |
| Tokyo | 105,503 | -1,070 (-2,085 to -59) | -1.0 (-1.9 to -0.1) |
| Kanagawa | 73,390 | -1,220 (-2,007 to -449) | -1.6 (-2.7 to -0.6) |
| Niigata | 25,746 | -735 (-1,098 to -371) | -2.8 (-4.1 to -1.4) |
| Toyama | 11,338 | -211 (-397 to -28) | -1.8 (-3.4 to -0.2) |
| Ishikawa | 11,053 | -266 (-455 to -80) | -2.3 (-4.0 to -0.7) |
| Fukui | 8,034 | -198 (-346 to -49) | -2.4 (-4.1 to -0.6) |
| Yamanashi | 8,448 | -202 (-356 to -45) | -2.3 (-4.0 to -0.5) |
| Nagano | 22,071 | -562 (-885 to -240) | -2.5 (-3.9 to -1.1) |
| Gifu | 19,661 | -583 (-875 to -305) | -2.9 (-4.3 to -1.5) |
| Shizuoka | 36,166 | -1,237 (-1,697 to -778) | -3.3 (-4.5 to -2.1) |
| Aichi | 61,214 | -438 (-1,119 to 221) | -0.7 (-1.8 to 0.4) |
| Mie | 17,939 | -288 (-548 to -7) | -1.6 (-3.0 to 0.0) |
| Shiga | 11,295 | -256 (-457 to -69) | -2.2 (-3.9 to -0.6) |
| Kyoto | 23,267 | -475 (-812 to -138) | -2.0 (-3.4 to -0.6) |
| Osaka | 79,626 | -186 (-1,005 to 616) | -0.2 (-1.2 to 0.8) |
| Hyogo | 51,026 | 147 (-464 to 716) | 0.3 (-0.9 to 1.4) |
| Nara | 12,692 | -296 (-503 to -88) | -2.3 (-3.8 to -0.7) |
| Wakayama | 10,869 | -248 (-437 to -62) | -2.2 (-3.9 to -0.6) |
| Tottori | 6,133 | -221 (-342 to -102) | -3.5 (-5.3 to -1.6) |
| Shimane | 8,295 | -194 (-343 to -42) | -2.3 (-4.0 to -0.5) |
| Okayama | 18,952 | -177 (-466 to 105) | -0.9 (-2.4 to 0.6) |
| Hiroshima | 26,255 | -989 (-1,374 to -605) | -3.6 (-5.0 to -2.3) |
| Yamaguchi | 15,998 | -579 (-831 to -330) | -3.5 (-4.9 to -2.0) |
| Tokushima | 8,525 | -273 (-432 to -118) | -3.1 (-4.8 to -1.4) |
| Kagawa | 10,587 | -166 (-348 to 13) | -1.5 (-3.2 to 0.1) |
| Ehime | 15,507 | -461 (-708 to -221) | -2.9 (-4.4 to -1.4) |
| Kochi | 8,578 | -224 (-372 to -72) | -2.5 (-4.2 to -0.8) |
| Fukuoka | 46,226 | -1,406 (-1,943 to -888) | -3.0 (-4.0 to -1.9) |
| Saga | 8,603 | -184 (-338 to -32) | -2.1 (-3.8 to -0.4) |
| Nagasaki | 15,402 | -268 (-507 to -36) | -1.7 (-3.2 to -0.2) |
| Kumamoto | 18,324 | -613 (-907 to -328) | -3.2 (-4.7 to -1.8) |
| Oita | 12,488 | -245 (-452 to -39) | -1.9 (-3.5 to -0.3) |
| Miyazaki | 12,278 | -85 (-288 to 115) | -0.7 (-2.3 to 0.9) |
| Kagoshima | 18,602 | -441 (-715 to -148) | -2.3 (-3.7 to -0.8) |
| Okinawa | 10,857 | -359 (-544 to -174) | -3.2 (-4.8 to -1.6) |
| Japan | 1,190,409 | -21,952 (-30,650 to -12,786) | -1.8 (-2.5 to -1.1) |

**Table S9.** The Quasi-Akaike Information Criterion (QAIC) for main model and each sensitivity analysis.

| Prefecture | Main model | Sensitivity analysis | | | | | |
| --- | --- | --- | --- | --- | --- | --- | --- |
|  |  | Without adjusting for influenza activity and ambient temperature | With adjusting for influenza activity | With adjusting for ambient temperature | The number of knots in interrupted spline component | | |
|  |  |  |  |  | 4 | 5 | 6 |
| Hokkaido | 17,730.6 | 17,822.1 | 17,810.8 | 17,744.9 | 17,733.2 | 17,789.9 | 17,791.6 |
| Aomori | 14,636.1 | 14,677.8 | 14,674.4 | 14,634.1 | 14,639.1 | 14,643.6 | 14,645.5 |
| Iwate | 14,798.3 | 14,808.7 | 14,798.8 | 14,804.4 | 14,799.7 | 14,809.9 | 14,809.5 |
| Miyagi | 15,343.6 | 15,365.5 | 15,366.3 | 15,341.9 | 15,345.2 | 15,381.1 | 15,381.9 |
| Akita | 14,386.5 | 14,398.9 | 14,396.2 | 14,386.3 | 14,386.5 | 14,403.2 | 14,401.7 |
| Yamagata | 14,363.2 | 14,380.2 | 14,380.3 | 14,361.1 | 14,365.7 | 14,382.4 | 14,380.7 |
| Fukushima | 15,536.8 | 15,573.1 | 15,570.3 | 15,536.7 | 15,536.6 | 15,564.1 | 15,563.9 |
| Ibaraki | 16,154.7 | 16,278.9 | 16,246.9 | 16,182.4 | 16,157.2 | 16,174.7 | 16,171.5 |
| Tochigi | 14,973.2 | 15,048.0 | 15,046.5 | 14,972.7 | 14,972.6 | 15,008.6 | 15,008.8 |
| Gunma | 15,173.6 | 15,284.5 | 15,282.4 | 15,173.8 | 15,170.2 | 15,198.2 | 15,200.0 |
| Saitama | 18,128.0 | 18,306.5 | 18,288.2 | 18,139.8 | 18,128.6 | 18,154.2 | 18,152.4 |
| Chiba | 17,594.1 | 17,771.4 | 17,747.8 | 17,622.7 | 17,595.7 | 17,613.9 | 17,614.4 |
| Tokyo | 19,529.3 | 19,860.1 | 19,828.1 | 19,563.6 | 19,532.9 | 19,574.7 | 19,579.7 |
| Kanagawa | 18,232.9 | 18,405.7 | 18,379.9 | 18,250.9 | 18,231.9 | 18,283.2 | 18,284.5 |
| Niigata | 15,741.6 | 15,835.9 | 15,811.1 | 15,758.9 | 15,740.2 | 15,785.3 | 15,784.7 |
| Toyama | 13,872.0 | 13,881.0 | 13,879.2 | 13,871.0 | 13,861.2 | 13,883.3 | 13,886.7 |
| Ishikawa | 13,828.1 | 13,855.9 | 13,852.7 | 13,831.0 | 13,825.9 | 13,838.3 | 13,840.7 |
| Fukui | 13,342.4 | 13,375.9 | 13,370.7 | 13,347.0 | 13,342.6 | 13,349.7 | 13,349.3 |
| Yamanashi | 13,346.6 | 13,380.0 | 13,359.1 | 13,357.4 | 13,348.7 | 13,366.4 | 13,368.0 |
| Nagano | 15,362.3 | 15,452.3 | 15,435.3 | 15,370.9 | 15,359.9 | 15,400.2 | 15,397.9 |
| Gifu | 15,126.3 | 15,202.5 | 15,191.8 | 15,136.5 | 15,127.7 | 15,152.4 | 15,153.5 |
| Shizuoka | 16,753.1 | 16,849.1 | 16,844.9 | 16,753.4 | 16,747.1 | 16,796.6 | 16,795.4 |
| Aichi | 17,762.6 | 18,059.3 | 18,041.2 | 17,795.0 | 17,763.5 | 17,797.0 | 17,799.3 |
| Mie | 14,969.4 | 15,032.5 | 15,015.5 | 14,978.0 | 14,967.4 | 15,004.8 | 15,003.8 |
| Shiga | 13,946.2 | 14,005.2 | 14,000.6 | 13,946.5 | 13,948.2 | 13,947.8 | 13,949.2 |
| Kyoto | 15,538.3 | 15,624.7 | 15,618.6 | 15,541.6 | 15,539.8 | 15,577.0 | 15,578.0 |
| Osaka | 18,331.1 | 18,635.7 | 18,617.0 | 18,352.1 | 18,332.1 | 18,367.6 | 18,374.7 |
| Hyogo | 17,429.1 | 17,607.0 | 17,593.9 | 17,438.3 | 17,431.5 | 17,468.6 | 17,470.4 |
| Nara | 14,210.3 | 14,255.4 | 14,249.2 | 14,216.6 | 14,212.1 | 14,230.5 | 14,234.2 |
| Wakayama | 13,939.1 | 13,973.4 | 13,965.7 | 13,938.4 | 13,940.3 | 13,960.5 | 13,963.4 |
| Tottori | 12,744.9 | 12,755.8 | 12,754.9 | 12,744.5 | 12,745.0 | 12,766.1 | 12,768.1 |
| Shimane | 13,274.3 | 13,302.8 | 13,304.9 | 13,272.2 | 13,276.4 | 13,287.0 | 13,287.1 |
| Okayama | 15,143.6 | 15,179.0 | 15,174.0 | 15,146.9 | 15,141.9 | 15,178.8 | 15,180.4 |
| Hiroshima | 16,056.5 | 16,108.6 | 16,105.1 | 16,056.2 | 16,057.5 | 16,100.0 | 16,095.8 |
| Yamaguchi | 14,776.3 | 14,831.4 | 14,825.8 | 14,778.8 | 14,777.5 | 14,792.3 | 14,793.7 |
| Tokushima | 13,477.6 | 13,519.4 | 13,519.3 | 13,475.8 | 13,479.7 | 13,488.0 | 13,489.6 |
| Kagawa | 13,715.6 | 13,756.1 | 13,743.4 | 13,722.2 | 13,716.4 | 13,739.2 | 13,740.2 |
| Ehime | 14,786.5 | 14,837.1 | 14,825.1 | 14,793.8 | 14,786.3 | 14,798.6 | 14,799.4 |
| Kochi | 13,454.1 | 13,513.4 | 13,497.3 | 13,461.8 | 13,456.1 | 13,484.3 | 13,484.5 |
| Fukuoka | 17,235.9 | 17,378.0 | 17,352.4 | 17,250.6 | 17,238.0 | 17,260.5 | 17,262.5 |
| Saga | 13,215.3 | 13,274.6 | 13,259.2 | 13,220.4 | 13,216.3 | 13,233.4 | 13,235.4 |
| Nagasaki | 14,491.2 | 14,567.6 | 14,558.3 | 14,497.5 | 14,490.8 | 14,513.6 | 14,514.6 |
| Kumamoto | 15,150.3 | 15,186.1 | 15,185.2 | 15,149.1 | 15,152.3 | 15,175.1 | 15,173.1 |
| Oita | 14,319.7 | 14,349.0 | 14,339.5 | 14,322.0 | 14,318.7 | 14,343.9 | 14,344.8 |
| Miyazaki | 14,061.0 | 14,119.7 | 14,102.8 | 14,065.5 | 14,059.9 | 14,076.9 | 14,076.5 |
| Kagoshima | 15,204.9 | 15,274.7 | 15,262.4 | 15,212.2 | 15,207.2 | 15,242.0 | 15,242.7 |
| Okinawa | 13,724.4 | 13,781.5 | 13,778.3 | 13,723.7 | 13,727.4 | 13,753.8 | 13,756.9 |
| Japan | 714,911.7 | 718,741.6 | 718,251.4 | 715,240.9 | 716,137.7 | 716,141.6 | 716,180.5 |
